# MiR-202 controls female fecundity by regulating medaka oogenesis

**DOI:** 10.1371/journal.pgen.1007593

**Published:** 2018-09-10

**Authors:** Stéphanie Gay, Jérôme Bugeon, Amine Bouchareb, Laure Henry, Clara Delahaye, Fabrice Legeai, Jérôme Montfort, Aurélie Le Cam, Anne Siegel, Julien Bobe, Violette Thermes

**Affiliations:** 1 LPGP, INRA, Rennes, France; 2 Univ Rennes, INRIA, CNRS, IRISA, Rennes, France; 3 IGEPP, INRA BP35327, Le Rheu, France; University of Wuerzburg, GERMANY

## Abstract

Female gamete production relies on coordinated molecular and cellular processes that occur in the ovary throughout oogenesis. In fish, as in other vertebrates, these processes have been extensively studied both in terms of endocrine/paracrine regulation and protein expression and activity. The role of small non-coding RNAs in the regulation of animal reproduction remains however largely unknown and poorly investigated, despite a growing interest for the importance of miRNAs in a wide variety of biological processes. Here, we analyzed the role of miR-202, a miRNA predominantly expressed in male and female gonads in several vertebrate species. We studied its expression in the medaka ovary and generated a mutant line (using CRISPR/Cas9 genome editing) to determine its importance for reproductive success with special interest for egg production. Our results show that miR-202-5p is the most abundant mature form of the miRNA and that it is expressed in granulosa cells and in the unfertilized egg. The knock out (KO) of *mir-202* gene resulted in a strong phenotype both in terms of number and quality of eggs produced. Mutant females exhibited either no egg production or produced a dramatically reduced number of eggs that could not be fertilized, ultimately leading to no reproductive success. We quantified the size distribution of the oocytes in the ovary of KO females and performed a large-scale transcriptomic analysis approach to identified dysregulated molecular pathways. Together, cellular and molecular analyses indicate that the lack of miR-202 impairs the early steps of oogenesis/folliculogenesis and decreases the number of large (*i*.*e*. vitellogenic) follicles, ultimately leading to dramatically reduced female fecundity. This study sheds new light on the regulatory mechanisms that control the early steps of follicular development, including possible targets of miR-202-5p, and provides the first *in vivo* functional evidence that a gonad-predominant microRNA may have a major role in female reproduction.

## Author summary

The role of small non-coding RNAs in the regulation of animal reproduction remains poorly investigated, despite a growing interest for the importance of miRNAs in a wide variety of biological processes. Here, we analyzed the role of miR-202, a miRNA predominantly expressed in gonads in vertebrate. We studied its expression in the medaka ovary and knocked out the *mir-202* gene to study its importance for reproductive success. We showed that the lack of miR-202 results in the sterility of both females and males. In particular, it led to a drastic reduction of both the number and the quality of eggs produced by females. Mutant females exhibited either no egg production or produced a drastically reduced number of eggs that could not be fertilized, ultimately leading to no reproductive success. Quantitative histological and molecular analyses indicated that *mir-202* KO impairs oocyte development and is also associated with the dysregulation of many genes that are critical for reproduction. This study sheds new light on the regulatory mechanisms that control oogenesis, including possible targets of miR-202-5p, and provides the first *in vivo* functional evidence that a gonad-predominant microRNA may have a major role in female reproduction.

## Introduction

In fish, female fecundity is tightly linked to the proper completion of oogenesis in the ovary, whereby undifferentiated germinal stem cells undergo meiosis, dramatically increase their size and ultimately form the eggs [1]. Such an important differentiation process requires interactions between the oocyte and the surrounding somatic cells (granulosa and theca cells), which together form the ovarian follicles [2]. While the role of endocrine and intra-ovarian factors in this process has been extensively studied, the regulation by the small non-coding RNAs (microRNAs) has received far less attention [3][4].

MicroRNAs (miRNAs) of approximately 22 nucleotides in length, play many different biological functions through the post-transcriptional regulation of protein-coding genes. The contribution of miRNAs to oogenesis was demonstrated in mice with the report of an infertility of Dicer1-deficient females and an arrest of zygotic development of the progeny after the loss of specific maternal miRNAs [5][6]. Numerous studies have highlighted the role played by miRNAs in ovarian development and oogenesis, as shown for miR-224 [7][8][9]. Other studies have reviewed the critical role played by miRNAs in controlling the expression of genes that are essential for folliculogenesis [10–13]. In contrast, data documenting the role played by miRNAs in the fish ovary remain scarce and mainly rely on expression studies. In zebrafish, the differential expression of several miRNAs has been associated with vitellogenesis and follicular development [14]. More specifically, expression and regulation of miR-17 and miR-430b in the ovary suggest a role in follicular development and oocyte maturation. To identify other miRNAs that potentially have an important role in gonads, large-scale differential analyses were performed during sex differentiation, gonadal development or vitellogenesis in various fish species, including rainbow trout (*O*. *mykiss*) [15], zebrafish *(D*. *rerio)* [16][17], Atlantic halibut (*H*. *hippoglossus*) [18], yellow catfish (*P*. *fulvidraco*) [19], Nile tilapia (*O*. *niloticus*) [20][21] and medaka (*O*. *latipes*) [22][23]. In addition, we previously performed a large-scale differential microarray analysis and identified a set of miRNAs that are predominantly expressed in medaka ovary compared to other tissues, which strongly supports a potential role for these miRNAs in fish reproduction.

Among the candidate miRNAs that potentially have an important role in gonads was miR-202 that is predominantly expressed in gonads in fish [22][24][25][26], as well as in other vertebrates, including frog (*X*. *tropicalis*) [27], human (*H*. *s*apiens) [28], mouse (*M*. *musculus*) [29] and chicken (*G*. *gallus*) [30][31]. The role of miR-202 in gonads is not yet understood and most of what is known relies on expression data and some functional studies. In chicken, miR-202 has a sexually dimorphic expression, suggesting a role in sex differentiation [30][32]. In human, miR-202-5p is expressed in sertoli cells and might play a role in the interaction between somatic and germ cells during spermatogenesis [33]. In frog, miR-202-5p expression is enriched in germ cells in the ovary [27]. In mice, miR-202-5p is the predominant miRNA mature form expressed during testis differentiation and is expressed in somatic sertoli cells but not in germ cells [34]. In the adult mouse testis, miR-202-5p is enriched in sertoli cells, but both miR-202-5p and -3p mature forms are expressed in germ cells at similar levels [35]. Notably, knock-out (KO) of *mir-202* in the mouse induces premature differentiation of spermatogonial stem cells and reduces stem cells activity, indicating that miR-202 is a key regulator of spermatogenesis. In fish, miR-202 function in reproduction is much less documented. It has been reported that the miR-202-3p mature form is the predominant arm expressed during gonad development in zebrafish, while miR-202-5p is the predominant arm expressed in adult gonads [36] and in mature oocytes [25]. It has been proposed that this arm-switch may be related to different arm preference between somatic and germ cells, and probably different function for miR-202-5p and miR-202-3p. In medaka, limited evidence suggests an expression of miR-202-5p in oocytes, but no expression in granulosa somatic cells [22]. Despite the existence of significant data on miR-202 expression in gonads, there is still no functional evidence of its role in fish reproduction.

Here, we investigated the role of miR-202 in fish with special attention for its role in the ovary. We thoroughly analyzed its expression in the ovary. We then characterized the reproductive phenotype of *mir-202* KO females generated by CRISPR/Cas9 genome editing. Egg production was thoroughly analyzed to determine the reproductive success of *mir-202* KO females. Quantitative image analysis and large-scale transcriptomic analysis were used to determine ovarian modifications in *mir-202* KO females. We showed that miR-202-5p is the predominant mature form expressed in granulosa cells. The KO of *mir-202* gene resulted in a dramatic decrease of egg number and quality. Furthermore, we showed that miR-202 is involved in the regulation of the early steps of oogenesis/folliculogenesis. Overall, this study sheds new light on the regulatory mechanisms that control the early steps of follicular development in the ovary and provides the first *in vivo* functional evidence that a gonad-predominant microRNA has a major role in female reproduction.

## Results

### MiR-202-5p is highly abundant in granulosa cells

**Fig 1:**
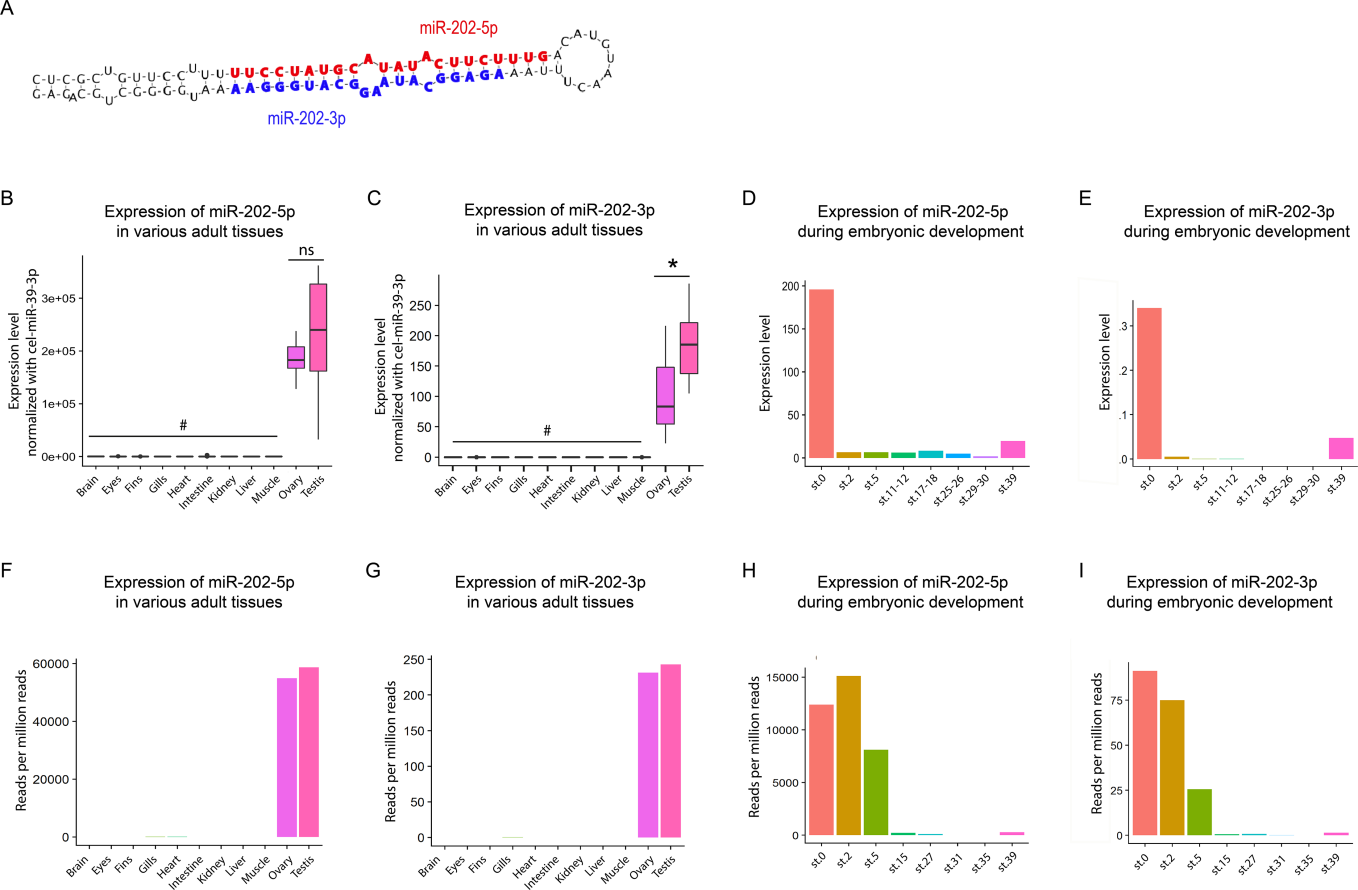
Expression profiles of miR-202-5p and miR-202-3p in adult tissues and during embryonic development. **(A)** Sequence and hairpin secondary structure of pre-mir-202. MiR-202-5p is in red and miR-202-3p is in blue. **(B, C)** Tissue distribution of miR-202-5p and miR-202-3p in eleven adult tissues obtained by TaqMan qRT-PCR (Box plots, n = 8, the ends of the boxes define the 25th and 75th percentiles; a line indicates the median and bars define the 5th and 95th percentiles). Cel-miR-39-3p was used as an external calibrator for normalization. **(D, E)** Expression profiles of miR-202-5p and miR-202-3p during embryonic development obtained by TaqMan qRT-PCR. Pools of embryos at the same stage were used. **(F, I)** Expression profiles of miR-202-5p and miR-202-3p in adult tissues and during embryonic development obtained by small RNA-Seq. *ns*, no significant difference (Student t-test, *p* > 0.05). *#*, expression levels not significantly different from the background signal.

The medaka *mir-202* gene harbors two mature miRNAs sequences, miR-202-5p and miR-202-3p (Fig 1A). Expression levels of both forms were surveyed by quantitative reverse transcription using TaqMan miRNA PCR assay (TaqMan qRT-PCR) in eleven different tissues of adult fish as well as during embryonic development. The miR-202-5p mature form was highly expressed in both gonads (*i*.*e*. ovary and testis), in comparison to other tissues. The expression of miR-202-5p also tended to be higher in testis compared to ovary, although not significantly (Fig 1B). The miR-202-3p mature form exhibited a similar expression profile in adult tissues. The expression level was significantly higher in testis than in ovary and was below detection limits in other tissues (Fig 1C). However, expression levels were approximately 1500 times lower in comparison to miR-202-5p. During embryonic development, the highest expression level of miR-202-5p was detected in non-fertilized eggs at stage 0 (st.0), corresponding to maternal accumulation (Fig 1D). Lower levels of miR-202-5p were detected from one-cell stage (st.2) onwards. A slight increased expression was detected just before hatching (st.39), likely corresponding to zygotic transcription. MiR-202-3p exhibited a similar expression profile during embryonic development and could be detected in unfertilized eggs at levels 500 times lower, while it could not be detected above background levels in other developmental stages (Fig 1E). Expression profiles of miRNA-5p and -3p were also analyzed using small RNA-Seq data sets obtained from the same adult tissues (Fig 1F and 1G), from embryos at different developmental stages (Fig 1H and 1I), as well as from ovarian follicles (S1 Fig). Expression profiles were similar to those obtained by TaqMan qRT-PCR, which further strengthens our findings.

**Fig 2:**
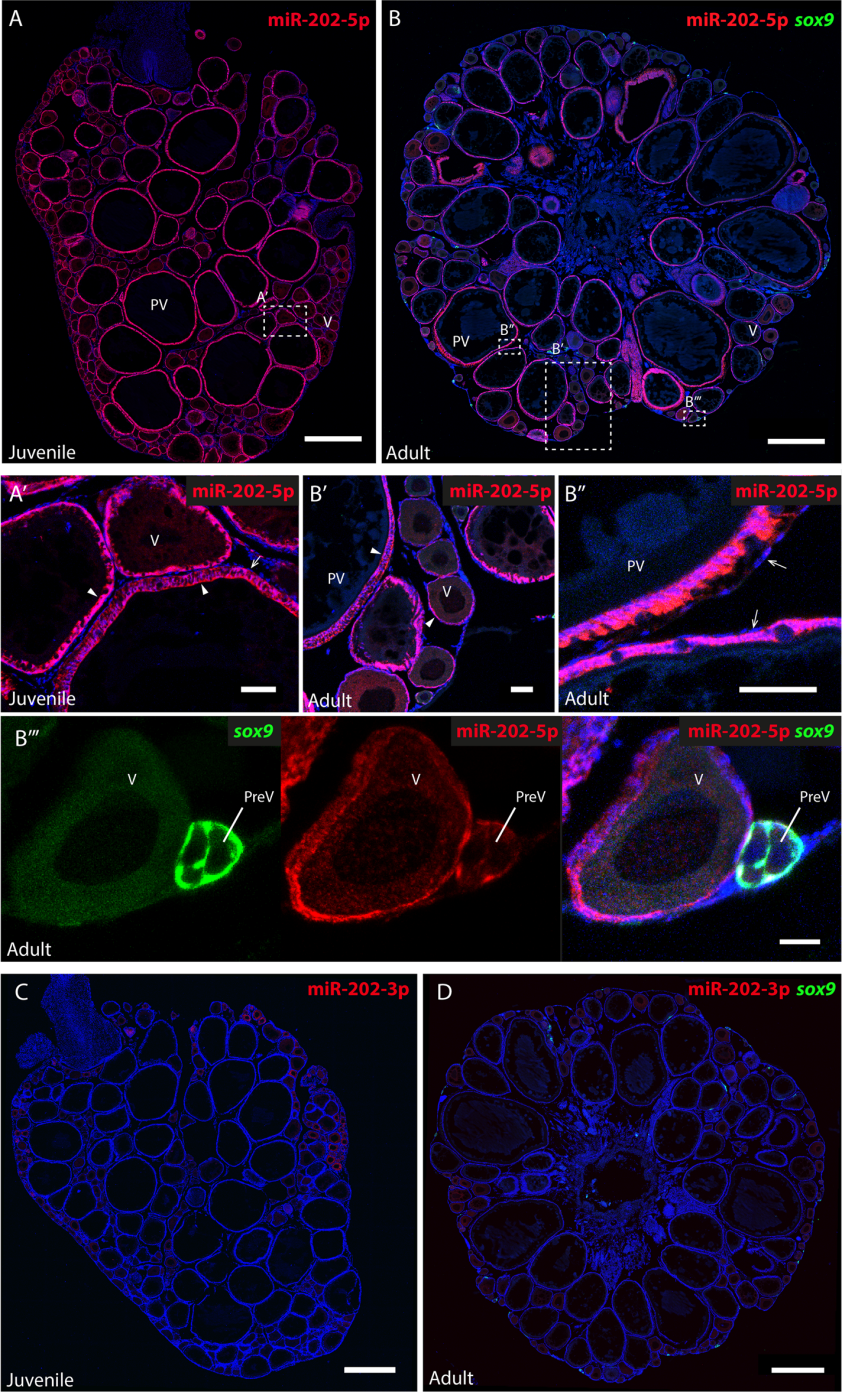
Expression of miR-202-5p and miR-202-3p in the ovary. Fluorescent *in situ* hybridizations (FISH) were performed on sections of ovaries from juvenile and adult medaka females. MiR-202-5p **(A, B)** and miR-202-3p **(C, D)** were detected with specific LNA miRNAs detection probes (in red). Ovaries of adult females were dissected from the transgenic medaka line *Tg*(*sox9b*::EGFP). The somatic GFP+ cells of the germinal cradle, including early granulosa cells, were immunodetected (in green) **(B, D)**. Nuclei are stained with DAPI (in blue). **(A’)** A magnified view of the juvenile ovarian section. **(B’, B” and B”’)** Magnified views of the adult ovarian section. Mir-202-5p is detected in granulosa cells of follicles at all stages in both juvenile and adult ovary (arrowhead), but not in the theca cells (arrow). **(B”’)** MiR-202-5p co-localize with GFP in early granulosa cells surrounding pre-vitellogenic follicles. **(C, D)** Mir-202-3p is not detected in ovaries from juvenile and adult fishes. PreV, pre-vitellogenic follicle; V, vitellogenic follicle; PV, post-vitellogenic follicles. Scale bars: 500 μm (A-D), 50 μm (A’, B’, B”) and 20 μm (B”’).

The cellular expression patterns of miR-202-5p and miR-202-3p were further analyzed in the ovary by fluorescent *in situ* hybridization (FISH) on ovarian sections (Fig 2). Ovaries were dissected either from juvenile females at 3 months old post-fertilization (*i*.*e*. before the first spawning) or from adult females of at least 4 months old post-fertilization (*i*.*e*. reproductively active fishes). In both juvenile and adult ovaries, miR-202-5p was detected in follicles at all vitellogenic and post-vitellogenic stages, in granulosa cells surrounding the oocyte (Fig 2A’ and 2B’, arrowhead), but not in theca cells (Fig 2A’ and 2B”, arrow). Similar ISH expression patterns were observed using frozen sections, which corroborate our results obtained with paraffin sections (S2 Fig). To visualize *sox9*-expressing cells in the germinal cradle, we performed FISH on ovaries from adult transgenic medaka *Tg*(*sox9b*::EGFP). MiR-202-5p was detected in a subset of GFP-positive cells surrounding early pre-vitellogenic follicles (primordial follicles, Fig 2B”’). FISH were also performed using a specific miR-202-3p probe (Fig 2C and 2D) and a scramble control probe (S3 Fig), which revealed no detectable signal above background.

### Knock-out of *mir-202* drastically reduces female fertility

**Fig 3:**
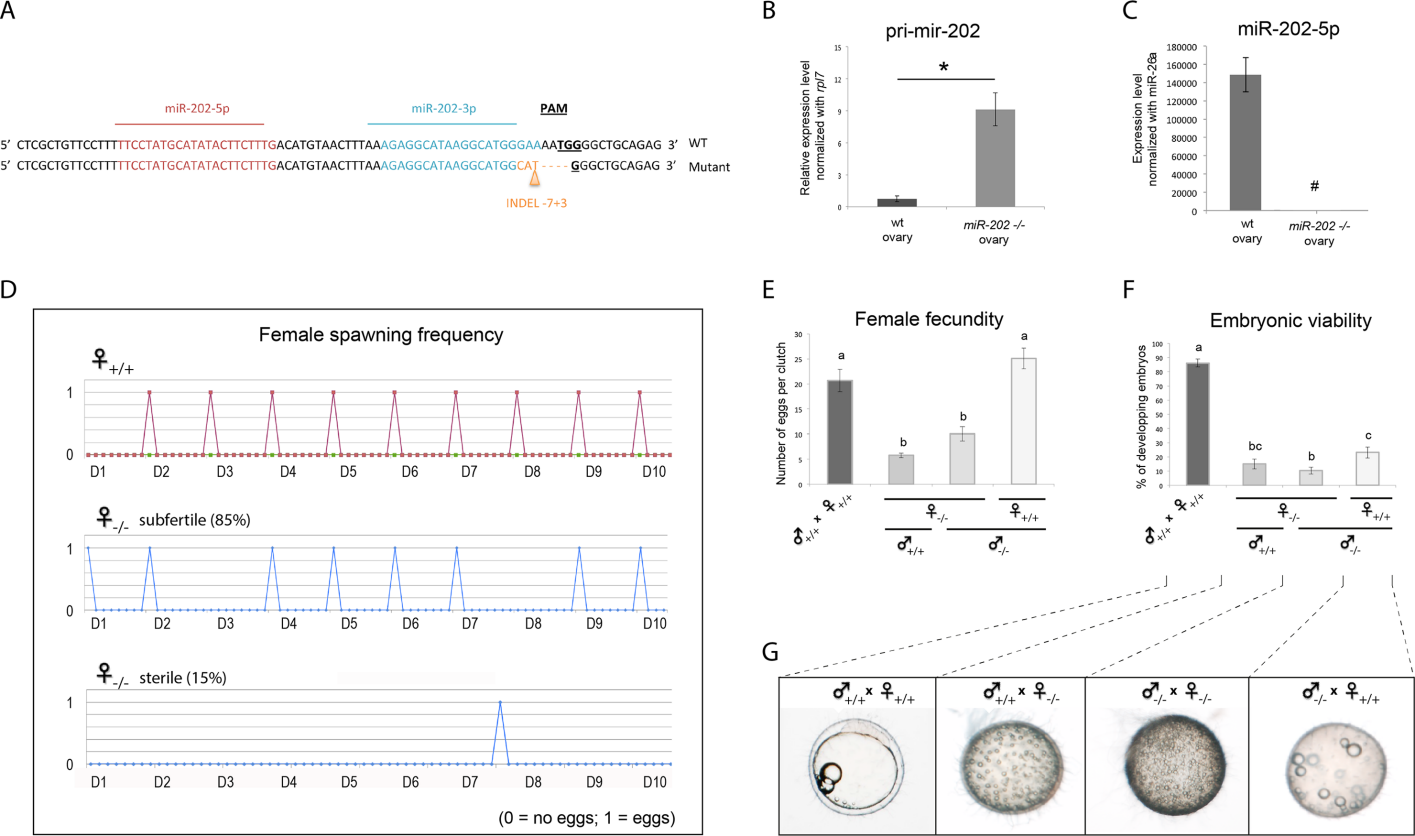
Analysis of the reproductive phenotype of *mir-202 -/-* adult fish. **(A)** Genomic regions of wild-type and mutant fishes showing the INDEL mutations (-7+3) inserted in the miR-202-3p sequence using CRISPR/Cas9 genome engineering. **(B and C)** Expression levels of the pri-mir-202 and miR-202-5p forms in the ovaries of wild-type and homozygotes females (*mir-202 -/-*) were measured by qRT-PCR. **(D)** Spawning frequency of mutant and wild-type females were monitored during ten days. Subfertile females (irregular spawning) and sterile females (only one spawning) represent respectively 85% and 15% of all analyzed females. **(E)** Number of eggs per clutch spawned by wild-type or subfertile females when mated with wild-type or mutant males. **(F)** Embryonic viability of spawn eggs measured by the percentage of eggs that are fertilized and that develop correctly. **(G)** Representative pictures of eggs from the different crosses of wild-type and mutant males and females. Mean values (± SEM) are displayed on the graphs. * indicates expression levels that are significantly different (Mann Whitney test, *p* < 0.05). Different letters indicate a significant difference, as determined by a one-way ANOVA test (Tukey’s post hoc test). *#* expression levels not significantly different from the background signal.

To determine the role of miR-202 on female reproduction, we inactivated the *mir-202* gene using the CRISPR/Cas9 technology. Small insertion/deletion (INDEL) mutations were inserted in the genome in the miR-202-3p mature sequence (Fig 3A). Fishes displaying the same INDEL (-7+3) were selected to establish a mutant line. In ovaries of homozygotes mutant fishes (*mir-202* -/-), the processing of the pri-mir-202 was impaired (Fig 3B) and the miR-202-5p mature form was absent (Fig 3C). We analyzed the sex ratio for heterozygous and homozygous using 66 and 48 fishes, respectively. In both cases, we obtained equilibrated sex ratios as for wild-type fishes. To confirm this phenotype, another mutant line harboring another INDEL mutation (-8) was used. Similarly, we observed equilibrated sex ratios for both heterozygous and homozygous using 31 and 24 fishes, respectively.

We then thoroughly analyzed the reproductive phenotype of *mir-202* -/- adult females of the mutant fish line harboring the INDEL (-7+3). The frequency of spawning was analyzed during ten consecutive cycles (Fig 3D). Two categories of mutant females were distinguishable by different reproductive phenotypes. The mildly affected females (subfertile females), which represented 85% of all mutant females analyzed, displayed an irregular frequency of spawning compared to wild-type females that spawned every day within one hour of the onset of light. The strongly affected females (sterile females), which represented 15% of all mutant females analyzed, spawned only once in ten days. Further analysis of the quantity of spawned eggs (female fecundity) revealed that subfertile females spawned a significantly reduced number of eggs (5.8 eggs per clutch on average) compared to wild-type females (18.8 eggs per clutch on average, Fig 3E). In addition, the viability of embryos (*i*.*e*. capability of eggs to be fertilized and to develop correctly) was dramatically reduced when subfertile females were outcrossed with wild-type males (Fig 3F). Similar results were obtained when subfertile females were mated with mutant males. Conversely, when mutant males were outcrossed with wild-type females, the viability of embryos was also drastically reduced compared to wild-type siblings. Further analysis of siblings revealed that eggs originating from subfertile females mated with either a wild-type or a mutant male could not be fertilized, while eggs originating from wild-type females mated with a mutant male were fertilized but arrested during the first cleavage stages of embryonic development (st.4-5, Fig 3G). The overall reproductive success of mutant females was therefore reduced to 0.83 viable eggs per clutch for subfertile females (*i*.*e*. mutant females that exhibited irregular spawning, reduced fecundity and low embryonic survival) and to 0 viable egg for sterile females that exhibited the most severe phenotype, while wild-type female exhibited an average of 15.3 viable eggs per clutch. To maintain the mutant fish line (INDEL -7+3), heterozygous fishes were thus systematically outcrossed with wild-type fishes at each generation (see Material and Methods). The other mutant line harboring another INDEL mutation (-8) was used to confirm this reproductive phenotype, but this line was not used for further histological and molecular phenotyping analyses. Together, our results show that miR-202 is required for the formation of functional gametes in both female and male gonads.

### *Mir-202* knock-out blocks early follicular growth progression in juvenile females

**Fig 4:**
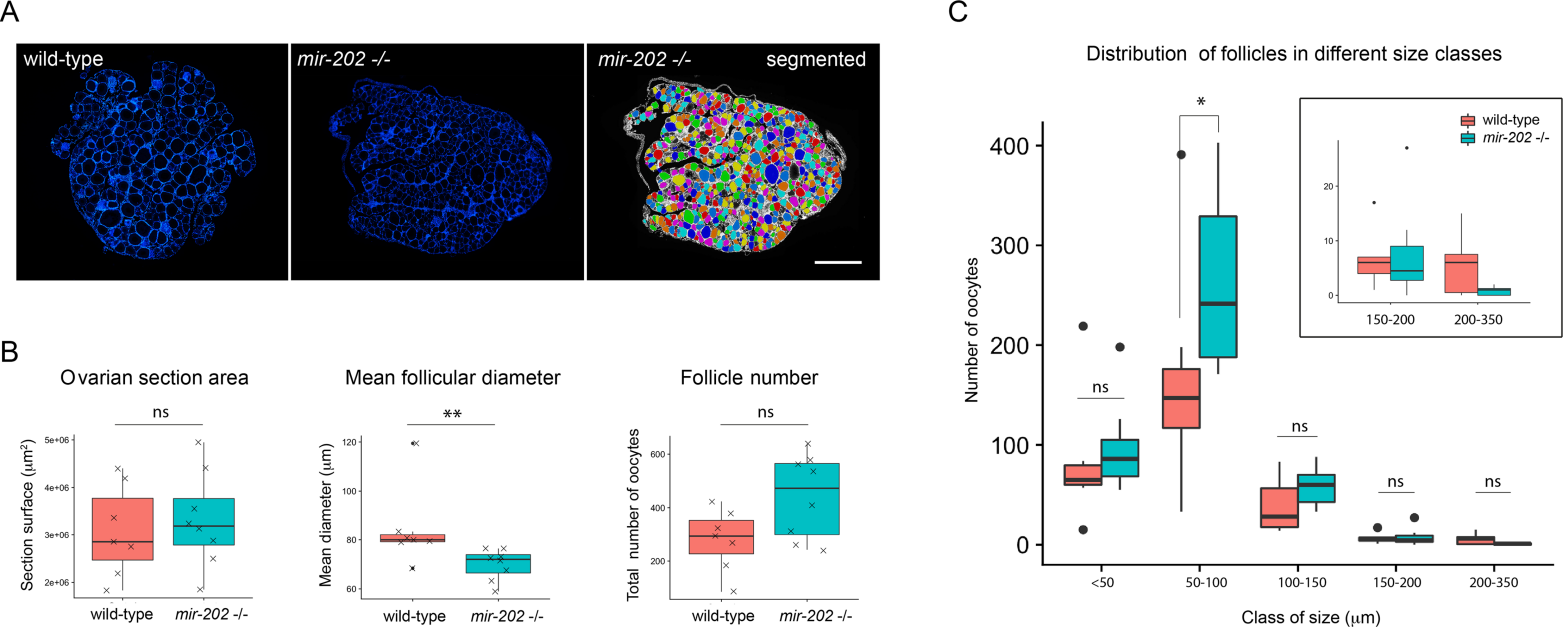
Quantitative image analysis of ovaries from *mir-202 -/-* juvenile females. **(A)** Median sections of ovaries from juvenile wild-type and *mir-202* -/- fishes. All nuclei are stained with DAPI (in blue). Image sections were automatically segmented. **(B)** Section area, mean follicle diameter and follicle number automatically determined. **(C)** Size distribution of follicles on sections. The number of small follicles (50–100 μm) is significantly higher compared to wild-type, while the number of follicles in the larger diameter class (200–350 μm) tends to decrease. Box plots are displayed on graphs for wild-type (n = 7) and *mir-202 -/-* (n = 8) juvenile females (in red and green, respectively). The ends of the boxes define the 25th and 75th percentiles; a line indicates the median and bars define the 5th and 95th percentiles. Individual values are shown for the graphs of panel B. Asterisks indicate significant differences (* *p* < 0.05 and ** *p* < 0.01, Mann Whitney test). Scale bar: 500 μm.

We further analyzed the role of miR-202 during oogenesis in juvenile females (*i*.*e*. before the first spawning). To this aim, the follicular content of wild-type and mutant ovaries were analyzed and compared. For each ovary, the number and size of follicles were determined on the median ovarian section. Nuclei of somatic cells, including granulosa cells surrounding each oocyte, were stained with DAPI, which allowed delineating all follicles and automatically individualizing them, using a computational automatic segmentation procedure (Fig 4A). The section area, the mean follicle diameter and the number of follicles were determined (Fig 4B). On *mir-202* -/- ovarian sections, the mean follicular diameter was significantly reduced (70 μm), as compared to wild-type ovarian sections (80μm), and the number of follicles appeared higher (500μm) as compared to wild-type (300μm), although not significantly. These data suggest an impairment of the early follicular growth in juvenile mutant ovaries.

The size distribution of follicles on sections was thoroughly analyzed in mutant and wild-type ovaries. Follicles were classified according to their diameter into five different classes (<50μm, 50–100μm, 100–150μm, 150–200μm and 200–350μm) (Fig 4C). Comparison of the resulting profiles revealed different size distribution profiles in mutant and wild-type ovaries. In mutant ovarian sections, the number of small follicles appeared higher compared to wild-type, with a significant increased in the 50–100μm size class, whereas the number of follicles in the larger diameter class (200–350μm) tended to decrease, although not significantly. This clearly suggests an arrest of early follicular growth progression in juvenile *mir-202* -/- females, leading to an accumulation of small and medium oocytes (*i*.*e*. pre-vitellogenic and early-vitellogenic oocytes) to the detriment of larger late-vitellogenic and maturing follicles.

### *Mir-202* knock-out impairs follicles growth in subfertile and sterile adult females

**Fig 5:**
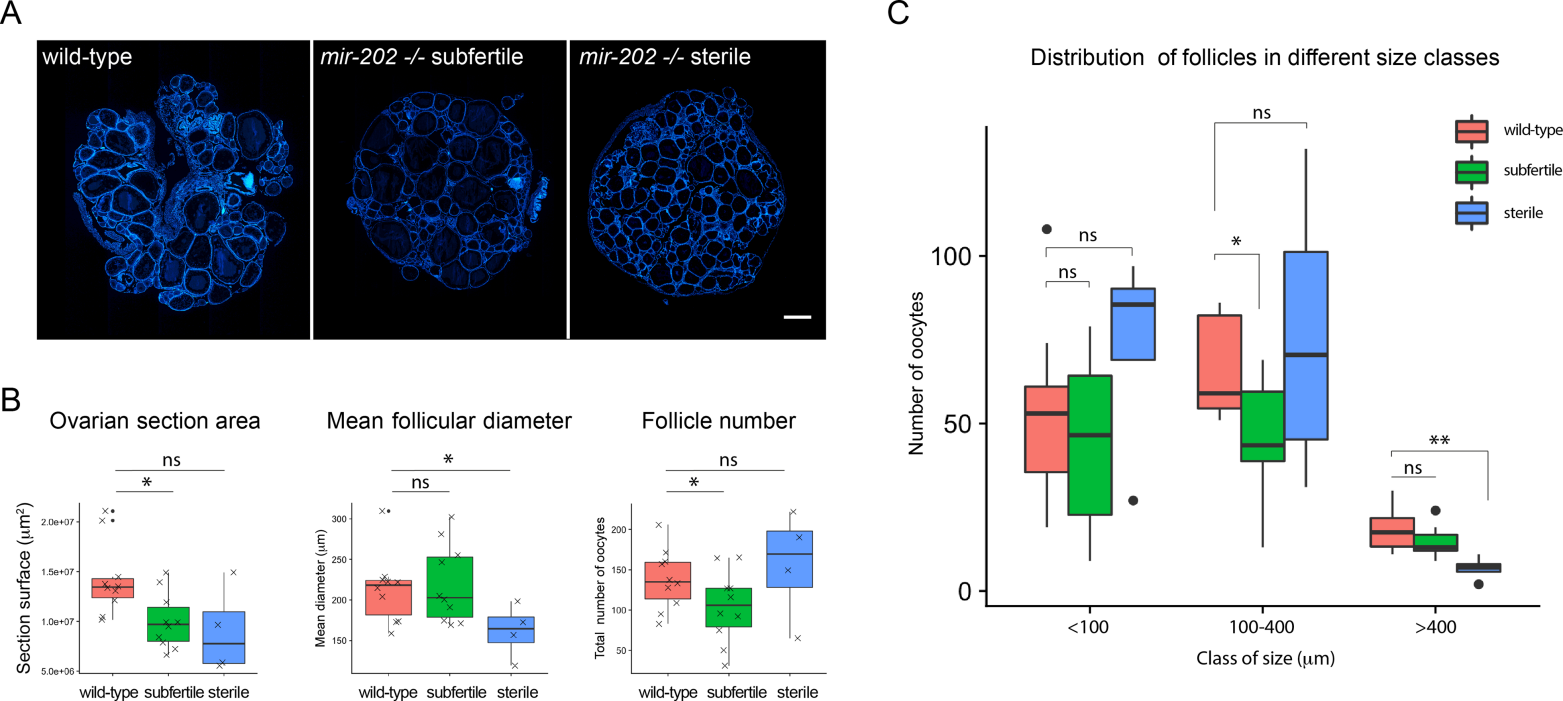
Quantitative image analysis of ovaries from sterile and subfertile *mir-202 -/-* adult females. **(A)** Median sections of ovaries from wild-type, sterile and subfertile mutant females. All nuclei are stained with DAPI (in blue). Image sections were automatically segmented. **(B)** Section area, mean follicle diameter and follicle number automatically determined. **(C)** Size distribution of follicles on sections. In ovaries from sterile females, the number of large follicles (>400 μm) is significantly reduced, while small follicles (<100 μm) tend to accumulate. In the ovary from subfertile females, the number of medium follicles (100–400 μm) is significantly reduced, but small size follicles (<100 μm) do not accumulate. Box plots are displayed on graphs for wild-type (n = 10), *mir-202 -/-* subfertile (n = 10) and *mir-202 -/-* sterile (n = 4) adult females (in red, green and blue, respectively). The ends of the boxes define the 25th and 75th percentiles; a line indicates the median and bars define the 5th and 95th percentiles. Individual values are shown for graphs of panel B. Asterisks indicate significant differences (* *p* < 0.05 and ** *p* < 0.01, Mann Whitney test). Scale bar: 500 μm.

The role of miR-202 during oogenesis was analyzed at the adult stage (reproductively active fish), in both subfertile and sterile females (Fig 5A). Section areas, mean follicular diameters and numbers of follicles were measured on median sections. The follicular size distribution was also analyzed. Follicles were classified according to their diameter into three different size classes based on their diameter: small (<100μm), medium (100–400μm) and large (400–1200μm). In sterile females, ovaries displayed a significant decrease of the mean follicular diameter (170μm) in comparison to wild-type ovaries (220μm), but similar numbers of follicles were found in both cases. Consistently, ovarian sections were significantly reduced in sterile females compared to wild-type (Fig 5B). The analysis of the size distributions also revealed distinct profiles in wild-types and sterile females. The number of large follicles (>400μm) was significantly reduced in sterile females, while small follicles (<100μm) tended to accumulate, although not significantly (Fig 5C), suggesting an accumulation of small follicles to the detriment of large follicles. These observations reveal a strong impairment of the early steps of the follicular growth in sterile females, similarly to juvenile mutant females analyzed during the first reproductive cycle (see above).

In subfertile females, which exhibited a milder reproductive phenotype (*i*.*e*. occurrence of spawning but a reduced number of eggs and reduced egg survival), ovarian sections displayed a significantly reduced number of follicles. This was associated with a significant reduction of ovarian section areas (Fig 5B). No modification of the mean follicular diameter was however observed, suggesting that although fewer follicles were engaged into growth, they were all able to reach their correct final size. This was supported by the follicular size distribution profiles, which showed a significant and important reduction of the number of medium-size follicles (100–400μm in diameter) in subfertile females compared to wild-type, and no accumulation of small-size follicles (<100μm in diameter, Fig 5C). These data likely reflect a milder effect of the miR-202 deficiency on follicular growth in subfertile adult females compared to sterile adult fishes, indicating a partial recovery of the phenotype at this stage.

### Large-scale transcriptomic analysis of juvenile ovaries reveals major dysregulation of key ovarian genes

**Fig 6:**
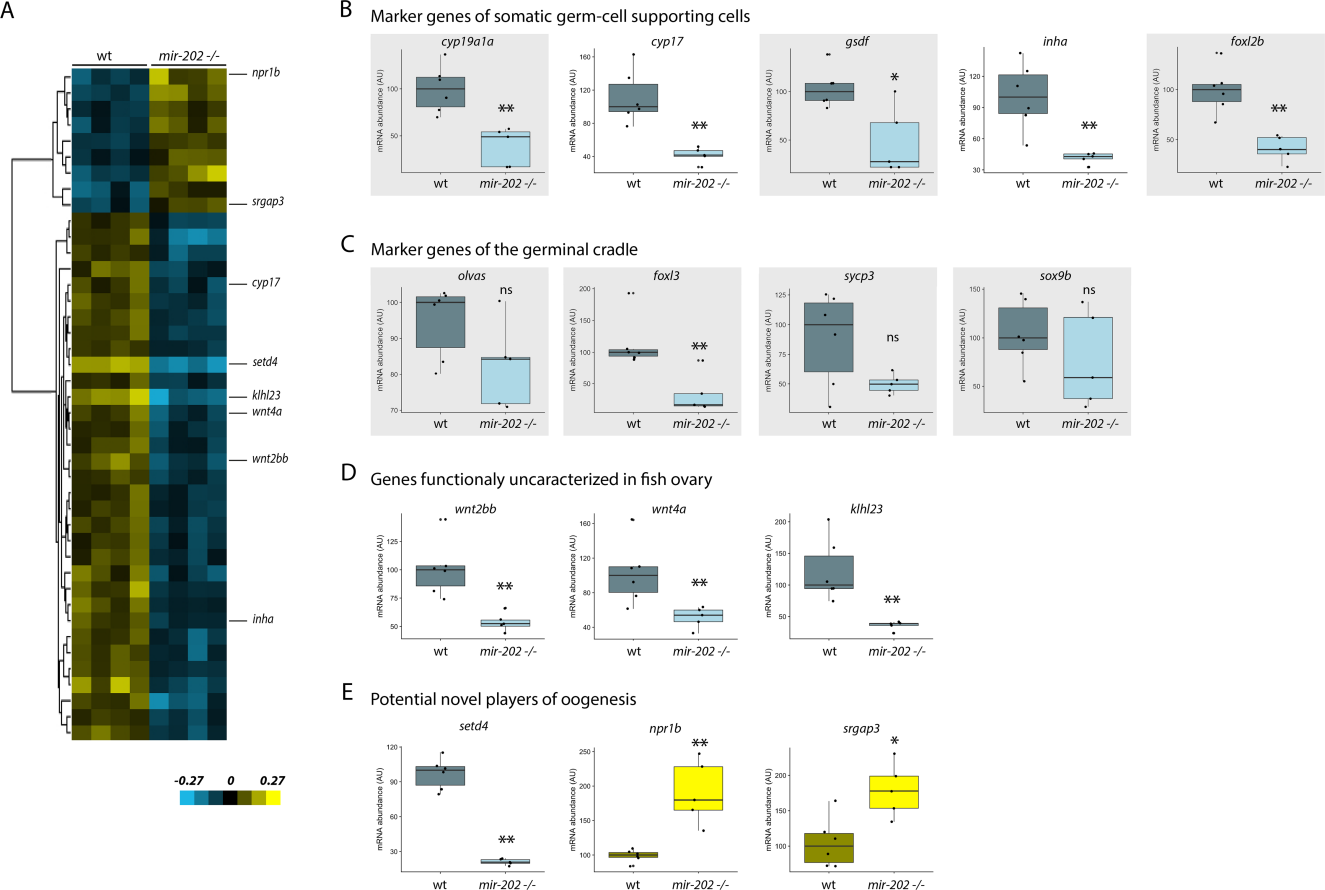
mRNA expression profiles of ovarian genes in *mir-202 -/-* females. A transcriptomic analysis was performed on ovaries of wild-type and *mir-202* -/- juvenile females (*i*.*e*. before the first spawning). The most differentially expressed genes were analyzed by qRT-PCR along with other major genes involved in fish oogenesis, selected from the literature (grey boxes). **(A)** Supervised cluster of the differentially expressed genes (at least 2 fold-change, p < 0.05 after Benjamini-Hochberg correction). Data are median-centered. **(B)** Key ovarian genes expressed in somatic germ-cell supporting cells of the follicles. **(C)** Key ovarian genes expressed in the germinal cradle. **(D)** Genes that are known to be expressed in the ovary of several vertebrates but whose function in the ovary remain poorly investigated. **(E)** Genes that have never been previously described as key ovarian genes and that are potential novel molecular players of oogenesis. Expression levels were measured in triplicates. AU: arbitrary units. Expression levels in wild-types were set at 100%. Box plots are displayed on graphs for wild-type (n = 6) and *mir-202 -/-* (n = 5) juvenile females. The ends of the boxes define the 25th and 75th percentiles; a line indicates the median and bars define the 5th and 95th percentiles. Individual values are shown. The *rpl7* gene was used for normalization. Asterisks indicate significant differences (* *p* < 0.05 and ** *p* < 0.01, Mann Whitney test).

To get further insight into the molecular pathways that are affected in absence of miR-202, we performed a large-scale transcriptomic analysis on ovaries of wild-type and *mir-202* -/- females. This analysis was conducted on ovaries from juvenile females, before the first spawning, when ovaries are more homogeneous and display a small range of follicular sizes (from 30 to 350 μm in diameter), compared to ovaries from reproductively active adult fish that display a large range of follicular sizes (from 30 to 1100 μm in diameter, see Figs 4 and 5). After retaining only genes exhibiting at least a 2-fold change in gene expression, we identified 42 differentially expressed genes (corresponding to 52 probes), including 9 genes up-regulated in mutants and 33 genes down-regulated in mutants (p < 0.05, after Benjamini-Hochberg correction, Fig 6A and S1 Table). The most differentially expressed genes (*cyp17*, *inha*, *wnt2bb*, *wnt4a*, *klhl23*, *setd4*, *npr1b and* srgap3) were analyzed by quantitative RT-PCR (qRT-PCR) along with other major genes (*cyp19a1a*, *gsdf*, *foxl2b*, *olvas*, *foxl3*, *sycp3 and sox9b*) involved in fish oogenesis and selected from the literature. All genes that were identified in the microarray analysis were also significantly differentially expressed in the qRT-PCR analysis. Results are shown on Fig 6 and described bellow.

### Down-regulation of marker genes expressed in the somatic germ-cell supporting cells during folliculogenesis (Fig 6B)

Expression analysis revealed a marked expression decrease of two genes encoding key steroidogenic enzymes (*cyp19a1a* and *cyp17*). *Cyp19a1a* (also known as aromatase) is an ovarian-predominant steroidogenic enzyme that mediates estradiol (E2) production and therefore plays a major role in both sex differentiation and vitellogenesis [3]. An even more pronounced under-expression was observed for *cyp17* that acts upstream of *cyp19a1a*. Consistently, data obtained from microarray analysis also revealed the down-regulation of the steroidogenic acute regulatory gene (*star*, S1 Table). *Star* is involved in the cholesterol shuttling across the inner mitochondrial membrane, which is a rate-limiting step of steroidogenesis [37][38]. A decrease in *star* expression indicates an overall decrease in steroid hormones production in the ovary. We also observed the down-regulation of *gsdf* and inhibin, two members of the TGFβ family, in the ovary of mutant females. *Gsdf* is known to be expressed in granulosa cells and to be involved in the maintenance and maturation of these cells [39][40]. Inhibin consists of a unique α subunit and an activin β subunit. In the zebrafish ovary, members of the activin-inhibin-follistatin system exhibit dynamic changes in expression during folliculogenesis and the α subunit (*inha*) is expressed during vitellogenesis, especially during final oocyte growth (*i*.*e*. prior to final oocyte maturation)[41]. Finally, qRT-PCR analysis revealed a significant down-regulation of *foxl2b*, the only *foxl2* copy retained in medaka after teleost-specific genome duplication [42]. In medaka, *foxl2b* encoding the Foxl2b transcription factor that is a mainly expressed in the female gonad, more specifically in the somatic cells surrounding germ cells in the germinal cradle. At later stages, *foxl2b* continues to be expressed in granulosa cells and in a minority of theca cells in pre-vitellogenic and vitellogenic follicles, but expression ceased in post-vitellogenic follicles [43][44]. In mice, *Foxl2* is known as a key regulator of oogenesis and plays a critical role in ovarian differentiation [45][46][47].

### Down-regulation of marker genes expressed within the germinal cradle (Fig 6C)

We analyzed the expression of the medaka *vasa*-like gene (*olvas*), *foxl3* (a *foxl2*-relative), *sycp3 and sox9b*. *Olvas* is a specific marker of early germinal stem cells, which are nested in the cords of *sox9b*-expressing cells (*i*.*e*. the somatic pre-granulosa cells), referred to as the germinal cradle [48][4]. *Foxl3* is expressed in germ-cells undergoing cystic division prior entry into meiosis, while *sycp3* is expressed in germ cells undergoing meiosis [4][49]. Quantitative RT-PCR revealed a significant down-regulation of *foxl3* in ovary of mutant females, while down-regulation of *sycp3* and *olvas* was not significant. In contrast, the expression of *sox9b* was similar in wild-type and mutant ovaries. These observations indicate a significant reduction of the germ cell activity in the germinal cradle of mutant ovaries, whereas no modification of the surrounding somatic cells was observed as suggested by *sox9* expression.

### Down-regulation of other genes that belong to the WNT and KELCH families (Fig 6D)

Among the most differentially expressed genes were two other genes coding for proteins of the WNT (wingless-related integration site) signaling pathway, *wnt2bb* and *wnt4a*. In the ovary of mutant females, qRT-PCR revealed a clear and significant down-regulation of both *wnt2bb* and *wnt4a*. *Wnt4a* is expressed in fish ovary, while the expression of *wnt2bb* remains uncharacterized to date [50]. The microarray analysis also revealed a dramatic down-regulation of *klhl23*, a member of the KELCH Like Family. Kelch proteins were initially characterized due to their important role in oocyte-somatic cells communication during drosophila oogenesis. Several members of this large family (over 30 members) are known to be expressed in the ovary of several vertebrates even though they remain poorly studied [51].

### Identification of novel molecular players of oogenesis (Fig 6E)

Finally, transcriptomic analysis revealed three other differentially expressed genes (*setd4*, *npr1b* and *srgap3*) that have, to our knowledge, never been previously described as molecular players of oogenesis. The set domain-containing protein 4 gene (*setd4*) displayed a significantly reduced expression in the ovary of mutant females. *Setd4* encodes for a recently characterized methyltransferase involved in breast cancer cell proliferation [52]. While the role of *setd4* in the ovary remains unknown, a recent report demonstrated its role in the regulation of cell quiescence during the diapause of artemia embryos [53]. The natriuretic peptide receptor 1b gene (*npr1b*) exhibited a marked over-expression in mutant juvenile females. Although the role for *npr1b* in oogenesis has never been reported, the mouse *nrp2* gene has already been detected in granulosa cells and shown to maintain meiotic arrest of oocytes in mouse ovary [54]. The slit-robo Rho GTPase activating protein 3 (*srgap3*) gene displayed a milder, yet significant, over-expression. Along with a well-established function in axon guidance, several studies suggested that the SLIT/ROBO pathway could also have important functions in the reproductive system [55]. Together, the differential expression of *setd4*, *npr1b* and *srgap3* in mutant juvenile females and the severe reproductive phenotypes in mutant adult females suggest these genes are important novel molecular players of oogenesis.

### *In silico* prediction of miR-202-5p targets in the ovary

The target prediction analysis resulted in the identification of 772 transcripts by both miRanda and TargetScan (S2 Table). Among those 772 transcripts, 557 were expressed in the medaka ovary according to our microarray data (present at levels above background in at least 3 out of 4 samples in at least 1 of the 2 experimental groups). These transcripts corresponded to 533 different genes including 428 annotated genes according to Ensembl. Among those genes, we found 18 genes displaying a putative binding site for miR-202-5p in their 3’UTR region. This gene set included *stat3* (signal transducer and activator of transcription 3) and *clockb* (clock circadian regulator b).

## Discussion

Most of our current understanding of the regulatory mechanisms of oogenesis in fish is due to several decades of research on hormones, secreted factors or intrinsic signaling pathways. However, miRNAs are well known for their role in many physiological processes through post-transcriptional gene regulations. Here, we showed that this fundamental class of molecules, endowed with pleiotropic functions, is also necessary for oogenesis in the medaka ovary. In particular, we showed that miR-202 plays a key role in early follicular recruitment and growth, and ultimately in the female reproductive success, as shown by the severe phenotype of *mir-202* KO fishes.

### MiR-202-5p is the predominant mature form and is expressed in granulosa cells

Quantitative expression analyses of miR-202-5p and -3p revealed that miR-202-5p is the predominant form expressed in medaka ovary, while the miR-202-3p form is detected at much lower levels. Generally, RNA duplexes of ∼22 nucleotides are transitorily accumulated during miRNA biogenesis and one strand of the duplex is degraded, while the other remains bound into the silencing complex for post-transcriptional regulations [56]. It is therefore likely that miR-202-5p is the main biologically active mature form in granulosa cells, although we cannot totally exclude a role for miR-202-3p. Quantitative expression analyses also showed that miR-202-5p is predominantly expressed in medaka gonads and in unfertilized eggs, as compared to other tissues and embryonic stages. These results are similar to previous observations in medaka and zebrafish gonads [25][22]. For protein-coding genes, such a predominant expression in gonads is usually associated with a major role in reproduction, as illustrated by many maternal-effect genes [57].

Further analysis of the cellular expression pattern in ovary revealed a restricted expression of miR-202-5p in ovarian somatic granulosa cells, as shown by ISH using both paraffin or frozen ovarian sections (Fig 2 and S2 Fig). By contrast, a previous study in medaka reported no expression of miR-202-5p in granulosa cells using ISH on frozen ovarian section [22]. This discrepancy could be due to the use of different hybridization temperatures for ISH. Here, we used a hybridization temperature of 53°C (*i*.*e*. 30°C below the given RNA Tm as specifically recommended). In Qiu *et al*., a much higher hybridization temperature of 65°C was used, which could explain the absence of signal in granulosa cells [22]. These authors however reported a weak signal of miR-202-5p in oocytes at very early stages (st.I and st.II), but did not provide any negative control data to confirm the specificity of the staining. In the present study we used a scramble control probe to estimate the background and no specific signal could be detected in these cells (S2 and S3 Figs). Together, these observations show that miR-202-5p is highly expressed in granulosa cells in the medaka ovary. Although not detected by ISH, miR-202-5p could however be present at low levels in early oocytes and subsequently be accumulated in oocytes at later stages. This would be consistent with the significant expression level of miR-202-5p detected in unfertilized eggs using TaqMan qRT-PCR and small RNA-Seq (Fig 1 and S2 Fig), and similar to what was previously reported in zebrafish [25][58].

### *Mir-202* gene is required for both male and female reproductive success, but not for sex determination

Here, we observed that the inactivation of *mir-202* gene in medaka does not lead to any modification of the sex ratio, since mutant siblings give rise to 50% of adult males and 50% of adult females. This rules out the possibility of a key role of miR-202 in sex-determination. It should however be stressed that the *mir-202* KO results in the sterility of both females and males. More particularly, when *mir-202* KO males were crossed with wild-type females, embryonic viability was dramatically reduced with most embryos arrested during the first cleavage stages (st.4-5). To our knowledge, this phenotypic defect of male reproduction has never been reported in fish, and it clearly indicates that miR-202 is also required for spermatogenesis. This is in agreement with results obtained after *mir-202* KO in cultured spermatogonial stem cell in mice, indicating a role in the control of the cellular proliferation/differentiation balance [59]. Further studies are now required to unravel the precise role of miR-202 in fish testis during spermatogenesis.

### Females lacking miR-202 produce eggs that cannot be fertilized

In this study, we thoroughly analyzed the severe phenotype displayed by *mir-202* KO females. The overall reproductive success of mutant females was reduced to 0.83 (for subfertile females) or, even more, to 0 viable eggs per clutch (for sterile females), compared to 15.3 viable eggs per clutch for wild-type females. This phenotype is the consequence of both a reduced fecundity (*i*.*e*. reduced number of spawned eggs) and reduced egg viability. Indeed, mutant females produced a large proportion of eggs that could not be fertilized, subsequently resulting in no embryonic development. During folliculogenesis, the somatic cells that surround the growing oocyte (granulosa and theca cells) contribute to the formation of the micropyle, which has a pivotal role in egg fertilization [60]. It is thus possible that *mir-202* gene, which is expressed in granulosa cells throughout folliculogenesis, contributes to the formation of the micropyle. However, we cannot exclude a role of miR-202 in oocytes during growth and maturation follicular steps, given the possible accumulation of miR-202 inside the oocyte, which could lead to the laying of abnormal and unfertilizable eggs.

### *Mir-202* knock-out severely affects early folliculogenesis and female fecundity

The very low fecundity of *mir-202* KO females strongly indicates that miR-202 plays an important role in oogenesis in the ovary. One of the most remarkable features observed in mutant ovaries was the increased number of pre-vitellogenic follicles. Along with this phenotype, we observed a reduced expression of *gsdf* (a member of the TGFβ family) and *foxl2b* (encoding the Foxl2b transcription factor). Both *gsdf* and *foxl2b* genes are expressed in granulosa and are well known for their role as key regulators of early oogenesis/folliculogenesis [42][61][62]. Consistently, we also observed the down-regulation of downstream key genes, including steroidogenic genes (*star*, *cyp19a1a*, and *cyp17*) and the TGFβ family member inhibin. The formers are involved in estrogen synthesis in ovarian follicles, while inhibin plays a critical role during follicular development in zebrafish [63]. Down-regulation of these factors thus strongly indicates that miR-202 likely controls the early development of granulosa cells (*i*.*e*. granulosa proliferation and/or differentiation) through the regulation of *gsdf* and *foxl2b*, and ultimately leads to an impaired folliculogenesis. Similarly, previous quantitative expression analysis study in zebrafish reported a differential expression of miR-202 during folliculogenesis, indicating a possible role in follicle activation [58]. Further analysis of the proliferation and differentiation states of granulosa cells are now needed to further understanding the role of miR-202 during folliculogenesis.

Of particular interest is also the down-regulation of *gsdf* in *mir-202* mutant ovaries. Along with its well-established key role in the male-determination in medaka, the Gsdf factor is also thought to be necessary for normal ovarian development in female [64][39]. Indeed, *gsdf* inactivation not only impairs male differentiation, but also leads to a severely reduced female fecundity up to a total infertility for the most severe cases. In such mutants, the number of pre-vitellogenic follicles is abnormally high [39]. Except the male-determination phenotypic defect, many features of the *gsdf* mutant are thus very similar to that observed for *mir-202* KO females, including the pre-vitellogenic follicles accumulation. We hypothesize that miR-202 promotes *gsdf* expression in the ovary and boosts its action on follicular development. In any case, such hypothesis remains to be confirmed and molecular mechanisms underlying the interaction between miR-202-5p and *gsdf* remain to be identified.

### *Mir-202* knock-out impairs germ cells activity in the germinal cradle

In the medaka ovary, the germinal cradle is composed somatic *sox9b*-expressing cells surrounding mitotic and meiotic germ cells, as well as primordial oogonia barely engage into folliculogenesis [4]. Our results show that *mir-202* is expressed in the germinal cradle, in a subset of *sox9b*-expressing cells surrounding primordial oogonia, which together likely correspond to primordial follicles. Interestingly, no modification of *sox9b* expression was observed in *mir-202* KO ovaries. In mice, previous studies have shown that *sox9* acts upstream of *mir-202* and regulates its expression in granulosa cells [65]. It is thus possible that *sox9b* acts upstream of *mir-202* in medaka as well. However, molecular mechanisms that regulate the expression of *mir-202* in follicles at later stages, in vitellogenic and post-vitellogenic follicles, remain to be determined since *sox9b* is no longer expressed at these stages.

We also observed, in juvenile ovaries, a down-regulation of *foxl3* that is expressed in germ cells undergoing cystic divisions prior entry into meiosis and, to a lesser extent, of *sycp3* that is expressed in germ cells undergoing meiosis [4][49]. These down-regulations indicate that miR-202 is necessary for cystic division and meiosis of germ cells within the germinal cradle, in addition to the correct follicular development (as discussed above). By contrast, miR-202 was shown to be essential for stem cells maintenance and to prevent spermatogonial stem cells (SSCs) from premature differentiation in mouse testis [66]. It will thus be very informative in the future to determine the role of miR-202 in the maintenance and fate determination of stem cells in the testis of medaka. Nevertheless, it must be stressed that no expression of *mir-202* was detected in early oocytes, we however cannot exclude that *mir-202* is barely expressed in early oocytes and could thus be involved in the regulation of germ cells mitosis and meiosis. An other possibility is that miR-202-5p acts through cellular interactions between germinal and somatic cells that are thought to be important for meiosis progression [67][68][51]. Any early defect of oocytes may thus occur in the context of granulosa dysfunction, including the onset of meiosis. One possibility is that miR-202 depletion in the granulosa affects oocyte-somatic cells communications, as suggested by the down-regulation of one member of the *KELCH* family, which has an important role in oocyte-somatic cell communication in drosophila [51]. This hypothesis is also supported by the dysregulation of the *npr1b* receptor and the intracellular effector *srgap3* of the SLIT/ROBO signaling pathway, which could both also mediate cellular communications in the ovary [54][55].

### *Stat3* and *clockb* are predicted target genes of miR-202-5p in the ovary

To understand the action mechanisms of miR-202, we searched for direct molecular targets of miR-202-5p *in silico*. Target prediction for miR-202-5p resulted in the identification of various putative targets mRNAs expressed in medaka ovary, including *stat3* and *clockb*. The co-expression of these genes and miR-202-5p supports a potential interaction between the miR-202-5p and these potential targets, although further validation of these molecular interactions is needed. However, the potential regulation of Stat3 and Clockb proteins would undoubtedly provide new insights into the comprehension of the regulatory mechanisms of fish fecundity.

Multiple lines of evidence place STAT3 at a central node in the development, progression and maintenance of many human tumors signal transducer and activator of transcription 3 (for review see [69]). STAT3 proteins relay signals from activated cytokine and growth factor receptors in the plasma membrane to the nucleus, where they regulate gene transcription. STAT3 modulates the transcription of responsive genes that block apoptosis, favor cell proliferation and survival, and promote angiogenesis and metastasis. Disruption of STAT3 signaling leads to the inhibition of growth and apoptosis in tumor cell lines and can impair tumor growth in the mouse. Interestingly, recent studies have demonstrated that miR-202 is down-regulated in multiple types of cancer. These studies show that miR-202 is a novel tumor suppressor by targeting and inhibiting STAT3, as for example in non-small cell lung cancer [70]. Further investigation are now needed to validate the miR-202 and *stat3* interaction in medaka, and to better understand the role played by miR-202 and *stat3* on germ cell division and follicular growth in medaka ovary.

C*lockb* (clock circadian regulator b) is another noteworthy potential target of miR-202-5p. *Clock* genes are core components of the circadian peripheral clock machinery that plays critical role in the regulation of biological rhythms. *Clock* genes encode transcription factors of the basic helix-loop-helix (bHLH) family, which also contain intrinsic DNA binding histone acetyltransferase activity. Impaired fertility and fecundity have been observed in behaviorally arrhythmic clock mutant mice that are however still able to ovulate and produce fertilizable ova [71][72]. Further considerable evidences have been accumulated in mammals, linking the molecular circadian clock to ovarian physiology (for review see [73]). In granulosa cells, the clock machinery has been shown to regulate the timing and amplitude of several gene products associated with the ovulatory response to gonadotropins, including the LH receptor and prostaglandin synthase (COX2). *Clock* genes have also been shown to drive rhythms of enzymes critical for steroid hormone biosynthesis such as STAR, CYP11a, and aromatase (CYP19), which were down-regulated in the ovary of *mir-202* KO females. In the oocyte, it has been suggested that, rather than driving rhythms of gene expression, *clock* genes are part of the maternal program responsible for normal oocyte maturation and early embryonic development. Overall, existing data support a role for *clock* genes in the processes of follicular growth, steroid hormone synthesis and ovulation in mammals. Although the function of *clock* genes in fish ovary is still unknown, the role of miR-202 in the regulation of the early steps of follicular growth is consistent with a potential interaction with *clock* genes. It is clear that further studies on miR-202-5p and *clockb* interaction will be promising for the comprehension of the rhythmic follicle recruitment during the medaka reproductive cycles.

### MiR-202, a major player rather than a fine modulator

MiR-202 is a gonad-predominant miRNA in vertebrates, as documented here and in the literature. For protein-coding genes, a predominant expression in gonads is usually associated with a major role in reproduction, as illustrated by many maternal-effect genes [57]. So far, it was unknown if such a critical feature would also be observed for miRNAs that are often seen as fine modulators of gene regulatory networks rather than major regulators of biological processes [74]. Although several miRNAs are known to be key players of physiological processes [75], it is generally considered that the purview of miRNAs is more likely the maintenance of regulatory networks by fine-tuning gene expression, rather than the establishment of key regulatory networks for developmental decisions or core physiological processes. This concept is supported by the fact that KO of miRNAs are often associated with “modest” phenotypic effects at the level of the organism (*i*.*e*. low penetrance of the mutation), which are strongly exacerbated only under particular condition, as for example manipulations, stresses or disease conditions. In the present study, we observed a drastic reduction of the reproductive success for both male and female medaka lacking miR-202, including a reduced female fecundity and the production of poor quality eggs that cannot be fertilized. This phenotype was confirmed using two mutant lines bearing different INDELs obtained with the same guide RNA. Such high penetrance of the mutation is in contrast with the idea that miRNA KO generally induces “modest” phenotypes. It is however possible that miR-202 modulates a large network of targets, which would have a synergistic effect on key regulatory pathways for folliculogenesis. KO of *mir-202* gene would thus ultimately lead to subfertile or sterile females. Nevertheless, the phenotype observed in *mir-202* -/- fish is among the most severe observed after miRNA KO.

### A new look at oogenesis

Surprisingly, the transcriptomic analysis performed in juvenile females, during the first reproductive cycle and before the occurrence of the first spawning, did not result in the identification of a large number of differentially expressed. It should however be stressed that among the differentially expressed genes were many genes that are crucial for steroidegenesis and reproduction, such as *star* and two members of the WNT family (as discussed above). In addition to these usual suspects, the transcriptome analysis also shed light on other genes such as one gene of the KELCH family (*klhl23*) that is less studied but believed to play an important role in oogenesis based on existing data in other animal species [76]. Finally, the identification of genes that were previously not known to participate in oogenesis, such as *setd4*, *npr1b* and *srgap3*, could shed a new light on our understanding of this complex and coordinated biological process. This begs for further investigations that will greatly benefit from *mir-202 -/-* fishes as a novel biological model.

### Conclusion

In summary, our results show that miR-202 is a key miRNA involved in the regulation of follicular recruitment and growth. This provides the first functional evidence that miRNAs are necessary for the female reproductive success and in particular the regulation of female fecundity. Furthermore, the present study shed new light on the regulatory mechanisms that control the early steps of follicular development, which remain poorly understood to date. A further systematic *in vivo* functional analysis of other gonad-predominant miRNAs should greatly increase our knowledge on the overall role of miRNAs in oogenesis and female fecundity in fish.

## Materials and methods

### Ethics statement

All experimental procedures were conducted in strict accordance with the French and European regulations on animal welfare recommendations and were approved by the INRA LPGP Animal Care and Use Committee. For tissues/organs dissection, adult medaka fishes were euthanized by immersion in a lethal dose of tricaine at 30-50mg/L.

### Medaka breeding

Adult medaka (*Oryzias latipes*) from the CAB strain and adults of the *Tg(sox9b*::*EGFP)* medaka line were raised at 26°C. Juvenile fishes that had never reproduce were raised under a growing photoperiod regime (12 h light/ 12 h dark) until 3 months post-fertilization. From 3 months post-fertilization, reproductively active adult fishes were raised under a photoperiod regime (14 h light/10 h dark) that triggered reproduction.

### Establishment of the *mir-202* mutant medaka line

For the CRISPR/Cas9 knock-out analysis, the target genomic sequence was identified with the help of the ZiFiT online tool (http://zifit.partners.org/ZiFiT/) and using the medaka genome reference available on the Ensembl genome database (Ensembl gene: ENSORLG00000021212). A short sequence in the mature miR-202-3p was selected as followed: GG-(N)18-NN. Two inverse-complementary primers (Forward 5’-TAGGCATAAGGCATGGGAAAAT-3’ and Reverse 5’-AAACATTTTCCCATGCCTTATG-3’) were annealed and cloned into the pDR274 vector (Addgene plasmid 42250) in the BsaI cloning site. The modified pDR274 vector was digested with DraI and the miR-202 specific guide RNA (mir202-sgRNA) was transcribed using the T7 RNA polymerase (P207, Promega). For the Cas9-RNA *in vitro* synthesis, the pCS2-nCas9n vector (Addgene plasmid 47929) was linearized with NotI and capped RNA encoding the Cas9 was transcribed with the mMessage mMachine SP6 Kit (AM1340, Life Technologies) following manufacturer's instructions. Cas9 and sgRNA were purified using phenol/chloroform and precipitated by Ammonium acetate. *Cas9*-RNA (100 ng μl^-1^) and *mir-202*-sgRNA (10 ng μl^-1^) were co-injected into one-cell stage embryos. Injected embryos were raised to sexual maturity and 10 fishes were genotyped to identified founder fishes (F0). F0 fishes harboring the same INDEL mutation were selected and outcrossed with wild-type fishes to obtain F1 heterozygous. Such outcrosses were performed at each generation in order to maintain the line. Heterozygous fishes were crossed together to produce homozygous fishes for histological and molecular phenotyping analyses. Two distinct family lines harboring different INDEL mutations (-7+3 and -8) were used to analyze the reproductive phenotype.

### Genotyping

Genomic DNA was extracted from a small piece of the caudal fin sampled from anesthetized adult fishes. Samples were lysed in 75 ml of lysis buffer containing 1,25 M NaOH and 10 mM EDTA (pH 12) incubated at 90°C for 1 h and were neutralized with 75 mL of neutralization solution containing 2 M Tris-HCl (pH 5). To identify F0 founder fishes, genomic DNA around the expected mutation site was sequenced. For systematic genotyping of individuals of the established line, wild-type and mutant (INDEL -7+3) alleles were specifically detected by HIDI-PCR using specific reverse primers for each allele (S3 Table). The HIDI polymerase (Genaxxon bioscience, M3025.0250) was used with the following PCR conditions: 95°C for 2 min; and 40 cycles of 95°C for 20 sec, 57°C for 15 sec. and 72°C for 30 sec; and then 72°C for 7 min.

### Tissues collection

For tissues/organs dissection, adult medaka fishes were euthanized by immersion in a lethal dose of tricaine at 30–50 mg l^-1^. For qRT-PCR, microarray and small RNA-seq analyses, all tissues/organs and embryos were immediately frozen in liquid nitrogen and subsequently stored at −80°C until RNA extraction. For histological analyses, ovaries were collected from females, fixed overnight in 4% paraformaldehyde (PFA) at 4°C, dehydrated in 100% methanol and stored at -20°C.

### Total RNA extraction

Frozen tissues were lysed with Precellys Evolution Homogenizer (Ozyme, bertin technologies) in TRI Reagent (TR118, Euromedex) and total RNA was extracted using the “nucleospin RNA” kit (740955, Macherey Nagel).

### Quantitative RT-PCR

For qRT-PCR analysis, eleven different tissues/organs (brain, eyes, fins, gills, heart, intestine, kidney, liver, muscle, ovary and testis) were collected from wild-type and *mir-202* -/- fishes. Embryos were collected at different stages throughout embryonic development (st.0, st.2, st.5, st.11-12, st.17-18, st.25-26, st.29-30 and st.39), according to the developmental table described by Iwamatsu *et al*. [77]. For expression analysis of mature miRNA forms, 20 ng of total RNA were reverse-transcribed (RT) using the TaqMan advanced miRNA cDNA Synthesis Kit (A28007, Applied Biosystems). Twenty fmol μl^-1^ of an external calibrator cel-miR-39-3p (478293_miR, Life technologies) was added in the first step of the TaqMan qRT-PCR (polyA step) for 20ng of RNA. The cDNA was diluted (1:5) and universal primers (20x miR-Amp Primer Mix, 100029187, Applied Biosystems) were added in the last step of the RT reaction. The TaqMan qRT-PCR was performed using 5 μl of diluted cDNA, 1 μl TaqMan Advanced miRNA Assay solution (CCU001S, Special Product Custom designed advanced miRNA assay, Life technologies) and 10 μl Fast Advanced Master Mix (4444557, Applied Biosystems) in a total volume of 20 μl. Specific modified probes complementary to miR-202-5p and -3p were designed as followed: miR-202-3p 5’_FAM_-AGAGGCATAAGGCATGGGAAAA-3’_Quencher_ and miR-202-5p 5’_FAM_-TTCCTATGCATATACTTCTTTG-3’_Quencher_ (Life technologies). Quantitative RT-PCR was performed using the Step One Plus system (Applied Biosystems, USA) with the following conditions: 95°C for 20 sec; and 40 cycles of 95°C for 1 sec and 60°C for 20 sec. The relative expression of miRNA within a sample set was calculated from standard curve using Applied Biosystem StepOne V.2.0 software. All qRT-PCR were performed in duplicates. MiR-26a (hsa-miR-26a-5p, A25576, Thermofisher Scientific) or cel-miR-39-3p was used for normalization.

For mRNA and pri-mir-202 expression analysis, 2 μg of total RNA was reverse-transcribed using the Maxima First Strand cDNA Synthesis Kit (K1671, ThermoFisher Scientific). The cDNA was diluted (1:20). The SyberGreen qRT-PCR was performed using 4 μl of diluted cDNA, 5 μl of GoTaq PCR Master Mix 2x (A600A, Promega) and 100 nM of each primer (S4 Table), in a total volume of 10 μl. The qRT-PCR was performed using the Step One Plus system (Applied Biosystems, Foster City, USA) with the following conditions: 95°C for 2 min; and 40 cycles of 95°C for 15 sec and 60°C for 1 min. Standard curves were generated using five serial cDNA dilutions (from 1:2 to 1:32) of a pool of all samples. The relative abundance of target cDNA was calculated from standard curve using Applied Biosystem StepOne V.2.0 software. All qRT-PCR were performed in triplicates and the *rpl7* gene was used for normalization. For the validation of differentially expressed genes, 6 and 5 fish were used for wild-types and *mir-202* -/- groups, respectively.

### Microarray analysis

Gene expression profiling was conducted on ovaries from 3-month old juvenile females, using four biological replicates for both experimental groups (wild-type and *mir-202* -/-). We used an Agilent 8x60K microarray as previously described [78]. Samples were randomly distributed on the microarray for hybridization. The data were processed using the GeneSpring software (Agilent v.14.5) using gMedianSignal values. The gene expression data was scale normalized and log(2) transformed before the statistical analysis. Corresponding data were deposited in Gene Expression Omnibus (GEO) (https://www.ncbi.nlm.nih.gov/geo/) database under the reference GSE111388. Differences between experimental groups were analyzed with unpaired t-test after application of minimum two-fold change filter with the significance level of 5% (p < 0.05) by False Discovery Rate analysis (Benjamini-Hochberg correction).

### Small RNA-seq

MicroRNA sequencing was performed from the following medaka samples: brain, eye, fins, gills, heart, intestine, kidney, liver, muscle, ovary, testis, embryos at different developmental stages (st.0, st.2, st.5, st.15, st.27, st.31, st.35, st.39) and ovarian follicles enriched in different categories of follicles according to Iwamatsu *et al*. (st.I-II, st.III, st.IV, st.V) [1]. A single biological sample was used for each tissue/stage. For developmental stages, several embryos were pooled before RNA extraction. Twenty-five libraries were constructed using the NEXTflex small RNA ki v3 (Bioo Scientific). Starting from 1μg of total RNA, we ligated an adapter on the 3' end of the RNAs. A second adapter was ligated to the 5' end. Ligated RNAs were subjected to reverse transcription using M-MuLv transcriptase and a RT primer complementary to the 3' adapter. PCR amplification (16 cycles) was performed on the cDNA using a universal primer and a barcoded primer. Final size selection was performed on 3% gel cassette on a Pippin HT between 126pb and 169pb. Sequencing (single read 50 nucleotide) was performed using a HiSeq2500 (Illumina) with SBS (Sequence By Synthesis) technique. A total of over 271 million reads (sequences after Illumina Purity Filter) were obtained with a number of read per library ranging between 7.5 and 15. 2 millions. All reads were deposited into NCBI Sequence Read Archive under accession SRP151190. Reads were trimmed using cutadapt to remove the adapter sequence TGGAATTCTCGGGTGCAAGG and the random primers [79]. Reads were subsequently aligned onto the medaka genome (RefSeq assembly accession GCF_002234675.1) using miRDeep2 that accurately identifies known and novel microRNA genes in seven animal clades [80]. No mismatch was allowed, and reads shorter than 18 nucleotides were discarded. The number of miR-202-5p and miR-202-3p were subsequently counted in the different samples and normalized using RPM (Reads Per Million).

### MiR-202-5p RNA folding and target prediction

The sequence of miR-202-5p was used for *in silico* prediction of the hairpin secondary structure, based on minimum free energy structures and base pair probabilities, using the RNAfold web server with default settings (http://rna.tbi.univie.ac.at/cgi-bin/RNAWebSuite/RNAfold.cgi) [81]. MiR-202-5p targets were predicted with miRanda (3.3a version) [82] and TargetScan (5.0 version) [83] and using medaka mRNA sequence available in Ensembl (v92). For miRanda, default parameters (score 140, energy 0). In order to limit the number of false-positive predictions, only targets that were predicted by both miRanda and TargetScan and expressed in the medaka ovary were considered to be relevant.

### Fluorescent *in situ* hybridization and immunostaining

For fluorescent *in situ* hybridization (FISH), fixed ovaries were embedded in paraffin and sections (9 μm thickness) were performed with a microtome (HM355, microm). Alternatively, fixed ovaries were embedded in OCT and frozen sections (9 μm thickness) were performed with a cryostat (LEICA CM3050S). An anti-sense Locked Nucleic Acid (LNA) oligonucleotide was designed and produced by Exiqon A/S to detect the mature miR-202-3p form. Since medaka and human miR-202-5p sequences are identical, we used the hsa-miR-202-5p miRCURY LNA miRNA detection probe to detect the mature medaka miR-202-5p form. A LNA Scramble-miR probe (5’-GTGTAACACGTCTATACGCCCA-3’) was used as a negative control. All LNA probes were double-DIG labeled at both 5’ and 3’ ends. FISH was performed using the microRNA ISH Buffer Set (FFPE) Hybridization Buffer (ref. 90000, Exiqon), following the manufacturer's instructions with some modifications. Permeabilisation was performed for 7 min at room temperature using of Proteinase-K (10 mg/ml, P2308 Sigma). LNA probes were used at 20 nM at 53°C (30°C below the RNA Tm) for 2 h. Samples were then incubated overnight at 4°C with a rabbit anti-DIG HRP-conjugate antibody (1:500, Roche). For the GFP detection, a chicken anti-GFP (1:500, ref. ab13970, Abcam) was added at this step. The anti-GFP was first detected with a goat anti-chicken AlexaFluor488-conjugate antibody (1:500, ref. A11039, Life Technologies) for 1 h at room temperature. Then, the anti-DIG-HRP antibody was detected with the TSA-Cy5 substrate (1:50, TSA PLUS Cy5 kit, NEL 745001KT, Perkin Elmer) for 15 min at room temperature. All pictures were taken under SP8 confocal microscope.

### Nuclear staining and image analysis

Fixed ovaries were embedded in paraffin and sections (7 μm thickness) were performed with a microtome (HM355, microm). Only median sections of ovaries were kept for further analysis. Nuclei were stained on median sections with DAPI (0,1 μg ml^-1^) at room temperature for 15 minutes in the dark. Sections were washed 1 h in PBS at room temperature. Images of whole sections were acquired with a nanozoomer (HAMAMATSU). For quantitative image analyses, individual oocyte's area was measured using an image analysis software (Visilog 7.2 for Windows). Based on DAPI staining intensity, the nucleus were segmented then the inner part surround by the nucleus and corresponding to oocytes were individually measured. Pictures were taken under a Nikon AZ100 microscope and DS-Ri1 digital camera.

## Supporting information

S1 Fig Expression profiles of miR-202-5p and miR-202-3p in ovarian follicles obtained by small RNA-Seq. (TIF)

S2 Fig **Comparison of the expression pattern of miR-202-5p and miR-202-3p obtained by ISH using either frozen sections (A-C) or paraffin sections (D-G) of ovaries from adult females.** MiR-202-5p, miR-202-3p and miR-187-3p LNA probes, as well as a control scramble LNA probe were used. With miR-202-5p and miR-187-3p probes, we obtained an expression pattern restricted to granulosa cells (B and E, arrows) or oocytes (G, stars), respectively. No signals were obtained with miR-202-3p and scramble control probes (A, D and C, F).
(TIF)

S3 Fig FISH performed with a scramble control probe on paraffin sections of ovaries from juvenile and adult females. (TIF)

S1 Table Differentially expressed genes obtained through a microarray analysis. (PDF)

S2 Table Targets of miR-202-5p based on miRanda and TargetScan predictions. (PDF)

S3 Table Primers used for HIDI-PCR. (PDF)

S4 Table Primers used for qRT-PCR. (PDF)
