# Supplementary figures and images for: MiR-202 controls female fecundity by regulating medaka oogenesis
Source: PLoS Genet. 2018 Sep 10;14(9):e1007593. doi: 10.1371/journal.pgen.1007593 (PMC6147661; doi:10.1371/journal.pgen.1007593)

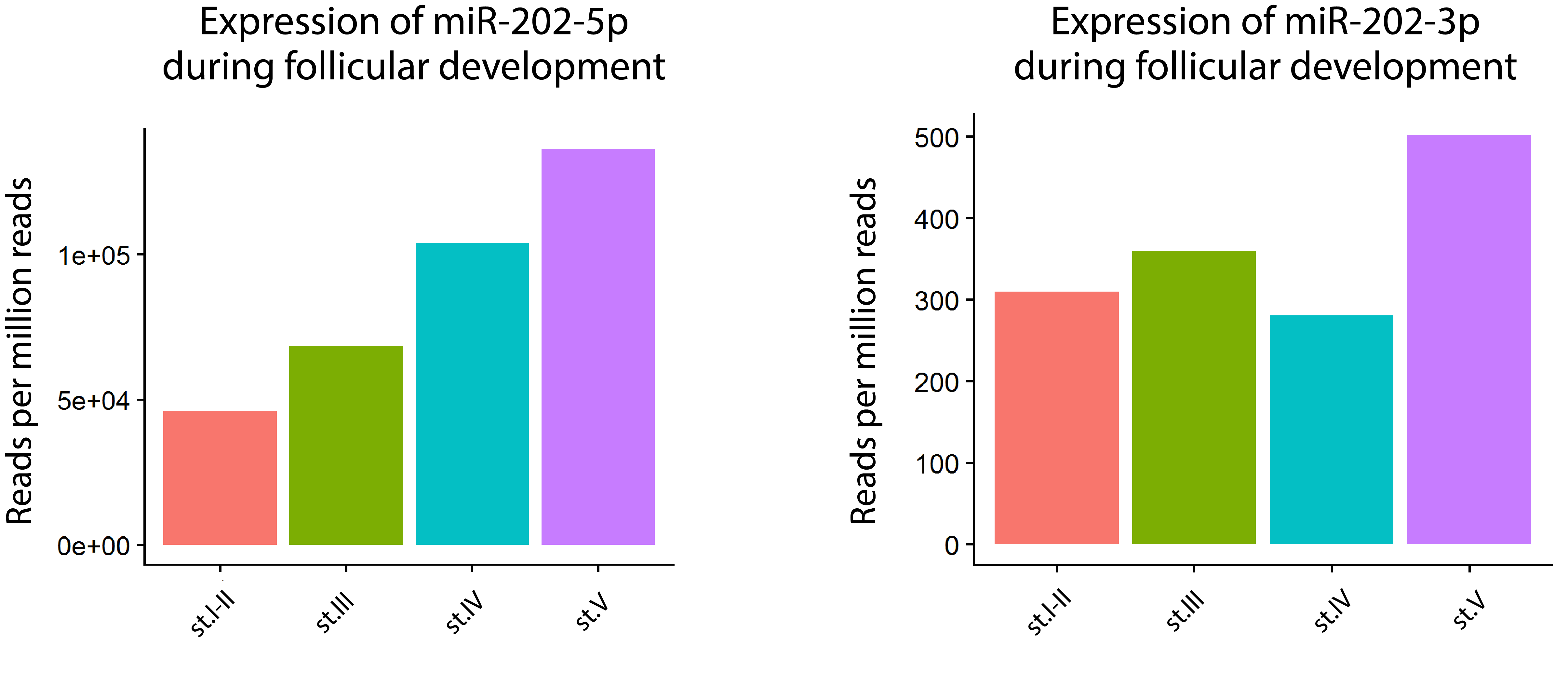

Supplement: S1 Fig — (TIF) [file pgen.1007593.s001.tif]

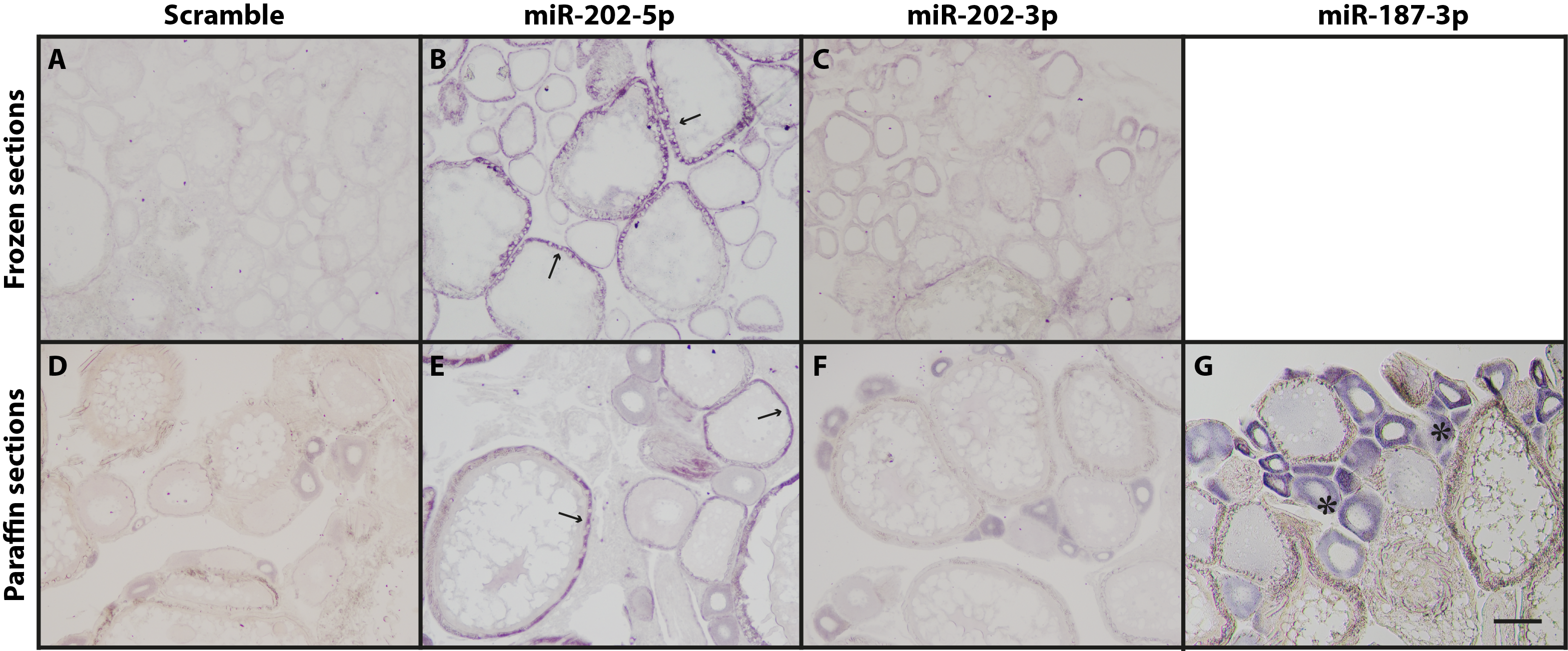

Supplement: S2 Fig — Comparison of the expression pattern of miR-202-5p and miR-202-3p obtained by ISH using either frozen sections (A-C) or paraffin sections (D-G) of ovaries from adult females. MiR-202-5p, miR-202-3p and miR-187-3p LNA probes, as well as a control scramble LNA probe were used. With miR-202-5p and miR-187-3p probes, we obtained an expression pattern restricted to granulosa cells (B and E, arrows) or oocytes (G, stars), respectively. No signals were obtained with miR-202-3p and scramble control probes (A, D and C, F). (TIF) [file pgen.1007593.s002.tif]

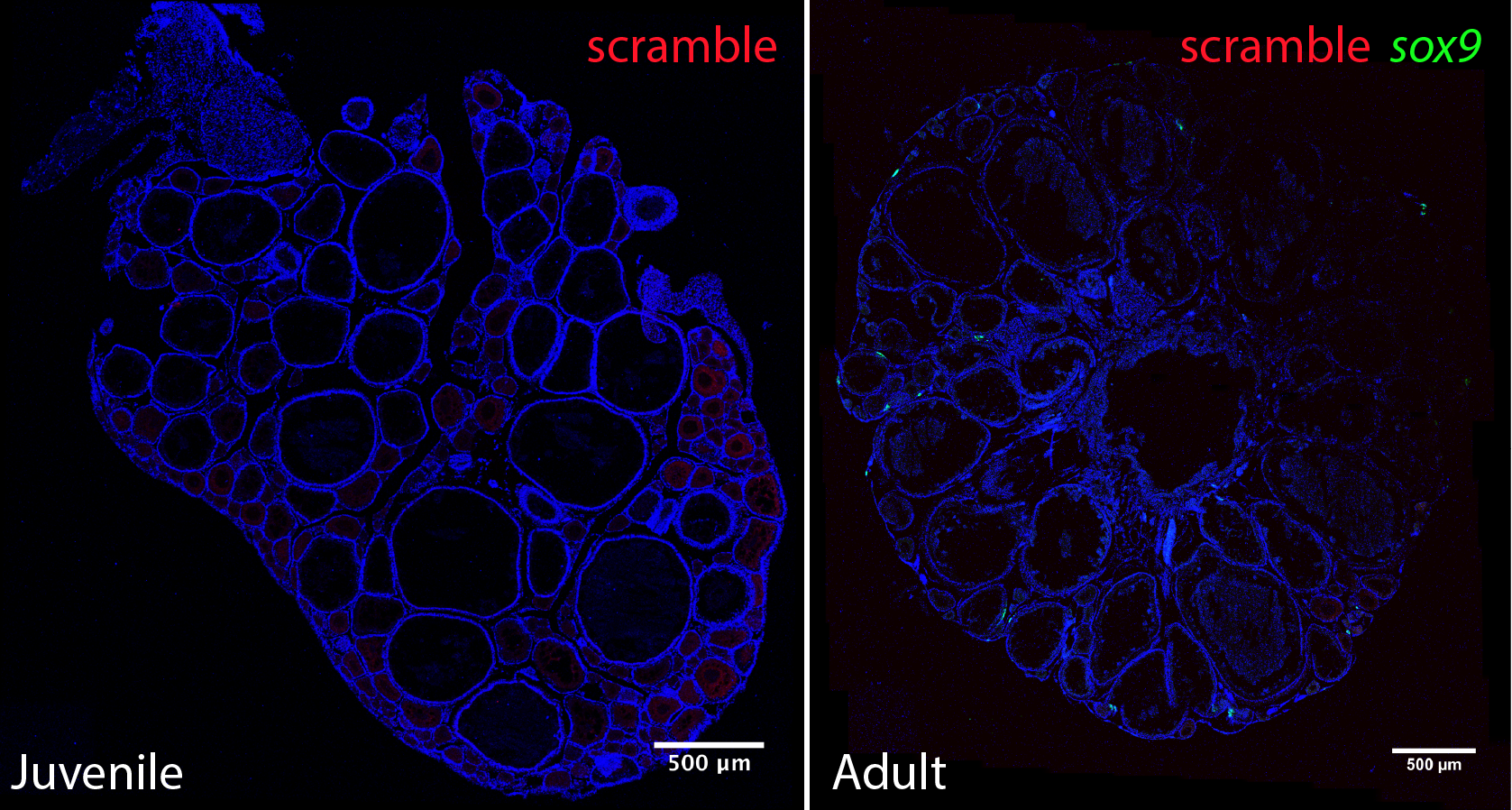

Supplement: S3 Fig — (TIF) [file pgen.1007593.s003.tif]
