# Supplementary material for: MiR-202 controls female fecundity by regulating medaka oogenesis
Source: PLoS Genet. 2018 Sep 10;14(9):e1007593. doi: 10.1371/journal.pgen.1007593 (PMC6147661; doi:10.1371/journal.pgen.1007593)
Supplement: S1 Table — (PDF) [file pgen.1007593.s004.pdf]

Notes : Created from Advanced Analysis operation: significance Analysis.
#Entitylist : Fold change >= 2.0
#Interpretation : Treatment (Non-averaged)
#Experiment: New Experiment
#corrected p-value cut-off:0.05
#Selected Test : T Test unpaired
#p-value computation: Asymptotic
#Multiple Testing Correction: Benjamini-Hochberg
#
Technology : Agilent.SingleColor.82421
Owner : gxuser
Created On : Fri Jun 02 16:04:15 CEST 2017

ProbeName	p (Corr)	p	Regulation	FC (abs)	FC
CUST_45763	0,028172938	4,43E-04	down	2,075612	-2,075612
CUST_25210	0,04061277	8,81E-04	down	2,1106827	-2,1106827
CUST_45868	0,005240591	1,03E-05	down	2,1391544	-2,1391544
CUST_35721	0,04369359	0,00134713	down	2,439573	-2,439573
CUST_6619_	0,045512788	0,00160936	down	2,5172336	-2,5172336
CUST_703_P	0,041526437	9,92E-04	down	2,548635	-2,548635
CUST_49074	0,045512788	0,00163998	down	3,103564	-3,103564
CUST_45596	0,017608102	1,06E-04	down	3,1225083	-3,1225083
CUST_4543_	0,03969736	8,31E-04	down	3,4979045	-3,4979045
CUST_963_P	7,91E-04	5,92E-07	up	7,241655	7,241655
CUST_41342	0,014321216	7,50E-05	up	6,0782	6,0782
CUST_30125	0,033430077	5,75E-04	up	3,5423074	3,5423074
CUST_23568	0,04369359	0,0013083	up	3,4702334	3,4702334
CUST_26528	0,04369359	0,00138775	up	3,114726	3,114726
CUST_19118	0,024941828	2,98E-04	up	3,0257053	3,0257053
CUST_42895	0,045512788	0,00149873	up	2,8776276	2,8776276
CUST_28351	0,04319733	0,00113082	up	2,5836635	2,5836635
CUST_39133	0,04319733	0,0010741	up	2,5670524	2,5670524
CUST_1200_	0,025215108	3,48E-04	up	2,527852	2,527852
CUST_39836	0,018476227	1,38E-04	up	2,5138113	2,5138113
CUST_25427	0,04882434	0,00189893	up	2,499248	2,499248
CUST_47226	0,04369359	0,00120936	up	2,4254782	2,4254782
CUST_30061	0,019330831	1,74E-04	up	2,4111335	2,4111335
CUST_17251	0,017608102	1,19E-04	up	2,3526726	2,3526726
CUST_34735	0,04863276	0,0018551	up	2,323327	2,323327
CUST_13295	0,03969736	8,05E-04	up	2,3219929	2,3219929
CUST_45850	0,045512788	0,00156972	up	2,2977266	2,2977266
CUST_17501	0,025215108	3,77E-04	up	2,2634015	2,2634015
CUST_22466	0,04369359	0,00128716	up	2,2490396	2,2490396
CUST_42391	0,006175743	2,05E-05	up	2,2363784	2,2363784
CUST_25528	0,04369359	0,00131231	up	2,1965227	2,1965227
CUST_4996_	0,019330831	1,72E-04	up	2,1837022	2,1837022
CUST_6829_	0,022902153	2,40E-04	up	2,1411421	2,1411421

CUST_40504	0,03559494	6,39E-04	up	2,1275694	2,1275694
CUST_142_P	0,04369359	0,00140525	up	2,1077418	2,1077418
CUST_38687	0,0414471	9,30E-04	up	2,099244	2,099244
CUST_15119	0,04319733	0,00111421	up	2,0905802	2,0905802
CUST_11749	0,03714719	6,95E-04	up	2,0854282	2,0854282
CUST_2258_	0,025215108	3,21E-04	up	2,0842583	2,0842583
CUST_37841	0,025215108	3,71E-04	up	2,074868	2,074868
CUST_30025	0,045512788	0,0016688	up	2,0195472	2,0195472
CUST_31271	0,012130885	5,44E-05	up	2,0030596	2,0030596

Log FC	WT 1++.txt:gProc	WT 7++.txt:gProc	WT 8++.txt:gProc	WT 12++.txt:gPrc	miR-202 KO 4--.txt:gProce	miR-202 KO 5--.txt:gProce
-1,0535368	13	20	13	13	32	32
-1,0777097	114	187	125	174	205	345
-1,0970407	99	123	90	126	197	230
-1,2866287	608	690	956	576	1174	1759
-1,3318391	57	84	119	71	213	206
-1,3497248	274	594	367	441	1124	1050
-1,6339259	12	23	16	22	82	41
-1,6427053	37	51	49	43	151	162
-1,8064909	70	120	92	119	214	264
2,8563194	2771	3494	3486	3501	445	412
2,6036441	45	69	55	88	6	13
1,8246894	40	30	53	32	11	10
1,7950327	28	35	30	49	16	8
1,6391053	23	25	20	33	5	8
1,5972714	20	31	34	25	7	10
1,5248799	23	23	14	27	6	11
1,3694181	92	100	92	151	27	45
1,3601127	88	182	132	165	40	42
1,337912	71	114	72	69	28	34
1,3298764	171	185	176	248	79	81
1,3214941	35	33	28	63	12	16
1,2782692	30	30	26	38	14	14
1,2697115	80	78	61	90	28	33
1,2343005	84	105	90	137	43	38
1,2161922	53	56	61	56	24	28
1,2153635	157	169	160	159	84	49
1,2002071	191	186	190	316	100	94
1,1784925	225	219	146	248	75	93
1,1693091	76	121	101	147	37	56
1,1611643	522	538	589	606	242	253
1,1352214	32	43	34	55	13	23
1,1267761	33	32	25	36	11	16
1,0983806	69	91	95	89	30	42

1,0892062	294	322	311	387	185	136
1,0756981	22	35	28	41	12	18
1,0698699	108	143	105	176	49	54
1,0639035	28	42	33	51	18	21
1,0603436	129	200	148	209	78	88
1,0595341	216	319	250	254	98	132
1,0530195	154	191	172	216	77	106
1,0140319	88	106	98	158	51	63
1,0022054	199	237	235	224	107	119

miR-202 KO	miR-202 KO					
17--.txt:gPro	20--.txt:gPro	1++.txt:gProc	7++.txt:gProc	8++.txt:gProc	12++.txt:gPr	4--.txt:gProce
20	31	4	4	4	4	5
284	346	7	7	7	7	8
206	234	7	7	7	7	8
1451	2058	9	9	10	9	10
148	187	6	6	7	6	8
623	1137	8	9	9	9	10
36	60	4	4	4	4	6
86	134	5	5	6	5	7
323	565	6	7	7	7	8
445	397	12	12	12	12	9
10	11	6	6	6	6	3
7	12	5	5	6	5	4
6	9	5	5	5	6	4
6	13	5	4	4	5	2
9	8	4	5	5	5	3
6	6	5	4	4	5	3
44	40	7	6	7	7	5
51	70	7	7	7	7	5
20	38	6	7	6	6	5
62	66	8	7	7	8	6
12	16	5	5	5	6	4
7	13	5	5	5	5	4
30	27	6	6	6	6	5
36	45	6	6	7	7	6
13	30	6	6	6	6	5
58	73	7	7	7	7	7
78	79	8	7	8	8	7
75	98	8	8	7	8	6
47	41	6	7	7	7	5
195	254	9	9	9	9	8
16	18	5	5	5	6	4
12	16	5	5	5	5	4
40	39	6	6	7	6	5

117	142	8	8	8	8	8
10	15	5	5	5	5	4
64	65	7	7	7	7	6
13	16	5	5	5	6	4
51	91	7	7	7	8	6
84	160	8	8	8	8	7
57	96	7	7	7	8	6
40	51	7	6	7	7	6
94	98	8	8	8	8	7

5--.txt:gProc	17--.txt:gPro	20--.txt:gPro	Description
5	5	5	
8	8	8	zgc:123321 [Source:ZFIN;Acc:ZDB-GENE-051023-1]
8	8	8	
11	11	11	SLIT-ROBO Rho GTPase activating protein 3 [Source:ZFIN;A
8	7	7	si:dkey-49n23.1 [Source:ZFIN;Acc:ZDB-GENE-050208-762]
10	10	10	
5	5	6	natriuretic peptide receptor 1b [Source:ZFIN;Acc:ZDB-GEN
7	7	7	basonuclin 1 [Source:HGNC Symbol;Acc:HGNC:1081]
8	9	9	Oryzias latipes myosin heavy chain (mmyhemb1), mRNA. [S
9	9	9	SET domain containing 4 [Source:ZFIN;Acc:ZDB-GENE-0508
4	4	3	kelch-like family member 23 [Source:ZFIN;Acc:ZDB-GENE-0
3	3	3	zinc finger C3H1-type containing [Source:HGNC Symbol;Acc
3	3	3	
3	3	4	
3	3	3	wingless-type MMTV integration site family, member 2Bb
3	3	3	small integral membrane protein 8 [Source:ZFIN;Acc:ZDB-G
5	6	5	Oryzias latipes wingless-type MMTV integration site family
5	6	6	si:dkey-194e6.1 [Source:ZFIN;Acc:ZDB-GENE-100922-43]
5	5	5	inositol polyphosphate-5-phosphatase A [Source:HGNC Syr
6	6	6	
4	4	4	hydroxysteroid (17-beta) dehydrogenase 7 [Source:ZFIN;Ac
4	3	4	zgc:172271 [Source:ZFIN;Acc:ZDB-GENE-080204-110]
5	5	5	solute carrier family 23 (ascorbic acid transporter), membe
5	5	5	heparan sulfate (glucosamine) 3-O-sulfotransferase 3B1a [S
5	4	5	inositol 1,4,5-trisphosphate receptor, type 1a [Source:ZFIN
6	6	6	si:dkeyp-69c1.7 [Source:ZFIN;Acc:ZDB-GENE-091204-69]
7	7	6	Oryzias latipes cytochrome P-450 17alpha-hydroxylase/C17
7	6	7	myosin, light chain 6, alkali, smooth muscle and non-muscl
6	6	5	UDP-Gal:betaGlcNAc beta 1,4- galactosyltransferase, polyp
8	8	8	ADAM metallopeptidase domain 23a [Source:ZFIN;Acc:ZDB
4	4	4	discoidin domain receptor tyrosine kinase 2b [Source:ZFIN;
4	4	4	inhibin, alpha [Source:ZFIN;Acc:ZDB-GENE-060503-554]
5	6	5	tumor necrosis factor, alpha-induced protein 8-like 3 [Sour

7	7	7	fibroblast activation protein, alpha [Source:ZFIN;Acc:ZDB-G
4	4	4	
6	6	6	
4	4	4	
6	6	6	solute carrier family 43 (amino acid system L transporter),
7	7	7	
7	6	6	myelin protein zero like 2 [Source:HGNC Symbol;Acc:HGNC
6	6	6	Oryzias latipes steroidogenic acute regulatory protein (star
7	7	7	solute carrier family 20, member 1a [Source:ZFIN;Acc:ZDB-

Fold-change	Gene ID	Gene Symbol	Transcript ID
**2,1**	ENSORLG00000019183		ENSORLT00000023904.1
**2,1**	ENSORLG00000010730	zgc:123321	ENSORLT00000013454.1
**2,1**	ENSORLG00000019238		ENSORLT00000023970.1
**2,4**	ENSORLG00000000702	srgap3	ENSORLT00000000866.2
**2,5**	ENSORLG00000002866	si:dkey-49n23.1	ENSORLT00000003586.1
**2,5**	ENSORLG00000000326		ENSORLT00000000396.1
**3,1**	ENSORLG00000020832	npr1b	ENSORLT00000025799.1
**3,1**	ENSORLG00000019081	BNC1	ENSORLT00000023795.1
**3,5**	ENSORLG00000001985	mmyhemb1	ENSORLT00000002483.1
**7,2**	ENSORLG00000000434	setd4	ENSORLT00000000535.1
**6,1**	ENSORLG00000017198	klhl23	ENSORLT00000021517.1
**3,5**	ENSORLG00000000155	ZFC3H1	ENSORLT00000000193.3
**3,5**	ENSORLG00000010020		ENSORLT00000012572.1
**3,1**	ENSORLG00000011290		ENSORLT00000014146.1
**3,0**	ENSORLG00000008125	wnt2bb	ENSORLT00000010199.1
**2,9**	ENSORLG00000017811	smim8	ENSORLT00000022290.1
**2,6**	ENSORLG00000012025	wnt4	ENSORLT00000015061.1
**2,6**	ENSORLG00000016335	si:dkey-194e6.1	ENSORLT00000020453.1
**2,5**	ENSORLG00000000540	INPP5A	ENSORLT00000000666.1
**2,5**	ENSORLG00000016620		ENSORLT00000020802.1
**2,5**	ENSORLG00000010814	hsd17b7 (1 of many)	ENSORLT00000013564.1
**2,4**	ENSORLG00000019946	zgc:172271	ENSORLT00000024771.1
**2,4**	ENSORLG00000012701	slc23a1	ENSORLT00000015919.1
**2,4**	ENSORLG00000007353	hs3st3b1a	ENSORLT00000009221.1
**2,3**	ENSORLG00000014557	itpr1a	ENSORLT00000018259.1
**2,3**	ENSORLG00000005686	si:dkeyp-69c1.7	ENSORLT00000007149.1
**2,3**	ENSORLG00000019226	cyp17	ENSORLT00000023960.1
**2,3**	ENSORLG00000007460	myl6	ENSORLT00000009354.1
**2,2**	ENSORLG00000009547	b4galt5	ENSORLT00000011972.1
**2,2**	ENSORLG00000017616	adam23a	ENSORLT00000022043.1
**2,2**	ENSORLG00000010857	ddr2b	ENSORLT00000013618.1
**2,2**	ENSORLG00000002184	inha	ENSORLT00000002714.1
**2,1**	ENSORLG00000002956	tnfaip8l3	ENSORLT00000003692.1

**2,1**	ENSORLG00000016880	fap	ENSORLT00000021121.1
**2,1**	ENSORLG00000000067		ENSORLT00000000080.1
**2,1**	ENSORLG00000016176		ENSORLT00000020238.1
**2,1**	ENSORLG00000006452		ENSORLT00000008100.1
**2,1**	ENSORLG00000005019	slc43a2b	ENSORLT00000006327.1
**2,1**	ENSORLG00000001022		ENSORLT00000001258.1
**2,1**	ENSORLG00000015826	MPZL2 (1 of many)	ENSORLT00000019822.1
**2,0**	ENSORLG00000012688	star	ENSORLT00000015903.1
**2,0**	ENSORLG00000013173	slc20a1a	ENSORLT00000016515.1
