# Supplementary material for: MiR-202 controls female fecundity by regulating medaka oogenesis
Source: PLoS Genet. 2018 Sep 10;14(9):e1007593. doi: 10.1371/journal.pgen.1007593 (PMC6147661; doi:10.1371/journal.pgen.1007593)
Supplement: S2 Table — (PDF) [file pgen.1007593.s005.pdf]

ARNm	miRanda	TargetScan	Gene
ENSORLT0000	5UTR	UTR5	ENSORLG00000006109
ENSORLT0000	5UTR	UTR5	ENSORLG00000013749
ENSORLT0000	5UTR	UTR5	ENSORLG00000018359
ENSORLT0000	5UTR	UTR5	ENSORLG00000015923
ENSORLT0000	5UTR	UTR5	ENSORLG00000010897
ENSORLT0000	5UTR	UTR5	ENSORLG00000009609
ENSORLT0000	5UTR	UTR5	ENSORLG00000009798
ENSORLT0000	5UTR	UTR5	ENSORLG00000002648
ENSORLT0000	5UTR	UTR5	ENSORLG00000003605
ENSORLT0000	5UTR	UTR5	ENSORLG00000015980
ENSORLT0000	5UTR	UTR5	ENSORLG00000015246
ENSORLT0000	5UTR	UTR5	ENSORLG00000015246
ENSORLT0000	5UTR	UTR5	ENSORLG00000006559
ENSORLT0000	5UTR	UTR5	ENSORLG00000015878
ENSORLT0000	5UTR	UTR5	ENSORLG00000004305
ENSORLT0000	3UTR	UTR3	ENSORLG00000000991
ENSORLT0000	3UTR	UTR3	ENSORLG00000010803
ENSORLT0000	3UTR	UTR3	ENSORLG00000003787
ENSORLT0000	3UTR	UTR3	ENSORLG00000019070
ENSORLT0000	3UTR	UTR3	ENSORLG00000004061
ENSORLT0000	3UTR	UTR3	ENSORLG00000020425
ENSORLT0000	3UTR	UTR3	ENSORLG00000018349
ENSORLT0000	3UTR	UTR3	ENSORLG00000014188
ENSORLT0000	3UTR	UTR3	ENSORLG00000011661
ENSORLT0000	3UTR	UTR3	ENSORLG00000002452
ENSORLT0000	3UTR	UTR3	ENSORLG00000014598
ENSORLT0000	3UTR	UTR3	ENSORLG00000013621
ENSORLT0000	3UTR	UTR3	ENSORLG00000004495
ENSORLT0000	3UTR	UTR3	ENSORLG00000019685
ENSORLT0000	3UTR	UTR3	ENSORLG00000017948
ENSORLT0000	3UTR	UTR3	ENSORLG00000013973
ENSORLT0000	3UTR	UTR3	ENSORLG00000016249
ENSORLT0000	3UTR	UTR3	ENSORLG00000020656
ENSORLT0000	CDS	CDS	ENSORLG00000002627
ENSORLT0000	CDS	CDS	ENSORLG00000006241
ENSORLT0000	CDS	CDS	ENSORLG00000008019
ENSORLT0000	CDS	CDS	ENSORLG00000014801
ENSORLT0000	CDS	CDS	ENSORLG00000006928
ENSORLT0000	CDS	CDS	ENSORLG00000007874
ENSORLT0000	CDS	CDS	ENSORLG00000014056
ENSORLT0000	CDS	CDS	ENSORLG00000005151
ENSORLT0000	CDS	CDS	ENSORLG00000010050
ENSORLT0000	CDS	CDS	ENSORLG00000015608
ENSORLT0000	CDS	CDS	ENSORLG00000005341
ENSORLT0000	CDS	CDS	ENSORLG00000000259
ENSORLT0000	CDS	CDS	ENSORLG00000004730
ENSORLT0000	CDS	CDS	ENSORLG00000014323
ENSORLT0000	CDS	CDS	ENSORLG00000000526
ENSORLT0000	CDS	CDS	ENSORLG00000003898
ENSORLT0000	CDS	CDS	ENSORLG00000011395
ENSORLT0000	CDS	CDS	ENSORLG00000014142

ENSORLT0000	CDS	CDS	ENSORLG00000015669
ENSORLT0000	CDS	CDS	ENSORLG00000016882
ENSORLT0000	CDS	CDS	ENSORLG00000009280
ENSORLT0000	CDS	CDS	ENSORLG00000004843
ENSORLT0000	CDS	CDS	ENSORLG00000014910
ENSORLT0000	CDS	CDS	ENSORLG00000008476
ENSORLT0000	CDS	CDS	ENSORLG00000014338
ENSORLT0000	CDS	CDS	ENSORLG00000003334
ENSORLT0000	CDS	CDS	ENSORLG00000012680
ENSORLT0000	CDS	CDS	ENSORLG00000005956
ENSORLT0000	CDS	CDS	ENSORLG00000017690
ENSORLT0000	CDS	CDS	ENSORLG00000015634
ENSORLT0000	CDS	CDS	ENSORLG00000004833
ENSORLT0000	CDS	CDS	ENSORLG00000016108
ENSORLT0000	CDS	CDS	ENSORLG00000011497
ENSORLT0000	CDS	CDS	ENSORLG00000005779
ENSORLT0000	CDS	CDS	ENSORLG00000019403
ENSORLT0000	CDS	CDS	ENSORLG00000019403
ENSORLT0000	CDS	CDS	ENSORLG00000006881
ENSORLT0000	CDS	CDS	ENSORLG00000011737
ENSORLT0000	CDS	CDS	ENSORLG00000003272
ENSORLT0000	CDS	CDS	ENSORLG00000017319
ENSORLT0000	CDS	CDS	ENSORLG00000003182
ENSORLT0000	CDS	CDS	ENSORLG00000002130
ENSORLT0000	CDS	CDS	ENSORLG00000010741
ENSORLT0000	CDS	CDS	ENSORLG00000007525
ENSORLT0000	CDS	CDS	ENSORLG00000010171
ENSORLT0000	CDS	CDS	ENSORLG00000008026
ENSORLT0000	CDS	CDS	ENSORLG00000009005
ENSORLT0000	CDS	CDS	ENSORLG00000002336
ENSORLT0000	CDS	CDS	ENSORLG00000014368
ENSORLT0000	CDS	CDS	ENSORLG00000005217
ENSORLT0000	CDS	CDS	ENSORLG00000000676
ENSORLT0000	CDS	CDS	ENSORLG00000014324
ENSORLT0000	CDS	CDS	ENSORLG00000020389
ENSORLT0000	CDS	CDS	ENSORLG00000016842
ENSORLT0000	CDS	CDS	ENSORLG00000008417
ENSORLT0000	CDS	CDS	ENSORLG00000016381
ENSORLT0000	CDS	CDS	ENSORLG00000014520
ENSORLT0000	CDS	CDS	ENSORLG00000006870
ENSORLT0000	CDS	CDS	ENSORLG00000001045
ENSORLT0000	CDS	CDS	ENSORLG00000002132
ENSORLT0000	CDS	CDS	ENSORLG00000004556
ENSORLT0000	CDS	CDS	ENSORLG00000007776
ENSORLT0000	CDS	CDS	ENSORLG00000009222
ENSORLT0000	CDS	CDS	ENSORLG00000016736
ENSORLT0000	CDS	CDS	ENSORLG00000007244
ENSORLT0000	CDS	CDS	ENSORLG00000011554
ENSORLT0000	CDS	CDS	ENSORLG00000011500
ENSORLT0000	CDS	CDS	ENSORLG00000002443
ENSORLT0000	CDS	CDS	ENSORLG00000011353
ENSORLT0000	CDS	CDS	ENSORLG00000013827

ENSORLT0000	CDS	CDS	ENSORLG00000017116
ENSORLT0000	CDS	CDS	ENSORLG00000008279
ENSORLT0000	CDS	CDS	ENSORLG00000001843
ENSORLT0000	CDS	CDS	ENSORLG00000004636
ENSORLT0000	CDS	CDS	ENSORLG00000002809
ENSORLT0000	CDS	CDS	ENSORLG00000016946
ENSORLT0000	CDS	CDS	ENSORLG00000017184
ENSORLT0000	CDS	CDS	ENSORLG00000008773
ENSORLT0000	CDS	CDS	ENSORLG00000003164
ENSORLT0000	CDS	CDS	ENSORLG00000006341
ENSORLT0000	CDS	CDS	ENSORLG00000011579
ENSORLT0000	CDS	CDS	ENSORLG00000013616
ENSORLT0000	CDS	CDS	ENSORLG00000015459
ENSORLT0000	CDS	CDS	ENSORLG00000006803
ENSORLT0000	CDS	CDS	ENSORLG00000015683
ENSORLT0000	CDS	CDS	ENSORLG00000000335
ENSORLT0000	CDS	CDS	ENSORLG00000003885
ENSORLT0000	CDS	CDS	ENSORLG00000006721
ENSORLT0000	CDS	CDS	ENSORLG00000015180
ENSORLT0000	CDS	CDS	ENSORLG00000004722
ENSORLT0000	CDS	CDS	ENSORLG00000004069
ENSORLT0000	CDS	CDS	ENSORLG00000005675
ENSORLT0000	CDS	CDS	ENSORLG00000009655
ENSORLT0000	CDS	CDS	ENSORLG00000014005
ENSORLT0000	CDS	CDS	ENSORLG00000014107
ENSORLT0000	CDS	CDS	ENSORLG00000007304
ENSORLT0000	CDS	CDS	ENSORLG00000006678
ENSORLT0000	CDS	CDS	ENSORLG00000012273
ENSORLT0000	CDS	CDS	ENSORLG00000014284
ENSORLT0000	CDS	CDS	ENSORLG00000018414
ENSORLT0000	CDS	CDS	ENSORLG00000009149
ENSORLT0000	CDS	CDS	ENSORLG00000004339
ENSORLT0000	CDS	CDS	ENSORLG00000004339
ENSORLT0000	CDS	CDS	ENSORLG00000010253
ENSORLT0000	CDS	CDS	ENSORLG00000014868
ENSORLT0000	CDS	CDS	ENSORLG00000002482
ENSORLT0000	CDS	CDS	ENSORLG00000016893
ENSORLT0000	CDS	CDS	ENSORLG00000005221
ENSORLT0000	CDS	CDS	ENSORLG00000011486
ENSORLT0000	CDS	CDS	ENSORLG00000006343
ENSORLT0000	CDS	CDS	ENSORLG00000008128
ENSORLT0000	CDS	CDS	ENSORLG00000000754
ENSORLT0000	CDS	CDS	ENSORLG00000006927
ENSORLT0000	CDS	CDS	ENSORLG00000006927
ENSORLT0000	CDS	CDS	ENSORLG00000009090
ENSORLT0000	CDS	CDS	ENSORLG00000011733
ENSORLT0000	CDS	CDS	ENSORLG00000015155
ENSORLT0000	CDS	CDS	ENSORLG00000006476
ENSORLT0000	CDS	CDS	ENSORLG00000005342
ENSORLT0000	CDS	CDS	ENSORLG00000018938
ENSORLT0000	CDS	CDS	ENSORLG00000012294
ENSORLT0000	CDS	CDS	ENSORLG00000011414

ENSORLT0000	CDS	CDS	ENSORLG00000014038
ENSORLT0000	CDS	CDS	ENSORLG00000000780
ENSORLT0000	CDS	CDS	ENSORLG00000017439
ENSORLT0000	CDS	CDS	ENSORLG00000010789
ENSORLT0000	CDS	CDS	ENSORLG00000002854
ENSORLT0000	CDS	CDS	ENSORLG00000016135
ENSORLT0000	CDS	CDS	ENSORLG00000007406
ENSORLT0000	CDS	CDS	ENSORLG00000007406
ENSORLT0000	CDS	CDS	ENSORLG00000017635
ENSORLT0000	CDS	CDS	ENSORLG00000006135
ENSORLT0000	CDS	CDS	ENSORLG00000006135
ENSORLT0000	CDS	CDS	ENSORLG00000005502
ENSORLT0000	CDS	CDS	ENSORLG00000010348
ENSORLT0000	CDS	CDS	ENSORLG00000011194
ENSORLT0000	CDS	CDS	ENSORLG00000012529
ENSORLT0000	CDS	CDS	ENSORLG00000014576
ENSORLT0000	CDS	CDS	ENSORLG00000016092
ENSORLT0000	CDS	CDS	ENSORLG00000005452
ENSORLT0000	CDS	CDS	ENSORLG00000002755
ENSORLT0000	CDS	CDS	ENSORLG00000002755
ENSORLT0000	CDS	CDS	ENSORLG00000015825
ENSORLT0000	CDS	CDS	ENSORLG00000014947
ENSORLT0000	CDS	CDS	ENSORLG00000004718
ENSORLT0000	CDS	CDS	ENSORLG00000004718
ENSORLT0000	CDS	CDS	ENSORLG00000008891
ENSORLT0000	CDS	CDS	ENSORLG00000017098
ENSORLT0000	CDS	CDS	ENSORLG00000003353
ENSORLT0000	CDS	CDS	ENSORLG00000010271
ENSORLT0000	CDS	CDS	ENSORLG00000000583
ENSORLT0000	CDS	CDS	ENSORLG00000005243
ENSORLT0000	CDS	CDS	ENSORLG00000002814
ENSORLT0000	CDS	CDS	ENSORLG00000007572
ENSORLT0000	3UTR	CDS	ENSORLG00000019070
ENSORLT0000	CDS	CDS	ENSORLG00000010438
ENSORLT0000	CDS	CDS	ENSORLG00000005124
ENSORLT0000	CDS	CDS	ENSORLG00000003115
ENSORLT0000	CDS	CDS	ENSORLG00000008271
ENSORLT0000	CDS	CDS	ENSORLG00000011316
ENSORLT0000	CDS	CDS	ENSORLG00000008651
ENSORLT0000	CDS	CDS	ENSORLG00000014048
ENSORLT0000	CDS	CDS	ENSORLG00000013698
ENSORLT0000	CDS	CDS	ENSORLG00000016356
ENSORLT0000	CDS	CDS	ENSORLG00000016356
ENSORLT0000	CDS	CDS	ENSORLG00000007854
ENSORLT0000	CDS	CDS	ENSORLG00000017625
ENSORLT0000	CDS	CDS	ENSORLG00000003838
ENSORLT0000	CDS	CDS	ENSORLG00000006292
ENSORLT0000	CDS	CDS	ENSORLG00000017589
ENSORLT0000	CDS	CDS	ENSORLG00000001466
ENSORLT0000	CDS	CDS	ENSORLG00000013388
ENSORLT0000	CDS	CDS	ENSORLG00000005535
ENSORLT0000	CDS	CDS	ENSORLG00000006402

ENSORLT0000	CDS	CDS	ENSORLG00000016819
ENSORLT0000	CDS	CDS	ENSORLG00000014787
ENSORLT0000	CDS	CDS	ENSORLG00000011520
ENSORLT0000	CDS	CDS	ENSORLG00000015647
ENSORLT0000	CDS	CDS	ENSORLG00000005715
ENSORLT0000	CDS	CDS	ENSORLG00000013588
ENSORLT0000	CDS	CDS	ENSORLG00000006597
ENSORLT0000	CDS	CDS	ENSORLG00000013704
ENSORLT0000	CDS	CDS	ENSORLG00000012143
ENSORLT0000	CDS	CDS	ENSORLG00000005227
ENSORLT0000	CDS	CDS	ENSORLG00000000111
ENSORLT0000	CDS	CDS	ENSORLG00000009216
ENSORLT0000	CDS	CDS	ENSORLG00000004787
ENSORLT0000	CDS	CDS	ENSORLG00000006124
ENSORLT0000	CDS	CDS	ENSORLG00000016677
ENSORLT0000	CDS	CDS	ENSORLG00000003854
ENSORLT0000	CDS	CDS	ENSORLG00000006500
ENSORLT0000	CDS	CDS	ENSORLG00000006455
ENSORLT0000	CDS	CDS	ENSORLG00000006004
ENSORLT0000	CDS	CDS	ENSORLG00000013569
ENSORLT0000	CDS	CDS	ENSORLG00000019526
ENSORLT0000	CDS	CDS	ENSORLG00000012928
ENSORLT0000	CDS	CDS	ENSORLG00000020394
ENSORLT0000	CDS	CDS	ENSORLG00000014294
ENSORLT0000	CDS	CDS	ENSORLG00000000479
ENSORLT0000	CDS	CDS	ENSORLG00000011314
ENSORLT0000	CDS	CDS	ENSORLG00000020132
ENSORLT0000	CDS	CDS	ENSORLG00000008354
ENSORLT0000	CDS	CDS	ENSORLG00000012985
ENSORLT0000	CDS	CDS	ENSORLG00000002730
ENSORLT0000	CDS	CDS	ENSORLG00000002730
ENSORLT0000	CDS	CDS	ENSORLG00000018531
ENSORLT0000	CDS	CDS	ENSORLG00000013530
ENSORLT0000	CDS	CDS	ENSORLG00000008541
ENSORLT0000	CDS	CDS	ENSORLG00000002944
ENSORLT0000	CDS	CDS	ENSORLG00000020323
ENSORLT0000	CDS	CDS	ENSORLG00000004102
ENSORLT0000	CDS	CDS	ENSORLG00000010031
ENSORLT0000	CDS	CDS	ENSORLG00000007264
ENSORLT0000	CDS	CDS	ENSORLG00000008478
ENSORLT0000	CDS	CDS	ENSORLG00000008073
ENSORLT0000	CDS	CDS	ENSORLG00000012811
ENSORLT0000	CDS	CDS	ENSORLG00000000794
ENSORLT0000	CDS	CDS	ENSORLG00000001076
ENSORLT0000	CDS	CDS	ENSORLG00000006926
ENSORLT0000	CDS	CDS	ENSORLG00000019976
ENSORLT0000	CDS	CDS	ENSORLG00000014361
ENSORLT0000	CDS	CDS	ENSORLG00000018260
ENSORLT0000	CDS	CDS	ENSORLG00000013529
ENSORLT0000	CDS	CDS	ENSORLG00000013744
ENSORLT0000	CDS	CDS	ENSORLG00000007549
ENSORLT0000	CDS	CDS	ENSORLG00000006899

ENSORLT0000	CDS	CDS	ENSORLG00000018222
ENSORLT0000	CDS	CDS	ENSORLG00000019677
ENSORLT0000	CDS	CDS	ENSORLG00000011141
ENSORLT0000	CDS	CDS	ENSORLG00000007583
ENSORLT0000	CDS	CDS	ENSORLG00000008700
ENSORLT0000	CDS	CDS	ENSORLG00000005936
ENSORLT0000	CDS	CDS	ENSORLG00000000629
ENSORLT0000	CDS	CDS	ENSORLG00000015810
ENSORLT0000	CDS	CDS	ENSORLG00000008029
ENSORLT0000	CDS	CDS	ENSORLG00000012833
ENSORLT0000	CDS	CDS	ENSORLG00000011381
ENSORLT0000	CDS	CDS	ENSORLG00000012890
ENSORLT0000	CDS	CDS	ENSORLG00000010621
ENSORLT0000	CDS	CDS	ENSORLG00000016940
ENSORLT0000	CDS	CDS	ENSORLG00000020765
ENSORLT0000	CDS	CDS	ENSORLG00000010249
ENSORLT0000	CDS	CDS	ENSORLG00000004609
ENSORLT0000	CDS	CDS	ENSORLG00000010423
ENSORLT0000	CDS	CDS	ENSORLG00000015342
ENSORLT0000	CDS	CDS	ENSORLG00000009600
ENSORLT0000	CDS	CDS	ENSORLG00000010672
ENSORLT0000	CDS	CDS	ENSORLG00000001174
ENSORLT0000	CDS	CDS	ENSORLG00000006027
ENSORLT0000	CDS	CDS	ENSORLG00000008697
ENSORLT0000	CDS	CDS	ENSORLG00000011009
ENSORLT0000	CDS	CDS	ENSORLG00000011732
ENSORLT0000	CDS	CDS	ENSORLG00000011615
ENSORLT0000	CDS	CDS	ENSORLG00000004757
ENSORLT0000	CDS	CDS	ENSORLG00000004724
ENSORLT0000	CDS	CDS	ENSORLG00000017271
ENSORLT0000	CDS	CDS	ENSORLG00000004496
ENSORLT0000	CDS	CDS	ENSORLG00000012299
ENSORLT0000	CDS	CDS	ENSORLG00000008985
ENSORLT0000	CDS	CDS	ENSORLG00000009427
ENSORLT0000	CDS	CDS	ENSORLG00000003328
ENSORLT0000	CDS	CDS	ENSORLG00000009184
ENSORLT0000	CDS	CDS	ENSORLG00000009271
ENSORLT0000	CDS	CDS	ENSORLG00000010079
ENSORLT0000	CDS	CDS	ENSORLG00000017960
ENSORLT0000	CDS	CDS	ENSORLG00000012678
ENSORLT0000	CDS	CDS	ENSORLG00000006683
ENSORLT0000	CDS	CDS	ENSORLG00000011355
ENSORLT0000	CDS	CDS	ENSORLG00000003500
ENSORLT0000	CDS	CDS	ENSORLG00000006599
ENSORLT0000	CDS	CDS	ENSORLG00000015126
ENSORLT0000	CDS	CDS	ENSORLG00000012084
ENSORLT0000	CDS	CDS	ENSORLG00000015253
ENSORLT0000	CDS	CDS	ENSORLG00000002614
ENSORLT0000	CDS	CDS	ENSORLG00000018220
ENSORLT0000	CDS	CDS	ENSORLG00000002997
ENSORLT0000	CDS	CDS	ENSORLG00000010217
ENSORLT0000	CDS	CDS	ENSORLG00000004589

ENSORLT0000	CDS	CDS	ENSORLG00000008138
ENSORLT0000	CDS	CDS	ENSORLG00000015336
ENSORLT0000	CDS	CDS	ENSORLG00000008775
ENSORLT0000	CDS	CDS	ENSORLG00000013029
ENSORLT0000	CDS	CDS	ENSORLG00000007709
ENSORLT0000	CDS	CDS	ENSORLG00000014488
ENSORLT0000	CDS	CDS	ENSORLG00000018012
ENSORLT0000	CDS	CDS	ENSORLG00000018012
ENSORLT0000	CDS	CDS	ENSORLG00000018012
ENSORLT0000	CDS	CDS	ENSORLG00000008714
ENSORLT0000	CDS	CDS	ENSORLG00000008991
ENSORLT0000	CDS	CDS	ENSORLG00000003852
ENSORLT0000	CDS	CDS	ENSORLG00000009595
ENSORLT0000	CDS	CDS	ENSORLG00000013213
ENSORLT0000	CDS	CDS	ENSORLG00000010898
ENSORLT0000	CDS	CDS	ENSORLG00000005352
ENSORLT0000	CDS	CDS	ENSORLG00000003456
ENSORLT0000	CDS	CDS	ENSORLG00000010705
ENSORLT0000	CDS	CDS	ENSORLG00000007771
ENSORLT0000	CDS	CDS	ENSORLG00000001951
ENSORLT0000	CDS	CDS	ENSORLG00000007177
ENSORLT0000	CDS	CDS	ENSORLG00000020895
ENSORLT0000	CDS	CDS	ENSORLG00000017902
ENSORLT0000	CDS	CDS	ENSORLG00000002001
ENSORLT0000	CDS	CDS	ENSORLG00000010449
ENSORLT0000	CDS	CDS	ENSORLG00000009199
ENSORLT0000	CDS	CDS	ENSORLG00000007223
ENSORLT0000	CDS	CDS	ENSORLG00000014773
ENSORLT0000	CDS	CDS	ENSORLG00000008302
ENSORLT0000	CDS	CDS	ENSORLG00000014443
ENSORLT0000	CDS	CDS	ENSORLG00000011817
ENSORLT0000	CDS	CDS	ENSORLG00000017276
ENSORLT0000	CDS	CDS	ENSORLG00000005241
ENSORLT0000	CDS	CDS	ENSORLG00000001399
ENSORLT0000	CDS	CDS	ENSORLG00000007842
ENSORLT0000	CDS	CDS	ENSORLG00000011002
ENSORLT0000	CDS	CDS	ENSORLG00000002036
ENSORLT0000	CDS	CDS	ENSORLG00000014891
ENSORLT0000	CDS	CDS	ENSORLG00000016613
ENSORLT0000	CDS	CDS	ENSORLG00000010799
ENSORLT0000	CDS	CDS	ENSORLG00000018129
ENSORLT0000	CDS	CDS	ENSORLG00000016966
ENSORLT0000	CDS	CDS	ENSORLG00000017287
ENSORLT0000	CDS	CDS	ENSORLG00000019566
ENSORLT0000	CDS	CDS	ENSORLG00000008315
ENSORLT0000	CDS	CDS	ENSORLG00000008315
ENSORLT0000	CDS	CDS	ENSORLG00000012904
ENSORLT0000	CDS	CDS	ENSORLG00000004387
ENSORLT0000	CDS	CDS	ENSORLG00000004387
ENSORLT0000	CDS	CDS	ENSORLG00000009538
ENSORLT0000	CDS	CDS	ENSORLG00000005829
ENSORLT0000	CDS	CDS	ENSORLG00000009416

ENSORLT0000	CDS	CDS	ENSORLG00000006082
ENSORLT0000	CDS	CDS	ENSORLG00000012008
ENSORLT0000	CDS	CDS	ENSORLG00000017379
ENSORLT0000	CDS	CDS	ENSORLG00000002912
ENSORLT0000	CDS	CDS	ENSORLG00000007164
ENSORLT0000	CDS	CDS	ENSORLG00000004570
ENSORLT0000	CDS	CDS	ENSORLG00000003286
ENSORLT0000	CDS	CDS	ENSORLG00000003673
ENSORLT0000	CDS	CDS	ENSORLG00000005488
ENSORLT0000	CDS	CDS	ENSORLG00000008035
ENSORLT0000	CDS	CDS	ENSORLG00000001379
ENSORLT0000	CDS	CDS	ENSORLG00000016873
ENSORLT0000	CDS	CDS	ENSORLG00000016734
ENSORLT0000	CDS	CDS	ENSORLG00000014882
ENSORLT0000	CDS	CDS	ENSORLG00000003457
ENSORLT0000	CDS	CDS	ENSORLG00000018576
ENSORLT0000	CDS	CDS	ENSORLG00000018576
ENSORLT0000	CDS	CDS	ENSORLG00000006828
ENSORLT0000	CDS	CDS	ENSORLG00000016883
ENSORLT0000	CDS	CDS	ENSORLG00000004091
ENSORLT0000	CDS	CDS	ENSORLG00000013947
ENSORLT0000	CDS	CDS	ENSORLG00000005971
ENSORLT0000	CDS	CDS	ENSORLG00000011726
ENSORLT0000	CDS	CDS	ENSORLG00000004014
ENSORLT0000	CDS	CDS	ENSORLG00000006040
ENSORLT0000	CDS	CDS	ENSORLG00000002926
ENSORLT0000	CDS	CDS	ENSORLG00000002926
ENSORLT0000	CDS	CDS	ENSORLG00000000182
ENSORLT0000	CDS	CDS	ENSORLG00000012632
ENSORLT0000	CDS	CDS	ENSORLG00000013559
ENSORLT0000	CDS	CDS	ENSORLG00000018318
ENSORLT0000	CDS	CDS	ENSORLG00000015999
ENSORLT0000	CDS	CDS	ENSORLG00000013613
ENSORLT0000	CDS	CDS	ENSORLG00000009372
ENSORLT0000	CDS	CDS	ENSORLG00000001680
ENSORLT0000	CDS	CDS	ENSORLG00000003667
ENSORLT0000	CDS	CDS	ENSORLG00000003667
ENSORLT0000	CDS	CDS	ENSORLG00000005486
ENSORLT0000	CDS	CDS	ENSORLG00000003670
ENSORLT0000	CDS	CDS	ENSORLG00000014123
ENSORLT0000	CDS	CDS	ENSORLG00000005106
ENSORLT0000	CDS	CDS	ENSORLG00000005709
ENSORLT0000	CDS	CDS	ENSORLG00000015888
ENSORLT0000	CDS	CDS	ENSORLG00000012096
ENSORLT0000	CDS	CDS	ENSORLG00000020741
ENSORLT0000	CDS	CDS	ENSORLG00000008174
ENSORLT0000	CDS	CDS	ENSORLG00000011132
ENSORLT0000	CDS	CDS	ENSORLG00000020365
ENSORLT0000	CDS	CDS	ENSORLG00000020232
ENSORLT0000	CDS	CDS	ENSORLG00000016189
ENSORLT0000	CDS	CDS	ENSORLG00000010946
ENSORLT0000	CDS	CDS	ENSORLG00000008620

ENSORLT0000	CDS	CDS	ENSORLG00000002861
ENSORLT0000	CDS	CDS	ENSORLG00000014389
ENSORLT0000	CDS	CDS	ENSORLG00000020481
ENSORLT0000	CDS	CDS	ENSORLG00000013184
ENSORLT0000	CDS	CDS	ENSORLG00000003633
ENSORLT0000	CDS	CDS	ENSORLG00000003633
ENSORLT0000	CDS	CDS	ENSORLG00000008359
ENSORLT0000	CDS	CDS	ENSORLG00000010514
ENSORLT0000	CDS	CDS	ENSORLG00000012782
ENSORLT0000	CDS	CDS	ENSORLG00000019024
ENSORLT0000	CDS	CDS	ENSORLG00000010486
ENSORLT0000	CDS	CDS	ENSORLG00000013214
ENSORLT0000	CDS	CDS	ENSORLG00000014164
ENSORLT0000	CDS	CDS	ENSORLG00000007983
ENSORLT0000	CDS	CDS	ENSORLG00000017390
ENSORLT0000	CDS	CDS	ENSORLG00000009489
ENSORLT0000	CDS	CDS	ENSORLG00000009366
ENSORLT0000	CDS	CDS	ENSORLG00000015321
ENSORLT0000	CDS	CDS	ENSORLG00000011373
ENSORLT0000	CDS	CDS	ENSORLG00000009544
ENSORLT0000	CDS	CDS	ENSORLG00000002450
ENSORLT0000	CDS	CDS	ENSORLG00000011753
ENSORLT0000	CDS	CDS	ENSORLG00000013112
ENSORLT0000	CDS	CDS	ENSORLG00000017079
ENSORLT0000	CDS	CDS	ENSORLG00000005598
ENSORLT0000	CDS	CDS	ENSORLG00000007487
ENSORLT0000	CDS	CDS	ENSORLG00000011356
ENSORLT0000	CDS	CDS	ENSORLG00000020191
ENSORLT0000	CDS	CDS	ENSORLG00000015037
ENSORLT0000	CDS	CDS	ENSORLG00000000799
ENSORLT0000	cDNA	cDNA	ENSORLG00000000607
ENSORLT0000	CDNA	cDNA	ENSORLG00000013823
ENSORLT0000	CDS	CDS	ENSORLG00000000705
ENSORLT0000	CDS	CDS	ENSORLG00000001238
ENSORLT0000	CDS	CDS	ENSORLG00000001697
ENSORLT0000	5UTR	UTR5	ENSORLG00000002043
ENSORLT0000	CDS	CDS	ENSORLG00000002231
ENSORLT0000	CDS	CDS	ENSORLG00000004593
ENSORLT0000	CDS	CDS	ENSORLG00000005260
ENSORLT0000	CDS	CDS	ENSORLG00000005616
ENSORLT0000	CDS	CDS	ENSORLG00000005639
ENSORLT0000	CDS	CDS	ENSORLG00000005770
ENSORLT0000	CDS	CDS	ENSORLG00000006118
ENSORLT0000	CDS	CDS	ENSORLG00000006140
ENSORLT0000	CDS	CDS	ENSORLG00000006467
ENSORLT0000	CDS	CDS	ENSORLG00000007035
ENSORLT0000	CDS	CDS	ENSORLG00000008344
ENSORLT0000	CDS	CDS	ENSORLG00000010637
ENSORLT0000	CDS	CDS	ENSORLG00000010891
ENSORLT0000	CDS	CDS	ENSORLG00000000035
ENSORLT0000	3UTR	UTR3	ENSORLG00000000227
ENSORLT0000	CDS	CDS	ENSORLG00000001051

ENSORLT0000	CDS	CDS	ENSORLG00000001051
ENSORLT0000	CDS	CDS	ENSORLG00000001322
ENSORLT0000	CDS	CDS	ENSORLG00000001801
ENSORLT0000	CDS	CDS	ENSORLG00000001869
ENSORLT0000	CDS	CDS	ENSORLG00000004201
ENSORLT0000	CDS	CDS	ENSORLG00000004413
ENSORLT0000	CDS	CDS	ENSORLG00000004967
ENSORLT0000	CDS	CDS	ENSORLG00000005223
ENSORLT0000	CDS	CDS	ENSORLG00000005337
ENSORLT0000	CDS	CDS	ENSORLG00000005533
ENSORLT0000	CDS	CDS	ENSORLG00000006059
ENSORLT0000	CDS	CDS	ENSORLG00000006146
ENSORLT0000	CDS	CDS	ENSORLG00000006146
ENSORLT0000	CDS	CDS	ENSORLG00000006233
ENSORLT0000	CDS	CDS	ENSORLG00000006425
ENSORLT0000	CDS	CDS	ENSORLG00000009071
ENSORLT0000	CDS	CDS	ENSORLG00000009187
ENSORLT0000	CDS	CDS	ENSORLG00000009995
ENSORLT0000	CDS	CDS	ENSORLG00000010198
ENSORLT0000	CDS	CDS	ENSORLG00000008177
ENSORLT0000	CDS	CDS	ENSORLG00000000073
ENSORLT0000	CDS	CDS	ENSORLG00000000514
ENSORLT0000	CDS	CDS	ENSORLG00000003470
ENSORLT0000	3UTR	UTR3	ENSORLG00000006874
ENSORLT0000	CDS	CDS	ENSORLG00000007097
ENSORLT0000	CDS	CDS	ENSORLG00000007178
ENSORLT0000	CDS	CDS	ENSORLG00000007224
ENSORLT0000	CDS	CDS	ENSORLG00000009018
ENSORLT0000	CDS	CDS	ENSORLG00000009359
ENSORLT0000	CDS	CDS	ENSORLG00000000282
ENSORLT0000	3UTR	UTR3	ENSORLG00000000877
ENSORLT0000	CDS	CDS	ENSORLG00000001531
ENSORLT0000	CDS	CDS	ENSORLG00000002133
ENSORLT0000	CDS	CDS	ENSORLG00000004073
ENSORLT0000	CDS	CDS	ENSORLG00000004270
ENSORLT0000	CDS	CDS	ENSORLG00000005966
ENSORLT0000	CDS	CDS	ENSORLG00000006254
ENSORLT0000	5UTR	UTR5	ENSORLG00000009826
ENSORLT0000	CDS	CDS	ENSORLG00000010390
ENSORLT0000	CDS	CDS	ENSORLG00000001664
ENSORLT0000	CDS	CDS	ENSORLG00000002124
ENSORLT0000	CDS	CDS	ENSORLG00000003065
ENSORLT0000	CDS	CDS	ENSORLG00000007843
ENSORLT0000	CDS	CDS	ENSORLG00000008060
ENSORLT0000	CDS	CDS	ENSORLG00000009880
ENSORLT0000	CDS	CDS	ENSORLG00000010363
ENSORLT0000	CDS	CDS	ENSORLG00000010944
ENSORLT0000	CDS	CDS	ENSORLG00000011057
ENSORLT0000	CDS	CDS	ENSORLG00000011584
ENSORLT0000	3UTR	UTR3	ENSORLG00000012363
ENSORLT0000	3UTR	UTR3	ENSORLG00000012745
ENSORLT0000	CDS	CDS	ENSORLG00000013011

ENSORLT0000	CDS	CDS	ENSORLG00000013519
ENSORLT0000	CDS	CDS	ENSORLG00000013780
ENSORLT0000	CDS	CDS	ENSORLG00000014327
ENSORLT0000	CDS	CDS	ENSORLG00000014328
ENSORLT0000	CDS	CDS	ENSORLG00000015757
ENSORLT0000	CDS	CDS	ENSORLG00000016591
ENSORLT0000	CDS	CDS	ENSORLG00000016749
ENSORLT0000	CDS	CDS	ENSORLG00000017703
ENSORLT0000	CDS	CDS	ENSORLG00000014358
ENSORLT0000	CDS	CDS	ENSORLG00000014880
ENSORLT0000	CDS	CDS	ENSORLG00000014919
ENSORLT0000	5UTR	UTR5	ENSORLG00000015122
ENSORLT0000	CDS	CDS	ENSORLG00000016025
ENSORLT0000	CDS	CDS	ENSORLG00000016025
ENSORLT0000	CDS	CDS	ENSORLG00000016679
ENSORLT0000	CDS	CDS	ENSORLG00000016797
ENSORLT0000	CDS	CDS	ENSORLG00000018021
ENSORLT0000	CDS	CDS	ENSORLG00000018122
ENSORLT0000	CDS	CDS	ENSORLG00000018232
ENSORLT0000	CDS	CDS	ENSORLG00000018257
ENSORLT0000	CDS	CDS	ENSORLG00000012830
ENSORLT0000	CDS	CDS	ENSORLG00000013248
ENSORLT0000	CDS	CDS	ENSORLG00000014433
ENSORLT0000	CDS	CDS	ENSORLG00000014888
ENSORLT0000	CDS	CDS	ENSORLG00000015151
ENSORLT0000	CDS	CDS	ENSORLG00000017067
ENSORLT0000	CDS	CDS	ENSORLG00000017454
ENSORLT0000	CDS	CDS	ENSORLG00000018320
ENSORLT0000	CDS	CDS	ENSORLG00000018644
ENSORLT0000	CDS	CDS	ENSORLG00000018644
ENSORLT0000	CDS	CDS	ENSORLG00000018900
ENSORLT0000	CDS	CDS	ENSORLG00000019206
ENSORLT0000	CDS	CDS	ENSORLG00000019382
ENSORLT0000	CDNA	cDNA	ENSORLG00000019656
ENSORLT0000	CDS	CDS	ENSORLG00000020274
ENSORLT0000	CDS	CDS	ENSORLG00000020371
ENSORLT0000	5UTR	UTR5	ENSORLG00000020834
ENSORLT0000	5UTR	UTR5	ENSORLG00000020834
ENSORLT0000	CDS	CDS	ENSORLG00000015888
ENSORLT0000	CDS	CDS	ENSORLG00000015888
ENSORLT0000	CDS	CDS	ENSORLG00000017079
ENSORLT0000	CDS	CDS	ENSORLG00000011260
ENSORLT0000	CDS	CDS	ENSORLG00000003852
ENSORLT0000	CDS	CDS	ENSORLG00000008714
ENSORLT0000	CDS	CDS	ENSORLG00000001164
ENSORLT0000	CDS	CDS	ENSORLG00000005066
ENSORLT0000	3UTR	UTR3	ENSORLG00000017388
ENSORLT0000	CDS	CDS	ENSORLG00000004573
ENSORLT0000	3UTR	UTR3	ENSORLG00000003787
ENSORLT0000	CDS	CDS	ENSORLG00000009113
ENSORLT0000	3UTR	CDS	ENSORLG00000010905
ENSORLT0000	CDS	CDS	ENSORLG00000001478

ENSORLT0000	CDS	CDS	ENSORLG00000001884
ENSORLT0000	CDS	CDS	ENSORLG00000002336
ENSORLT0000	CDS	CDS	ENSORLG00000002336
ENSORLT0000	CDS	CDS	ENSORLG00000002422
ENSORLT0000	CDS	CDS	ENSORLG00000003690
ENSORLT0000	CDS	CDS	ENSORLG00000004897
ENSORLT0000	CDS	CDS	ENSORLG00000005005
ENSORLT0000	CDS	CDS	ENSORLG00000005709
ENSORLT0000	CDS	CDS	ENSORLG00000005709
ENSORLT0000	CDS	CDS	ENSORLG00000001225
ENSORLT0000	CDS	CDS	ENSORLG00000002627
ENSORLT0000	CDS	CDS	ENSORLG00000002854
ENSORLT0000	CDS	CDS	ENSORLG00000003520
ENSORLT0000	CDS	CDS	ENSORLG00000003520
ENSORLT0000	CDS	CDS	ENSORLG00000008548
ENSORLT0000	CDS	CDS	ENSORLG00000008878
ENSORLT0000	CDS	CDS	ENSORLG00000005372
ENSORLT0000	CDS	CDS	ENSORLG00000005658
ENSORLT0000	CDS	CDS	ENSORLG00000005675
ENSORLT0000	CDS	CDS	ENSORLG00000005675
ENSORLT0000	CDS	CDS	ENSORLG00000005678
ENSORLT0000	CDS	CDS	ENSORLG00000006082
ENSORLT0000	CDS	CDS	ENSORLG00000006093
ENSORLT0000	5UTR	UTR5	ENSORLG00000006109
ENSORLT0000	CDS	CDS	ENSORLG00000006171
ENSORLT0000	CDS	CDS	ENSORLG00000006683
ENSORLT0000	CDS	CDS	ENSORLG00000001079
ENSORLT0000	CDS	CDS	ENSORLG00000002231
ENSORLT0000	CDS	CDS	ENSORLG00000008344
ENSORLT0000	CDS	CDS	ENSORLG00000008374
ENSORLT0000	CDS	CDS	ENSORLG00000008374
ENSORLT0000	CDS	CDS	ENSORLG00000008374
ENSORLT0000	CDS	CDS	ENSORLG00000008111
ENSORLT0000	CDS	CDS	ENSORLG00000010348
ENSORLT0000	CDS	CDS	ENSORLG00000002001
ENSORLT0000	CDS	CDS	ENSORLG00000002342
ENSORLT0000	CDS	CDS	ENSORLG00000002427
ENSORLT0000	CDS	CDS	ENSORLG00000002898
ENSORLT0000	CDS	CDS	ENSORLG00000002904
ENSORLT0000	CDS	CDS	ENSORLG00000003456
ENSORLT0000	CDS	CDS	ENSORLG00000003838
ENSORLT0000	CDS	CDS	ENSORLG00000003838
ENSORLT0000	CDS	CDS	ENSORLG00000006079
ENSORLT0000	CDS	CDS	ENSORLG00000002926
ENSORLT0000	CDS	CDS	ENSORLG00000003138
ENSORLT0000	CDS	CDS	ENSORLG00000006341
ENSORLT0000	CDS	CDS	ENSORLG00000006782
ENSORLT0000	CDS	CDS	ENSORLG00000006899
ENSORLT0000	CDS	CDS	ENSORLG00000007836
ENSORLT0000	CDS	CDS	ENSORLG00000008478
ENSORLT0000	CDS	CDS	ENSORLG00000008478
ENSORLT0000	CDS	CDS	ENSORLG00000006012

ENSORLT0000	CDS	CDS	ENSORLG00000009671
ENSORLT0000	CDS	CDS	ENSORLG00000006914
ENSORLT0000	CDS	CDS	ENSORLG00000007164
ENSORLT0000	CDS	CDS	ENSORLG00000007304
ENSORLT0000	CDS	CDS	ENSORLG00000007727
ENSORLT0000	CDS	CDS	ENSORLG00000008057
ENSORLT0000	CDS	CDS	ENSORLG00000008153
ENSORLT0000	CDS	CDS	ENSORLG00000003006
ENSORLT0000	CDS	CDS	ENSORLG00000003589
ENSORLT0000	CDS	CDS	ENSORLG00000004339
ENSORLT0000	CDS	CDS	ENSORLG00000005352
ENSORLT0000	CDS	CDS	ENSORLG00000005535
ENSORLT0000	CDS	CDS	ENSORLG00000009590
ENSORLT0000	CDS	CDS	ENSORLG00000010171
ENSORLT0000	CDS	CDS	ENSORLG00000000913
ENSORLT0000	CDS	CDS	ENSORLG00000002730
ENSORLT0000	CDS	CDS	ENSORLG00000009604
ENSORLT0000	CDS	CDS	ENSORLG00000003670
ENSORLT0000	CDS	CDS	ENSORLG00000004548
ENSORLT0000	CDS	CDS	ENSORLG00000007983
ENSORLT0000	CDS	CDS	ENSORLG00000008354
ENSORLT0000	CDS	CDS	ENSORLG00000008700
ENSORLT0000	CDS	CDS	ENSORLG00000009088
ENSORLT0000	CDS	CDS	ENSORLG00000009256
ENSORLT0000	CDS	CDS	ENSORLG00000010136
ENSORLT0000	CDS	CDS	ENSORLG00000010396
ENSORLT0000	CDS	CDS	ENSORLG00000000073
ENSORLT0000	CDS	CDS	ENSORLG00000000073
ENSORLT0000	CDS	CDS	ENSORLG00000000073
ENSORLT0000	CDS	CDS	ENSORLG00000002699
ENSORLT0000	CDS	CDS	ENSORLG00000003245
ENSORLT0000	CDS	CDS	ENSORLG00000007672
ENSORLT0000	CDS	CDS	ENSORLG00000000182
ENSORLT0000	CDS	CDS	ENSORLG00000002312
ENSORLT0000	CDS	CDS	ENSORLG00000003500
ENSORLT0000	CDS	CDS	ENSORLG00000005344
ENSORLT0000	CDS	CDS	ENSORLG00000005502
ENSORLT0000	CDS	CDS	ENSORLG00000006499
ENSORLT0000	CDS	CDS	ENSORLG00000006870
ENSORLT0000	CDS	CDS	ENSORLG00000007701
ENSORLT0000	CDS	CDS	ENSORLG00000007980
ENSORLT0000	CDS	CDS	ENSORLG00000008937
ENSORLT0000	CDS	CDS	ENSORLG00000010799
ENSORLT0000	CDS	CDS	ENSORLG00000004613
ENSORLT0000	CDS	CDS	ENSORLG00000010732
ENSORLT0000	5UTR	UTR5	ENSORLG00000002648
ENSORLT0000	CDS	CDS	ENSORLG00000006803
ENSORLT0000	CDS	CDS	ENSORLG00000007431
ENSORLT0000	CDS	CDS	ENSORLG00000007431
ENSORLT0000	CDS	CDS	ENSORLG00000007525
ENSORLT0000	CDS	CDS	ENSORLG00000007525
ENSORLT0000	CDS	CDS	ENSORLG00000007525

ENSORLT0000	CDS	CDS	ENSORLG00000008337
ENSORLT0000	CDS	CDS	ENSORLG00000010119
ENSORLT0000	CDS	CDS	ENSORLG00000010778
ENSORLT0000	CDS	CDS	ENSORLG00000010778
ENSORLT0000	CDS	CDS	ENSORLG00000002686
ENSORLT0000	5UTR	UTR5	ENSORLG00000008464
ENSORLT0000	CDS	CDS	ENSORLG00000008616
ENSORLT0000	CDS	CDS	ENSORLG00000010390
ENSORLT0000	CDS	CDS	ENSORLG00000001284
ENSORLT0000	CDS	CDS	ENSORLG00000004453
ENSORLT0000	CDS	CDS	ENSORLG00000007952
ENSORLT0000	CDS	CDS	ENSORLG00000010741
ENSORLT0000	CDS	CDS	ENSORLG00000010741
ENSORLT0000	CDS	CDS	ENSORLG00000011554
ENSORLT0000	CDS	CDS	ENSORLG00000011554
ENSORLT0000	CDS	CDS	ENSORLG00000011579
ENSORLT0000	CDS	CDS	ENSORLG00000012270
ENSORLT0000	CDS	CDS	ENSORLG00000012678
ENSORLT0000	CDS	CDS	ENSORLG00000012680
ENSORLT0000	CDS	CDS	ENSORLG00000012833
ENSORLT0000	CDS	CDS	ENSORLG00000012833
ENSORLT0000	CDS	CDS	ENSORLG00000012833
ENSORLT0000	CDS	CDS	ENSORLG00000014325
ENSORLT0000	CDS	CDS	ENSORLG00000014518
ENSORLT0000	CDS	CDS	ENSORLG00000014631
ENSORLT0000	CDS	CDS	ENSORLG00000014884
ENSORLT0000	CDS	CDS	ENSORLG00000015508
ENSORLT0000	CDS	CDS	ENSORLG00000012299
ENSORLT0000	CDS	CDS	ENSORLG00000012299
ENSORLT0000	CDS	CDS	ENSORLG00000012632
ENSORLT0000	CDS	CDS	ENSORLG00000012680
ENSORLT0000	CDS	CDS	ENSORLG00000013112
ENSORLT0000	CDS	CDS	ENSORLG00000013815
ENSORLT0000	CDS	CDS	ENSORLG00000014164
ENSORLT0000	5UTR	UTR5	ENSORLG00000011858
ENSORLT0000	CDNA	cDNA	ENSORLG00000012724
ENSORLT0000	CDS	CDS	ENSORLG00000014576
ENSORLT0000	CDS	CDS	ENSORLG00000014781
ENSORLT0000	CDS	CDS	ENSORLG00000014810
ENSORLT0000	CDS	CDS	ENSORLG00000015037
ENSORLT0000	CDS	CDS	ENSORLG00000016157
ENSORLT0000	CDS	CDS	ENSORLG00000016189
ENSORLT0000	CDS	CDS	ENSORLG00000016224
ENSORLT0000	CDS	CDS	ENSORLG00000012776
ENSORLT0000	CDS	CDS	ENSORLG00000012779
ENSORLT0000	3UTR	UTR3	ENSORLG00000013527
ENSORLT0000	3UTR	UTR5	ENSORLG00000013527
ENSORLT0000	5UTR	UTR3	ENSORLG00000013527
ENSORLT0000	5UTR	UTR5	ENSORLG00000013527
ENSORLT0000	CDS	CDS	ENSORLG00000013992
ENSORLT0000	CDS	CDS	ENSORLG00000014129
ENSORLT0000	CDS	CDS	ENSORLG00000014469

ENSORLT0000	CDS	CDS	ENSORLG00000015563
ENSORLT0000	CDS	CDS	ENSORLG00000016915
ENSORLT0000	CDS	CDS	ENSORLG00000012076
ENSORLT0000	CDS	CDS	ENSORLG00000013744
ENSORLT0000	CDS	CDS	ENSORLG00000013827
ENSORLT0000	CDS	CDS	ENSORLG00000014338
ENSORLT0000	CDS	CDS	ENSORLG00000016946
ENSORLT0000	CDS	CDS	ENSORLG00000017465
ENSORLT0000	CDS	CDS	ENSORLG00000017960
ENSORLT0000	CDS	CDS	ENSORLG00000012890
ENSORLT0000	CDS	CDS	ENSORLG00000012890
ENSORLT0000	CDS	CDS	ENSORLG00000015273
ENSORLT0000	CDS	CDS	ENSORLG00000015742
ENSORLT0000	CDS	CDS	ENSORLG00000015767
ENSORLT0000	CDS	CDS	ENSORLG00000017234
ENSORLT0000	CDS	CDS	ENSORLG00000017804
ENSORLT0000	5UTR	UTR5	ENSORLG00000012199
ENSORLT0000	CDS	CDS	ENSORLG00000013616
ENSORLT0000	CDS	CDS	ENSORLG00000013991
ENSORLT0000	CDS	CDS	ENSORLG00000015436
ENSORLT0000	CDS	CDS	ENSORLG00000016735
ENSORLT0000	3UTR	UTR3	ENSORLG00000017948
ENSORLT0000	CDS	CDS	ENSORLG00000015040
ENSORLT0000	CDS	CDS	ENSORLG00000015040
ENSORLT0000	CDS	CDS	ENSORLG00000015913
ENSORLT0000	3UTR	UTR3	ENSORLG00000016165
ENSORLT0000	CDS	CDS	ENSORLG00000016305
ENSORLT0000	CDS	CDS	ENSORLG00000016915
ENSORLT0000	CDS	CDS	ENSORLG00000017764
ENSORLT0000	CDS	CDS	ENSORLG00000014443
ENSORLT0000	CDS	CDS	ENSORLG00000015683
ENSORLT0000	CDS	CDS	ENSORLG00000013379
ENSORLT0000	CDS	CDS	ENSORLG00000014287
ENSORLT0000	CDS	CDS	ENSORLG00000015151
ENSORLT0000	CDS	CDS	ENSORLG00000015462
ENSORLT0000	CDS	CDS	ENSORLG00000016883
ENSORLT0000	CDS	CDS	ENSORLG00000016883
ENSORLT0000	5UTR	UTR5	ENSORLG00000018531
ENSORLT0000	CDS	CDS	ENSORLG00000018794
ENSORLT0000	CDS	CDS	ENSORLG00000018938
ENSORLT0000	CDS	CDS	ENSORLG00000019403
ENSORLT0000	3UTR	UTR3	ENSORLG00000020656
ENSORLT0000	CDS	CDS	ENSORLG00000019583
ENSORLT0000	CDS	CDS	ENSORLG00000018341
ENSORLT0000	CDNA	cDNA	ENSORLG00000018644
ENSORLT0000	CDS	CDS	ENSORLG00000018679
ENSORLT0000	CDS	CDS	ENSORLG00000019927
ENSORLT0000	CDNA	cDNA	ENSORLG00000020257
ENSORLT0000	CDS	CDS	ENSORLG00000020371

Gene common name
uracil phosphoribosyltransferase (FUR1) homolog (S. cerevis
UDP-glucose glycoprotein glucosyltransferase 1 [Source:ZFII
SUZ RNA binding domain containing 1 [Source:HGNC Symbo
septin 10 [Source:ZFIN;Acc:ZDB-GENE-050417-3]
ring finger protein 170 [Source:ZFIN;Acc:ZDB-GENE-040426-
RAB18B, member RAS oncogene family [Source:ZFIN;Acc:ZD
pdp2
oxysterol binding protein-like 2b [Source:ZFIN;Acc:ZDB-GEN
NT5C3B (1 of many)
itpk1b
growth factor receptor-bound protein 2b [Source:ZFIN;Acc:
growth factor receptor-bound protein 2b [Source:ZFIN;Acc:
dusp1
charged multivesicular body protein 2Ba [Source:ZFIN;Acc:Z
basigin [Source:ZFIN;Acc:ZDB-GENE-030131-9638]
voltage-dependent anion channel 1 [Source:ZFIN;Acc:ZDB-G
proteasome subunit beta 1 [Source:ZFIN;Acc:ZDB-GENE-040
prkaa1
pirin [Source:ZFIN;Acc:ZDB-GENE-040718-288]
Oryzias latipes signal transducer and activator of transcripti
nudix hydrolase 13 [Source:HGNC Symbol;Acc:HGNC:18827]
NECAP endocytosis associated 2 [Source:ZFIN;Acc:ZDB-GENI
laccase (multicopper oxidoreductase) domain containing 1 [
intraflagellar transport 52 homolog (Chlamydomonas) [Sour
fkbp1ab
fkbp1aa
DEAD (Asp-Glu-Ala-Asp) box helicase 3a [Source:ZFIN;Acc:ZI
clock circadian regulator b [Source:ZFIN;Acc:ZDB-GENE-030
capping actin protein of muscle Z-line alpha subunit 2 [Sour
AHA1, activator of heat shock protein ATPase homolog 1b [S
adcy7
acyl-CoA synthetase short chain family member 3 [Source:ZI
aconitase 1, soluble [Source:ZFIN;Acc:ZDB-GENE-031118-76
zmp:0000000846 [Source:ZFIN;Acc:ZDB-GENE-130530-849]
zinc finger protein 711 [Source:ZFIN;Acc:ZDB-GENE-081107-
zinc finger protein 592 [Source:ZFIN;Acc:ZDB-GENE-030131-
zinc finger protein 292a [Source:ZFIN;Acc:ZDB-GENE-07100
zinc finger homeobox 4 [Source:ZFIN;Acc:ZDB-GENE-07053C
zinc finger homeobox 3 [Source:ZFIN;Acc:ZDB-GENE-030131
zinc finger CCCH-type containing 18 [Source:ZFIN;Acc:ZDB-G
zinc finger and BTB domain containing 5 [Source:HGNC Sym
zic family member 1 (odd-paired homolog, Drosophila) [Sou
zgc:66449 [Source:ZFIN;Acc:ZDB-GENE-030131-5618]
zgc:56231 [Source:ZFIN;Acc:ZDB-GENE-040426-1914]
zgc:136929 [Source:ZFIN;Acc:ZDB-GENE-060421-4235]
zgc:110269 [Source:ZFIN;Acc:ZDB-GENE-050417-81]
YTH N(6)-methyladenosine RNA binding protein 2 [Source:Z
WSC domain containing 1b [Source:ZFIN;Acc:ZDB-GENE-091
wolframin ER transmembrane glycoprotein [Source:HGNC S
WNK4
Wiskott-Aldrich syndrome (eczema-thrombocytopenia) a [S

WD repeat domain 47a [Source:ZFIN;Acc:ZDB-GENE-070216
WD repeat and FYVE domain containing 3 [Source:ZFIN;Acc:
WD repeat and FYVE domain containing 1 [Source:ZFIN;Acc:
von Willebrand factor A domain containing 2 [Source:ZFIN;A
very low density lipoprotein receptor [Source:ZFIN;Acc:ZDB-
valosin containing protein [Source:ZFIN;Acc:ZDB-GENE-0301
utrophin [Source:ZFIN;Acc:ZDB-GENE-070228-6]
usp16
uroporphyrinogen decarboxylase [Source:ZFIN;Acc:ZDB-GEN
unc-50 homolog (C. elegans) [Source:ZFIN;Acc:ZDB-GENE-04
UDP-glucose glycoprotein glucosyltransferase 2 [Source:ZFI
tumor necrosis factor receptor superfamily, member 21 [So
tudor domain containing 1 [Source:ZFIN;Acc:ZDB-GENE-070
tubulin, alpha 8 like 3 [Source:ZFIN;Acc:ZDB-GENE-040801-7
Tu translation elongation factor, mitochondrial [Source:ZFIN
tRNA methyltransferase 11 homolog (S. cerevisiae) [Source:
tRNA aspartic acid methyltransferase 1 [Source:ZFIN;Acc:ZD
tRNA aspartic acid methyltransferase 1 [Source:ZFIN;Acc:ZD
tripartite motif containing 66 [Source:ZFIN;Acc:ZDB-GENE-0
trip12
trichorhinophalangeal syndrome I [Source:ZFIN;Acc:ZDB-GE
trhde.2
transportin 1 [Source:ZFIN;Acc:ZDB-GENE-080819-1]
transmembrane p24 trafficking protein 4 [Source:ZFIN;Acc:Z
transient receptor potential cation channel, subfamily M, m
transient receptor potential cation channel, subfamily M, m
transient receptor potential cation channel, subfamily C, me
transient receptor potential cation channel, subfamily A, me
transgelin 2 [Source:ZFIN;Acc:ZDB-GENE-020802-2]
transforming growth factor, beta 2, like [Source:ZFIN;Acc:ZD
transducin (beta)-like 2 [Source:ZFIN;Acc:ZDB-GENE-030131
tight junction protein 1b [Source:ZFIN;Acc:ZDB-GENE-07092
THUMP domain containing 3 [Source:ZFIN;Acc:ZDB-GENE-03
tetraspanin 33a [Source:ZFIN;Acc:ZDB-GENE-040718-361]
teneurin transmembrane protein 3 [Source:HGNC Symbol;A
TBC1 domain family, member 30 [Source:ZFIN;Acc:ZDB-GEN
TAF1 RNA polymerase II, TATA box binding protein (TBP)-as
synaptotagmin X [Source:ZFIN;Acc:ZDB-GENE-041210-297]
syk
SWI/SNF related, matrix associated, actin dependent regula
sushi, nidogen and EGF-like domains 1 [Source:ZFIN;Acc:ZD
sulfotransferase family, cytosolic, 6b, member 1 [Source:ZFI
structural maintenance of chromosomes 2 [Source:ZFIN;Acc
STIM activating enhance [Source:ZFIN;Acc:ZDB-GENE-04042
stearoyl-CoA desaturase (delta-9-desaturase) [Source:ZFIN;
SRY (sex determining region Y)-box 18 [Source:ZFIN;Acc:ZD
splA/ryanodine receptor domain and SOCS box containing 3
spinster homolog 3 (Drosophila) [Source:ZFIN;Acc:ZDB-GEN
sphingolipid transporter 2 [Source:HGNC Symbol;Acc:HGNC
spermatogenesis associated 5-like 1 [Source:ZFIN;Acc:ZDB-
spectrin, beta, erythrocytic [Source:ZFIN;Acc:ZDB-GENE-000
spalt-like transcription factor 1a [Source:ZFIN;Acc:ZDB-GEN

sorting nexin 2 [Source:ZFIN;Acc:ZDB-GENE-051120-45]
solute carrier organic anion transporter family, member 1C1
solute carrier family 7 (cationic amino acid transporter, y+ sy
solute carrier family 6 (neurotransmitter transporter), mem
solute carrier family 4 member 10 [Source:HGNC Symbol;Ac
solute carrier family 38, member 11 [Source:ZFIN;Acc:ZDB-G
solute carrier family 35, member E3 [Source:ZFIN;Acc:ZDB-G
solute carrier family 30 (zinc transporter), member 2 [Sourc
solute carrier family 27 (fatty acid transporter), member 2a
solute carrier family 2 member 4 [Source:HGNC Symbol;Acc
solute carrier family 2 (facilitated glucose transporter), mem
solute carrier family 16, member 7 (monocarboxylic acid tra
small nuclear ribonucleoprotein 27 (U4/U6.U5) [Source:ZFIN
SMAD family member 3b [Source:ZFIN;Acc:ZDB-GENE-03012
SMAD family member 2 [Source:ZFIN;Acc:ZDB-GENE-990603
slit homolog 3 (Drosophila) [Source:ZFIN;Acc:ZDB-GENE-010
single stranded DNA binding protein 3b [Source:ZFIN;Acc:ZD
signal-induced proliferation-associated 1 like 3 [Source:ZFIN
signal transducer and activator of transcription 4 [Source:ZF
si:dkey-243i1.1 [Source:ZFIN;Acc:ZDB-GENE-030131-6399]
si:dkey-183c6.8 [Source:ZFIN;Acc:ZDB-GENE-160728-23]
si:dkey-166k12.1 [Source:ZFIN;Acc:ZDB-GENE-120215-78]
si:dkey-160o24.3
si:ch73-170l17.1
si:ch211-79l17.1 [Source:ZFIN;Acc:ZDB-GENE-050419-127]
si:ch211-217a12.1 [Source:ZFIN;Acc:ZDB-GENE-121214-95]
si:ch211-160d20.3 [Source:ZFIN;Acc:ZDB-GENE-030131-890
si:ch211-14a17.6 [Source:ZFIN;Acc:ZDB-GENE-030616-589]
si:ch211-125m10.6 [Source:ZFIN;Acc:ZDB-GENE-120709-92]
si:ch1073-335m2.2 [Source:ZFIN;Acc:ZDB-GENE-081104-82]
si:ch1073-228j22.2 [Source:ZFIN;Acc:ZDB-GENE-110411-227
short chain dehydrogenase/reductase family 16C, member 5
short chain dehydrogenase/reductase family 16C, member 5
sephs1
sema domain, transmembrane domain (TM), and cytoplasm
SAS-6 centriolar assembly protein [Source:ZFIN;Acc:ZDB-GE
ryanodine receptor 3 [Source:HGNC Symbol;Acc:HGNC:1048
ryanodine receptor 2b (cardiac) [Source:ZFIN;Acc:ZDB-GENE
rngtt
ring finger protein 24 [Source:ZFIN;Acc:ZDB-GENE-041114-4
ring finger protein 111 [Source:ZFIN;Acc:ZDB-GENE-070808-
rhomboid 5 homolog 2 [Source:HGNC Symbol;Acc:HGNC:20
Rho GTPase activating protein 35a [Source:ZFIN;Acc:ZDB-GE
Rho GTPase activating protein 35a [Source:ZFIN;Acc:ZDB-GE
Rho GTPase activating protein 17b [Source:ZFIN;Acc:ZDB-GE
Rh family, C glycoprotein b [Source:ZFIN;Acc:ZDB-GENE-040
rev3l
retinoid x receptor, beta a [Source:ZFIN;Acc:ZDB-GENE-9805
regulatory factor X7b [Source:ZFIN;Acc:ZDB-GENE-170623-1
regucalcin [Source:ZFIN;Acc:ZDB-GENE-040718-68]
RecQ helicase-like 5 [Source:ZFIN;Acc:ZDB-GENE-071214-1]
Ras association domain family member 8 [Source:HGNC Syn

RAP1 GTPase activating protein 2a [Source:ZFIN;Acc:ZDB-GE
Ral GTPase activating protein, beta subunit (non-catalytic) [S
Ral GTPase activating protein, alpha subunit 2 (catalytic) [So
RAB38, member RAS oncogene family [Source:HGNC Symbo
RAB30, member RAS oncogene family [Source:ZFIN;Acc:ZDB
RAB25, member RAS oncogene family b [Source:ZFIN;Acc:ZD
RAB14, member RAS oncogene family [Source:ZFIN;Acc:ZDB
RAB14, member RAS oncogene family [Source:ZFIN;Acc:ZDB
RAB10, member RAS oncogene family [Source:ZFIN;Acc:ZDB
Rab interacting lysosomal protein-like 2 [Source:ZFIN;Acc:ZD
Rab interacting lysosomal protein-like 2 [Source:ZFIN;Acc:ZD
R3H domain containing 2 [Source:HGNC Symbol;Acc:HGNC:
pyrimidinergic receptor P2Y6 [Source:HGNC Symbol;Acc:HG
ptgr2
prpf4bb
protein phosphatase 2 regulatory subunit B'gamma [Source
protein O-fucosyltransferase 2 [Source:ZFIN;Acc:ZDB-GENE-
protein kinase, DNA-activated, catalytic polypeptide [Source
proteasome subunit beta 6 [Source:ZFIN;Acc:ZDB-GENE-990
proteasome subunit beta 6 [Source:ZFIN;Acc:ZDB-GENE-990
proteasome 26S subunit, ATPase 1a [Source:ZFIN;Acc:ZDB-G
prospero homeobox 1a [Source:ZFIN;Acc:ZDB-GENE-980526
proline and serine rich 1 [Source:ZFIN;Acc:ZDB-GENE-11100
proline and serine rich 1 [Source:ZFIN;Acc:ZDB-GENE-11100
ppifb
ppic
potassium channel, voltage gated eag related subfamily H, r
potassium channel tetramerization domain containing 6b [S
polybromo 1 [Source:ZFIN;Acc:ZDB-GENE-080926-4]
polq
plexin b2b [Source:ZFIN;Acc:ZDB-GENE-080902-1]
pkd1l2a
pirin [Source:ZFIN;Acc:ZDB-GENE-040718-288]
phospholipase A2, group VI (cytosolic, calcium-independent
phospholipase A2-activating protein [Source:ZFIN;Acc:ZDB-G
phospho1
phosphatidylinositol-3,4,5-trisphosphate-dependent Rac exc
phosphatidylinositol glycan anchor biosynthesis, class W [Sc
phosphatidylinositol glycan anchor biosynthesis, class B [Sou
phosphate regulating endopeptidase homolog, X-linked [Sou
PHD finger protein 12b [Source:ZFIN;Acc:ZDB-GENE-110628
peptidoglycan recognition protein 6 [Source:ZFIN;Acc:ZDB-G
peptidoglycan recognition protein 6 [Source:ZFIN;Acc:ZDB-G
PDZ and LIM domain 5b [Source:ZFIN;Acc:ZDB-GENE-040426
pcmt
pantothenate kinase 1a [Source:ZFIN;Acc:ZDB-GENE-040426
p53-induced death domain protein 1 [Source:ZFIN;Acc:ZDB-
oxysterol binding protein-like 6 [Source:ZFIN;Acc:ZDB-GENE
oxysterol binding protein-like 1A [Source:ZFIN;Acc:ZDB-GEN
otoferlin a [Source:ZFIN;Acc:ZDB-GENE-030131-7778]
Oryzias latipes RAD21 cohesin complex component (rad21),
Oryzias latipes fucosyltransferase 11 (fut11), mRNA. [Source

Oryzias latipes estrogen-related receptor gamma type 2 (err
origin recognition complex, subunit 3 [Source:ZFIN;Acc:ZDB-
origin recognition complex, subunit 2 [Source:ZFIN;Acc:ZDB-
opsin 5 [Source:ZFIN;Acc:ZDB-GENE-041001-179]
olfactory receptor C family, g6 [Source:ZFIN;Acc:ZDB-GENE-
nucleoporin 35 [Source:HGNC Symbol;Acc:HGNC:29797]
nucleoporin 214 [Source:ZFIN;Acc:ZDB-GENE-110203-3]
nuclear receptor coactivator 3 [Source:ZFIN;Acc:ZDB-GENE-
nuclear receptor coactivator 3 [Source:HGNC Symbol;Acc:H
ntrk2a
npepps
nmt2
NHL repeat containing 2 [Source:ZFIN;Acc:ZDB-GENE-10031
neuroguidin, EIF4E binding protein [Source:ZFIN;Acc:ZDB-GE
neuroblastoma amplified sequence [Source:ZFIN;Acc:ZDB-G
neurobeachin a [Source:ZFIN;Acc:ZDB-GENE-050320-68]
neuralized E3 ubiquitin protein ligase 4 [Source:ZFIN;Acc:ZD
neu4
Nedd4 family interacting protein 1 [Source:ZFIN;Acc:ZDB-GE
nedd4 binding protein 1 [Source:ZFIN;Acc:ZDB-GENE-03013
NCK-associated protein 1 [Source:ZFIN;Acc:ZDB-GENE-0301
naprt
N-acylsphingosine amidohydrolase 2 [Source:ZFIN;Acc:ZDB-
N-acetyl-alpha-glucosaminidase [Source:HGNC Symbol;Acc:
myosin, heavy chain 9a, non-muscle [Source:ZFIN;Acc:ZDB-
myosin XIX [Source:ZFIN;Acc:ZDB-GENE-140106-116]
myosin light chain kinase b [Source:ZFIN;Acc:ZDB-GENE-041
myocardial zonula adherens protein [Source:ZFIN;Acc:ZDB-
myo3a
myelin expression factor 2 [Source:ZFIN;Acc:ZDB-GENE-051
myelin expression factor 2 [Source:ZFIN;Acc:ZDB-GENE-051
muscle-specific beta 1 integrin binding protein [Source:ZFIN
multiple EGF-like-domains 8 [Source:ZFIN;Acc:ZDB-GENE-09
msra
Mov10 RISC complex RNA helicase like 1 [Source:HGNC Sym
MMS19 homolog, cytosolic iron-sulfur assembly component
mitoguardin 2 [Source:ZFIN;Acc:ZDB-GENE-050320-135]
mitogen-activated protein kinase 8 interacting protein 2 [So
mitochondrial fission process 1 [Source:HGNC Symbol;Acc:H
meningioma expressed antigen 5 (hyaluronidase) [Source:Z
membrane associated guanylate kinase, WW and PDZ doma
melanocortin 2 receptor accessory protein 2b [Source:ZFIN;
mediator complex subunit 12 [Source:ZFIN;Acc:ZDB-GENE-0
mark1
map2k1
major vault protein [Source:ZFIN;Acc:ZDB-GENE-030826-33]
lysine (K)-specific demethylase 5C [Source:ZFIN;Acc:ZDB-GE
low density lipoprotein receptor-related protein 1Bb [Sourc
lonp2
lin-9 DREAM MuvB core complex component [Source:ZFIN;
leucine-rich repeats and immunoglobulin-like domains 2 [Sc
leucine-rich repeat, immunoglobulin-like and transmembra

leucine rich repeat and fibronectin type III domain containir
laminin, alpha 5 [Source:ZFIN;Acc:ZDB-GENE-030131-9823]
laminin subunit alpha 2 [Source:HGNC Symbol;Acc:HGNC:64
kmt2d
kinesin family member 3A [Source:ZFIN;Acc:ZDB-GENE-0504
kinesin family member 18A [Source:ZFIN;Acc:ZDB-GENE-040
kinase non-catalytic C-lobe domain containing 1 [Source:HG
kelch-like family member 24a [Source:ZFIN;Acc:ZDB-GENE-0
kelch repeat and BTB (POZ) domain containing 8 [Source:ZFI
kelch domain containing 3 [Source:ZFIN;Acc:ZDB-GENE-0404
Josephin domain containing 2 [Source:ZFIN;Acc:ZDB-GENE-0
IQ motif and Sec7 domain 1b [Source:ZFIN;Acc:ZDB-GENE-1
intracisternal A particle-promoted polypeptide [Source:HGN
inter-alpha-trypsin inhibitor heavy chain family, member 5 [
integrin, beta 4 [Source:ZFIN;Acc:ZDB-GENE-030131-7209]
integrin, alpha 10 [Source:ZFIN;Acc:ZDB-GENE-100922-54]
integrin, alpha 1 [Source:ZFIN;Acc:ZDB-GENE-091118-115]
insulin induced gene 1 [Source:ZFIN;Acc:ZDB-GENE-030131-
HMBS
hemicentin 1 [Source:ZFIN;Acc:ZDB-GENE-041014-322]
helicase with zinc finger 2, transcriptional coactivator [Sourc
heat shock protein 12A [Source:ZFIN;Acc:ZDB-GENE-031001
harbinger transposase derived 1 [Source:ZFIN;Acc:ZDB-GEN
hadhab
hacd1
gucy2g
growth regulation by estrogen in breast cancer 1 [Source:ZF
growth arrest-specific 6 [Source:ZFIN;Acc:ZDB-GENE-03013
grk1
golgi reassembly stacking protein 2 [Source:HGNC Symbol;A
glyoxylate reductase/hydroxypyruvate reductase a [Source:
glycogenin 1a [Source:ZFIN;Acc:ZDB-GENE-040426-2910]
glutamic-oxaloacetic transaminase 2b, mitochondrial [Sourc
glutamate receptor, metabotropic 3 [Source:ZFIN;Acc:ZDB-
glutamate receptor, metabotropic 2b [Source:ZFIN;Acc:ZDB-
glutamate receptor, ionotropic, N-methyl D-aspartate 2A, b
GGT7
gba
G protein-coupled receptor 89B [Source:HGNC Symbol;Acc:
G protein-coupled receptor 137B [Source:HGNC Symbol;Acc
G protein signaling modulator 1b [Source:ZFIN;Acc:ZDB-GEN
frizzled class receptor 3b [Source:ZFIN;Acc:ZDB-GENE-07012
Fras1 related extracellular matrix 1b [Source:ZFIN;Acc:ZDB-
flt4
fkbp7
finTRIM family, member 83 [Source:ZFIN;Acc:ZDB-GENE-031
fibronectin type III and SPRY domain containing 1 [Source:ZI
fibrillin 1 [Source:HGNC Symbol;Acc:HGNC:3603]
fer-1-like family member 6 [Source:ZFIN;Acc:ZDB-GENE-070
fbp1a (1 of many)
FAT atypical cadherin 3a [Source:ZFIN;Acc:ZDB-GENE-06092
FAT atypical cadherin 3 [Source:HGNC Symbol;Acc:HGNC:23

FAT atypical cadherin 1a [Source:ZFIN;Acc:ZDB-GENE-05042
FAD-dependent oxidoreductase domain containing 2 [Sourc
F-box protein 46 [Source:HGNC Symbol;Acc:HGNC:25069]
F-box and WD repeat domain containing 9 [Source:ZFIN;Acc
extended synaptotagmin-like protein 1a [Source:ZFIN;Acc:ZI
exocyst complex component 6 [Source:ZFIN;Acc:ZDB-GENE-
estrogen receptor 2b [Source:ZFIN;Acc:ZDB-GENE-030116-1
estrogen receptor 2b [Source:ZFIN;Acc:ZDB-GENE-030116-1
estrogen receptor 2b [Source:ZFIN;Acc:ZDB-GENE-030116-1
erap2
EPHA4
ENPP2
endothelin converting enzyme-like 1 [Source:ZFIN;Acc:ZDB-
EF-hand and coiled-coil domain containing 1 [Source:ZFIN;A
ectonucleoside triphosphate diphosphohydrolase 4 [Source
ectonucleoside triphosphate diphosphohydrolase 3 [Source
echinoderm microtubule associated protein like 4 [Source:H
dynamin 1-like [Source:ZFIN;Acc:ZDB-GENE-040426-1556]
dpy-19 like 4 [Source:HGNC Symbol;Acc:HGNC:27829]
dpp3
Down syndrome cell adhesion molecule like 1 [Source:ZFIN;
double zinc ribbon and ankyrin repeat domains 1 [Source:ZF
dopey family member 2 [Source:ZFIN;Acc:ZDB-GENE-04042
dopey family member 1 [Source:ZFIN;Acc:ZDB-GENE-05030
DnaJ (Hsp40) homolog, subfamily C, member 15 [Source:ZFI
DnaJ (Hsp40) homolog, subfamily B, member 12b [Source:ZI
dnah11
DNA (cytosine-5-)-methyltransferase 3 beta, duplicate a [So
discs, large (Drosophila) homolog-associated protein 1a [Sou
dedicator of cytokinesis 5 [Source:ZFIN;Acc:ZDB-GENE-0801
DEAH (Asp-Glu-Ala-His) box polypeptide 33 [Source:ZFIN;Ac
DEAH (Asp-Glu-Ala-His) box helicase 30 [Source:ZFIN;Acc:ZD
DEAD (Asp-Glu-Ala-Asp) box polypeptide 52 [Source:ZFIN;Ac
damage-specific DNA binding protein 1 [Source:ZFIN;Acc:ZD
cytoplasmic FMR1 interacting protein 2 [Source:ZFIN;Acc:ZD
cytohesin 2 [Source:HGNC Symbol;Acc:HGNC:9502]
cytochrome P450, family 26, subfamily C, polypeptide 1 [Sou
cytochrome P450, family 2, subfamily N, polypeptide 13 [So
cytochrome P450, family 2, subfamily AE, polypeptide 1 [Sou
cysteine rich protein 2 [Source:HGNC Symbol;Acc:HGNC:23
cyclin T2a [Source:ZFIN;Acc:ZDB-GENE-030131-5779]
cyb561
CWF19-like 1, cell cycle control [Source:ZFIN;Acc:ZDB-GENE
cubilin (intrinsic factor-cobalamin receptor) [Source:ZFIN;Ac
CREBBP
CREBBP
corticotropin releasing hormone receptor 1 [Source:ZFIN;Ac
contactin associated protein 1 [Source:ZFIN;Acc:ZDB-GENE-
contactin associated protein 1 [Source:ZFIN;Acc:ZDB-GENE-
collagen, type V, alpha 3b [Source:ZFIN;Acc:ZDB-GENE-1107
collagen, type V, alpha 3a [Source:ZFIN;Acc:ZDB-GENE-0301
collagen, type IV, alpha 5 (Alport syndrome) [Source:ZFIN;Ac

collagen and calcium binding EGF domains 1 [Source:ZFIN;A
coiled-coil and C2 domain containing 1A [Source:HGNC Sym
CNKSR family member 3 [Source:HGNC Symbol;Acc:HGNC:2
claudin c [Source:ZFIN;Acc:ZDB-GENE-010328-3]
claudin 15a [Source:ZFIN;Acc:ZDB-GENE-040426-1305]
claudin 1 [Source:ZFIN;Acc:ZDB-GENE-010328-11]
chromodomain helicase DNA binding protein 6 [Source:ZFIN
chromodomain helicase DNA binding protein 1-like [Source:
chondroitin sulfate N-acetylgalactosaminyltransferase 1a [S
cholinergic receptor, nicotinic, epsilon [Source:ZFIN;Acc:ZDE
cholinergic receptor, nicotinic, alpha 1 (muscle) [Source:ZFIN
cholinergic receptor, muscarinic 5a [Source:ZFIN;Acc:ZDB-G
cerebral cavernous malformation 2-like [Source:ZFIN;Acc:ZD
Cdk5 and Abl enzyme substrate 2a [Source:ZFIN;Acc:ZDB-GE
cdk12
CDC5 cell division cycle 5-like (S. pombe) [Source:ZFIN;Acc:Z
CDC5 cell division cycle 5-like (S. pombe) [Source:ZFIN;Acc:Z
catenin (cadherin-associated protein), delta 2a [Source:ZFIN
carnitine palmitoyltransferase 1Cb [Source:ZFIN;Acc:ZDB-GE
carboxypeptidase O [Source:ZFIN;Acc:ZDB-GENE-070619-6]
carboxylesterase 3 [Source:ZFIN;Acc:ZDB-GENE-030131-956
CAP-GLY domain containing linker protein 1a [Source:ZFIN;A
camkk1a
calponin 3, acidic b [Source:ZFIN;Acc:ZDB-GENE-050522-263
calmodulin regulated spectrin-associated protein family, me
calcineurin-like phosphoesterase domain containing 1 [Sour
calcineurin-like phosphoesterase domain containing 1 [Sour
cadherin, EGF LAG seven-pass G-type receptor 2 [Source:ZFI
cadherin 7 [Source:HGNC Symbol;Acc:HGNC:1766]
cadherin 4, type 1, R-cadherin (retinal) [Source:ZFIN;Acc:ZD
C1q and TNF related 4 [Source:ZFIN;Acc:ZDB-GENE-050417-
BTB (POZ) domain containing 7 [Source:ZFIN;Acc:ZDB-GENE-
bsk146
bromodomain and WD repeat domain containing 3 [Source:
bridging integrator 1b [Source:ZFIN;Acc:ZDB-GENE-030425-
bpgm
bpgm
bobby sox homolog (Drosophila) [Source:ZFIN;Acc:ZDB-GEN
basic leucine zipper nuclear factor 1 [Source:HGNC Symbol;
autophagy/beclin-1 regulator 1b [Source:ZFIN;Acc:ZDB-GEN
ATPase phospholipid transporting 8B4 [Source:ZFIN;Acc:ZDE
ATPase copper transporting alpha [Source:ZFIN;Acc:ZDB-GE
atp2a2a
ATP11A (1 of many)
ATP-binding cassette, sub-family C (CFTR/MRP), member 3 [
ataxin 7 [Source:ZFIN;Acc:ZDB-GENE-110621-1]
ataxin 10 [Source:ZFIN;Acc:ZDB-GENE-060503-180]
aspartylglucosaminidase [Source:ZFIN;Acc:ZDB-GENE-04042
asparaginase like 1 [Source:ZFIN;Acc:ZDB-GENE-050320-102
arylsulfatase H [Source:ZFIN;Acc:ZDB-GENE-081104-120]
aryl hydrocarbon receptor nuclear translocator-like 2 [Sourc
armadillo repeat containing 1 [Source:ZFIN;Acc:ZDB-GENE-0

arih2
ArfGAP with SH3 domain, ankyrin repeat and PH domain 3 [
ArfGAP with RhoGAP domain, ankyrin repeat and PH domai
aquarius intron-binding spliceosomal factor [Source:ZFIN;Ac
anoctamin 10a [Source:ZFIN;Acc:ZDB-GENE-050913-43]
anoctamin 10a [Source:ZFIN;Acc:ZDB-GENE-050913-43]
aldh18a1
aldehyde dehydrogenase 9 family, member A1b [Source:ZFI
ADP-ribosylation factor-like 8Bb [Source:ZFIN;Acc:ZDB-GENI
ADP-ribosylation factor-like 8Ba [Source:ZFIN;Acc:ZDB-GENI
adhesion G protein-coupled receptor V1 [Source:ZFIN;Acc:Z
adenomatosis polyposis coli 2 [Source:ZFIN;Acc:ZDB-GENE-(
adaptor-related protein complex 2, mu 1 subunit, b [Source
adaptor-related protein complex 1, gamma 1 subunit [Sourc
adaptor protein, phosphotyrosine interaction, PH domain ai
ADAM metallopeptidase domain 32 [Source:HGNC Symbol;/
ADAM metallopeptidase domain 22 [Source:ZFIN;Acc:ZDB-G
ADAM metallopeptidase domain 17 [Source:HGNC Symbol;/
aconitate decarboxylase 1 [Source:ZFIN;Acc:ZDB-GENE-0811
acadsb
acadl
ABI family, member 3 (NESH) binding protein a [Source:ZFIN
abhydrolase domain containing 16A [Source:ZFIN;Acc:ZDB-(
abcb11a
abca4a
aatka
aak1a
A kinase (PRKA) anchor protein 11 [Source:ZFIN;Acc:ZDB-GE
3-hydroxyisobutyryl-CoA hydrolase [Source:ZFIN;Acc:ZDB-G
mars-02
ribosomal protein S3 [Source:ZFIN;Acc:ZDB-GENE-030131-8
DnaJ (Hsp40) homolog, subfamily C, member 11a [Source:ZI

atp2a2a
atp2a2a
abcb11a
cp
ENPP2
erap2
gcnt7
loxhd1a
mocs3
PLCZ1
prkaa1
ppa1a
tyr
dystrophin related protein 2 [Source:ZFIN;Acc:ZDB-GENE-07

caspase recruitment domain family, member 14 [Source:ZFI
transforming growth factor, beta 2, like [Source:ZFIN;Acc:ZD
transforming growth factor, beta 2, like [Source:ZFIN;Acc:ZD
collagen type XVII alpha 1 chain [Source:HGNC Symbol;Acc:H
DAN domain family, member 5 [Source:ZFIN;Acc:ZDB-GENE-
tromal interaction molecule 2a [Source:ZFIN;Acc:ZDB-GENE-
ADP-ribosylation factor-like 3, like 2 [Source:ZFIN;Acc:ZDB-G
ATPase copper transporting alpha [Source:ZFIN;Acc:ZDB-GE
ATPase copper transporting alpha [Source:ZFIN;Acc:ZDB-GE
cytochrome P450 family 2 subfamily C member 8 [Source:H
zmp:0000000846 [Source:ZFIN;Acc:ZDB-GENE-130530-849]
RAB30, member RAS oncogene family [Source:ZFIN;Acc:ZDB
Fras1 related extracellular matrix protein 2b [Source:ZFIN;A
Fras1 related extracellular matrix protein 2b [Source:ZFIN;A
SLIT and NTRK-like family, member 4 [Source:ZFIN;Acc:ZDB-
fibrinogen-like 1 [Source:ZFIN;Acc:ZDB-GENE-130815-1]
procollagen, type IX, alpha 2 [Source:ZFIN;Acc:ZDB-GENE-03
si:ch211-229c8.4 [Source:ZFIN;Acc:ZDB-GENE-050419-129]
si:dkey-166k12.1 [Source:ZFIN;Acc:ZDB-GENE-120215-78]
si:dkey-166k12.1 [Source:ZFIN;Acc:ZDB-GENE-120215-78]
si:ch73-125k17.2 [Source:ZFIN;Acc:ZDB-GENE-120215-164]
collagen and calcium binding EGF domains 1 [Source:ZFIN;A
solute carrier family 2 (facilitated glucose transporter), mem
uracil phosphoribosyltransferase (FUR1) homolog (S. cerevis
intermediate filament family orphan 1a [Source:ZFIN;Acc:ZD
G protein signaling modulator 1b [Source:ZFIN;Acc:ZDB-GEN

solute carrier family 46 (folate transporter), member 1 [Sou
pyrimidinergic receptor P2Y6 [Source:HGNC Symbol;Acc:HG
dopey family member 1 [Source:ZFIN;Acc:ZDB-GENE-05030
early B cell factor 1 [Source:HGNC Symbol;Acc:HGNC:3126]
dendrocyte expressed seven transmembrane protein [Sourc
claudin h [Source:ZFIN;Acc:ZDB-GENE-010328-8]
claudin h [Source:ZFIN;Acc:ZDB-GENE-010328-8]
echinoderm microtubule associated protein like 4 [Source:H
pantothenate kinase 1a [Source:ZFIN;Acc:ZDB-GENE-04042
pantothenate kinase 1a [Source:ZFIN;Acc:ZDB-GENE-04042
solute carrier family 22 (organic anion transporter), membe
calcineurin-like phosphoesterase domain containing 1 [Sour
neuralized E3 ubiquitin protein ligase 2 [Source:ZFIN;Acc:ZD
solute carrier family 2 member 4 [Source:HGNC Symbol;Acc
GDNF family receptor alpha 3 [Source:ZFIN;Acc:ZDB-GENE-1
leucine-rich repeat, immunoglobulin-like and transmembra
si:ch211-284e13.4 [Source:ZFIN;Acc:ZDB-GENE-060526-161
meningioma expressed antigen 5 (hyaluronidase) [Source:Z
meningioma expressed antigen 5 (hyaluronidase) [Source:Z

APC membrane recruitment protein 2 [Source:ZFIN;Acc:ZDB
claudin 15a [Source:ZFIN;Acc:ZDB-GENE-040426-1305]
si:ch211-217a12.1 [Source:ZFIN;Acc:ZDB-GENE-121214-95]
5-methyltetrahydrofolate-homocysteine methyltransferase
XK, Kell blood group complex subunit-related family, memb

endothelin receptor type A [Source:HGNC Symbol;Acc:HGN
neuroligin 2a [Source:ZFIN;Acc:ZDB-GENE-090918-2]
short chain dehydrogenase/reductase family 16C, member
ectonucleoside triphosphate diphosphohydrolase 3 [Source
Oryzias latipes RAD21 cohesin complex component (rad21),
carboxypeptidase X, M14 family member 2 [Source:HGNC S
transient receptor potential cation channel, subfamily C, me
si:ch73-63e15.2 [Source:ZFIN;Acc:ZDB-GENE-091204-463]
myelin expression factor 2 [Source:ZFIN;Acc:ZDB-GENE-051
ATPase phospholipid transporting 8B2 [Source:HGNC Symbo
basic leucine zipper nuclear factor 1 [Source:HGNC Symbol;
NTPase, KAP family P-loop domain containing 1 [Source:ZFI
adaptor-related protein complex 1, gamma 1 subunit [Sourc
myocardial zonula adherens protein [Source:ZFIN;Acc:ZDB-
kinesin family member 3A [Source:ZFIN;Acc:ZDB-GENE-050
feline leukemia virus subgroup C cellular receptor family, m
Oryzias latipes 50 kD glycoprotein (rh50), mRNA. [Source:Re
opsin 8, group member b [Source:ZFIN;Acc:ZDB-GENE-0707
T-box 18 [Source:ZFIN;Acc:ZDB-GENE-020529-2]

cadherin, EGF LAG seven-pass G-type receptor 2 [Source:ZF
bassoon (presynaptic cytomatrix protein) b [Source:ZFIN;Ac
Fras1 related extracellular matrix 1b [Source:ZFIN;Acc:ZDB-
fibrillin 2b [Source:ZFIN;Acc:ZDB-GENE-090112-3]
R3H domain containing 2 [Source:HGNC Symbol;Acc:HGNC:
solute carrier family 5 (iodide transporter), member 8-like [
SWI/SNF related, matrix associated, actin dependent regula
family with sequence similarity 43, member A [Source:ZFIN;
EGF domain-specific O-linked N-acetylglucosamine (GlcNAc)
si:ch73-61d6.3 [Source:ZFIN;Acc:ZDB-GENE-091204-109]
cysteine rich protein 2 [Source:HGNC Symbol;Acc:HGNC:23
kazrin, periplakin interacting protein [Source:HGNC Symbol;
calcium channel, voltage-dependent, L type, alpha 1S subun
oxysterol binding protein-like 2b [Source:ZFIN;Acc:ZDB-GEN
SMAD family member 3b [Source:ZFIN;Acc:ZDB-GENE-0301
calcium channel, voltage-dependent, L type, alpha 1S subun
calcium channel, voltage-dependent, L type, alpha 1S subun
transient receptor potential cation channel, subfamily M, m
transient receptor potential cation channel, subfamily M, m
transient receptor potential cation channel, subfamily M, m

si:dkeyp-59c12.1 [Source:ZFIN;Acc:ZDB-GENE-050419-167]
solute carrier family 6 (neurotransmitter transporter), mem
cholinergic receptor, muscarinic 2b [Source:ZFIN;Acc:ZDB-G
cholinergic receptor, muscarinic 2b [Source:ZFIN;Acc:ZDB-G

si:ch211-214j8.1 [Source:ZFIN;Acc:ZDB-GENE-060526-100]
dedicator of cytokinesis 8 [Source:ZFIN;Acc:ZDB-GENE-0605
apoptosis-inducing factor, mitochondrion-associated, 3 [Sou
transient receptor potential cation channel, subfamily M, m
transient receptor potential cation channel, subfamily M, m
spinster homolog 3 (Drosophila) [Source:ZFIN;Acc:ZDB-GENI
spinster homolog 3 (Drosophila) [Source:ZFIN;Acc:ZDB-GENI
solute carrier family 2 (facilitated glucose transporter), mem
transmembrane channel-like 8 [Source:ZFIN;Acc:ZDB-GENE-
G protein-coupled receptor 137B [Source:HGNC Symbol;Acc
uroporphyrinogen decarboxylase [Source:ZFIN;Acc:ZDB-GEN
kelch domain containing 3 [Source:ZFIN;Acc:ZDB-GENE-040
kelch domain containing 3 [Source:ZFIN;Acc:ZDB-GENE-040
kelch domain containing 3 [Source:ZFIN;Acc:ZDB-GENE-040
SH3-domain GRB2-like endophilin B2b [Source:ZFIN;Acc:ZDE
prostaglandin E receptor 1a (subtype EP1) [Source:ZFIN;Acc
5-hydroxytryptamine (serotonin) receptor 3A [Source:ZFIN;
SH3-domain binding protein 2 [Source:ZFIN;Acc:ZDB-GENE-
heart and neural crest derivatives expressed 1 [Source:HGN
glycogenin 1a [Source:ZFIN;Acc:ZDB-GENE-040426-2910]
glycogenin 1a [Source:ZFIN;Acc:ZDB-GENE-040426-2910]
cadherin 7 [Source:HGNC Symbol;Acc:HGNC:1766]
uroporphyrinogen decarboxylase [Source:ZFIN;Acc:ZDB-GEN
abhydrolase domain containing 16A [Source:ZFIN;Acc:ZDB-G
otolith matrix protein [Source:ZFIN;Acc:ZDB-GENE-040709-1
adaptor-related protein complex 2, mu 1 subunit, b [Source
mesenchyme homeobox 1 [Source:ZFIN;Acc:ZDB-GENE-040
chromatin accessibility complex 1 [Source:HGNC Symbol;Ac
protein phosphatase 2 regulatory subunit B'gamma [Source
tensin 1b [Source:ZFIN;Acc:ZDB-GENE-030131-6933]
glycoprotein hormones, alpha polypeptide [Source:ZFIN;Acc
3-hydroxyisobutyryl-CoA hydrolase [Source:ZFIN;Acc:ZDB-G
bridging integrator 1a [Source:ZFIN;Acc:ZDB-GENE-070912-
arylsulfatase H [Source:ZFIN;Acc:ZDB-GENE-081104-120]
solute carrier family 35, member F4 [Source:ZFIN;Acc:ZDB-G

potassium channel, subfamily K, member 3a [Source:ZFIN;A
lin-9 DREAM MuvB core complex component [Source:ZFIN;
spalt-like transcription factor 1a [Source:ZFIN;Acc:ZDB-GEN
utrophin [Source:ZFIN;Acc:ZDB-GENE-070228-6]
solute carrier family 38, member 11 [Source:ZFIN;Acc:ZDB-
connexin 35.4 [Source:ZFIN;Acc:ZDB-GENE-050417-174]
G protein-coupled receptor 89B [Source:HGNC Symbol;Acc:
IQ motif and Sec7 domain 1b [Source:ZFIN;Acc:ZDB-GENE-1
IQ motif and Sec7 domain 1b [Source:ZFIN;Acc:ZDB-GENE-1
TGF-beta activated kinase 1/MAP3K7 binding protein 2 [Sou
myosin, heavy chain 6, cardiac muscle, alpha [Source:ZFIN;A
myosin binding protein C, slow type [Source:ZFIN;Acc:ZDB-
SLIT-ROBO Rho GTPase activating protein 1b [Source:ZFIN;A
leucine rich repeat and fibronectin type III domain containir
RAB37, member RAS oncogene family [Source:HGNC Symbo
solute carrier family 16, member 7 (monocarboxylic acid tra
RAP1 GTPase activating protein [Source:ZFIN;Acc:ZDB-GENE
tight junction protein 3 [Source:ZFIN;Acc:ZDB-GENE-030828
XK, Kell blood group complex subunit-related family, memb
AHA1, activator of heat shock protein ATPase homolog 1b [

dedicator of cytokinesis 5 [Source:ZFIN;Acc:ZDB-GENE-0801
SMAD family member 2 [Source:ZFIN;Acc:ZDB-GENE-99060

carnitine palmitoyltransferase 1Cb [Source:ZFIN;Acc:ZDB-GE
carnitine palmitoyltransferase 1Cb [Source:ZFIN;Acc:ZDB-GE
muscle-specific beta 1 integrin binding protein [Source:ZFIN
peripherin 2a (retinal degeneration, slow) [Source:ZFIN;Acc:
regucalcin [Source:ZFIN;Acc:ZDB-GENE-040718-68]
tRNA aspartic acid methyltransferase 1 [Source:ZFIN;Acc:ZD
aconitase 1, soluble [Source:ZFIN;Acc:ZDB-GENE-031118-76
sodium voltage-gated channel alpha subunit 4 [Source:HGN

Gene description	Expressed in ovary
siae) [Source:ZFIN;Acc:ZDB-GENE-040426-2411]	yes
N;Acc:ZDB-GENE-061103-619]	yes
l;Acc:HGNC:30232]	yes
	yes
-2572]	yes
B-GENE-040801-185]	yes
putative pyruvate dehydrogenase phosphatase isoenzyme 2 [Source:ZFIN	yes
E-030131-6138]	yes
5'-nucleotidase, cytosolic IIIB [Source:HGNC Symbol;Acc:HGNC:28300]	yes
inositol-tetrakisphosphate 1-kinase b [Source:ZFIN;Acc:ZDB-GENE-131126	yes
ZDB-GENE-040426-1975]	yes
ZDB-GENE-040426-1975]	yes
dual specificity phosphatase 1 [Source:ZFIN;Acc:ZDB-GENE-040426-2018]	yes
DB-GENE-070628-3]	yes
	yes
ENE-030131-6514]	yes
618-2]	yes
protein kinase, AMP-activated, alpha 1 catalytic subunit [Source:ZFIN;Acc:	yes
	yes
on 3 (stat3), mRNA. [Source:RefSeq mRNA;Acc:NM_001104838]	yes
]	yes
E-050522-67]	yes
[Source:ZFIN;Acc:ZDB-GENE-081104-155]	yes
ce:ZFIN;Acc:ZDB-GENE-040614-3]	yes
FK506 binding protein 1Ab [Source:ZFIN;Acc:ZDB-GENE-040927-9]	yes
FK506 binding protein 1Aa [Source:ZFIN;Acc:ZDB-GENE-030131-7275]	yes
B-GENE-030131-1565]	yes
408-2]	yes
ce:HGNC Symbol;Acc:HGNC:1490]	yes
Source:ZFIN;Acc:ZDB-GENE-030131-664]	yes
adenylate cyclase 7 [Source:ZFIN;Acc:ZDB-GENE-040713-1]	yes
FIN;Acc:ZDB-GENE-060503-816]	yes
]	yes
	yes
-49]	yes
-9613]	yes
4-18]	yes
-3]	yes
-7577]	yes
ENE-050419-13]	yes
bol;Acc:HGNC:23836]	yes
rce:ZFIN;Acc:ZDB-GENE-000208-4]	yes
	yes
	yes
	yes
	yes
FIN;Acc:ZDB-GENE-040426-948]	yes
118-93]	yes
ymbol;Acc:HGNC:12762]	yes
WNK lysine deficient protein kinase 4 [Source:HGNC Symbol;Acc:HGNC:1	yes
ource:ZFIN;Acc:ZDB-GENE-081104-419]	yes

;-3]	yes
:ZDB-GENE-070705-153]	yes
:ZDB-GENE-041024-4]	yes
\cc:ZDB-GENE-100302-1]	yes
-GENE-040426-803]	yes
131-5408]	yes
	yes
ubiquitin specific peptidase 16 [Source:ZFIN;Acc:ZDB-GENE-030131-4153]	yes
NE-000208-18]	yes
40426-907]	yes
N;Acc:ZDB-GENE-050517-21]	yes
urce:ZFIN;Acc:ZDB-GENE-030131-5971]	yes
803-1]	yes
77]	yes
N;Acc:ZDB-GENE-050320-73]	yes
ZFIN;Acc:ZDB-GENE-040426-953]	yes
)B-GENE-041008-138]	yes
)B-GENE-041008-138]	yes
40724-145]	yes
thyroid hormone receptor interactor 12 [Source:ZFIN;Acc:ZDB-GENE-0411	yes
NE-030131-8404]	yes
thyrotropin releasing hormone degrading enzyme, tandem duplicate 2 [So	yes
	yes
ZDB-GENE-040625-140]	yes
ıember 3 [Source:ZFIN;Acc:ZDB-GENE-060531-95]	yes
ıember 1b [Source:ZFIN;Acc:ZDB-GENE-070424-31]	yes
ember 1 [Source:ZFIN;Acc:ZDB-GENE-070830-1]	yes
ember 1b [Source:ZFIN;Acc:ZDB-GENE-050106-1]	yes
	yes
)B-GENE-130425-3]	yes
1-9012]	yes
25-1]	yes
30131-9745]	yes
	yes
ıcc:HGNC:29944]	yes
IE-030131-2064]	yes
sociated factor [Source:ZFIN;Acc:ZDB-GENE-030131-5576]	yes
	yes
spleen tyrosine kinase [Source:ZFIN;Acc:ZDB-GENE-040702-3]	yes
tor of chromatin, subfamily d, member 1 [Source:ZFIN;Acc:ZDB-GENE-030	yes
B-GENE-100913-1]	yes
IN;Acc:ZDB-GENE-050417-228]	yes
c:ZDB-GENE-030131-105]	yes
26-1198]	yes
Acc:ZDB-GENE-031106-3]	yes
3-GENE-080725-1]	yes
3b [Source:ZFIN;Acc:ZDB-GENE-121226-2]	yes
E-060531-7]	yes
:26992]	yes
GENE-060929-204]	yes
)906-1]	yes
E-020228-2]	yes

	yes
1 [Source:ZFIN;Acc:ZDB-GENE-041210-50]	yes
ystem), member 3a [Source:ZFIN;Acc:ZDB-GENE-041114-206]	yes
ber 11b [Source:ZFIN;Acc:ZDB-GENE-121116-2]	yes
:c:HGNC:13811]	yes
GENE-050417-122]	yes
GENE-041210-186]	yes
e:ZFIN;Acc:ZDB-GENE-041014-347]	yes
[Source:ZFIN;Acc:ZDB-GENE-050706-104]	yes
:HGNC:11009]	yes
nber 2 [Source:ZFIN;Acc:ZDB-GENE-030131-1213]	yes
nsporter 2) [Source:ZFIN;Acc:ZDB-GENE-051113-248]	yes
N;Acc:ZDB-GENE-040718-457]	yes
28-4]	yes
3-7]	yes
0306-4]	yes
DB-GENE-040426-2801]	yes
J;Acc:ZDB-GENE-050420-109]	yes
FIN;Acc:ZDB-GENE-030616-264]	yes
	yes
	yes
	yes
si:dkey-160o24.3 [Source:ZFIN;Acc:ZDB-GENE-140106-224]	yes
nicotinamide nucleotide adenylyltransferase 3 [Source:HGNC Symbol;Acc:	yes
	yes
	yes
6]	yes
	yes
]	yes
]	yes
7]	yes
5a [Source:ZFIN;Acc:ZDB-GENE-040426-2049]	yes
5a [Source:ZFIN;Acc:ZDB-GENE-040426-2049]	yes
selenophosphate synthetase 1 [Source:ZFIN;Acc:ZDB-GENE-030131-3670]	yes
nic domain, (semaphorin) 6Ba [Source:ZFIN;Acc:ZDB-GENE-131024-1]	yes
NE-040426-2784]	yes
35]	yes
E-061226-3]	yes
RNA guanylyltransferase and 5'-phosphatase [Source:ZFIN;Acc:ZDB-GENE-	yes
40]	yes
-2]	yes
0788]	yes
ENE-120516-2]	yes
ENE-120516-2]	yes
ENE-080220-26]	yes
0426-2595]	yes
REV3-like, polymerase (DNA directed), zeta, catalytic subunit [Source:ZFIN	yes
526-436]	yes
1]	yes
	yes
	yes
nbol;Acc:HGNC:13232]	yes

ENE-061013-179]	yes
Source:ZFIN;Acc:ZDB-GENE-130530-945]	yes
urce:ZFIN;Acc:ZDB-GENE-131122-56]	yes
l;Acc:HGNC:9776]	yes
B-GENE-040718-250]	yes
DB-GENE-050706-113]	yes
B-GENE-030826-20]	yes
B-GENE-030826-20]	yes
B-GENE-040718-304]	yes
DB-GENE-030131-6682]	yes
DB-GENE-030131-6682]	yes
29167]	yes
NC:8543]	yes
prostaglandin reductase 2 [Source:ZFIN;Acc:ZDB-GENE-050506-71]	yes
pre-mRNA processing factor 4Bb [Source:ZFIN;Acc:ZDB-GENE-040426-279	yes
:HGNC Symbol;Acc:HGNC:9311]	yes
030131-3595]	yes
e:ZFIN;Acc:ZDB-GENE-030131-9008]	yes
0415-216]	yes
0415-216]	yes
GENE-030131-8730]	yes
6-397]	yes
03-1]	yes
03-1]	yes
peptidylprolyl isomerase Fb [Source:ZFIN;Acc:ZDB-GENE-051030-126]	yes
peptidylprolyl isomerase C [Source:ZFIN;Acc:ZDB-GENE-170217-1]	yes
member 7 [Source:ZFIN;Acc:ZDB-GENE-120214-39]	yes
Source:ZFIN;Acc:ZDB-GENE-040718-14]	yes
	yes
polymerase (DNA directed), theta [Source:ZFIN;Acc:ZDB-GENE-040724-23	yes
	yes
polycystic kidney disease 1 like 2a [Source:ZFIN;Acc:ZDB-GENE-050208-89	yes
	yes
) [Source:ZFIN;Acc:ZDB-GENE-040426-2079]	yes
GENE-030826-13]	yes
phosphatase, orphan 1 [Source:ZFIN;Acc:ZDB-GENE-040801-198]	yes
change factor 2 [Source:ZFIN;Acc:ZDB-GENE-091113-37]	yes
urce:ZFIN;Acc:ZDB-GENE-080204-94]	yes
urce:ZFIN;Acc:ZDB-GENE-040426-1216]	yes
urce:ZFIN;Acc:ZDB-GENE-030103-2]	yes
-2]	yes
GENE-071227-2]	yes
GENE-071227-2]	yes
6-908]	yes
protein-L-isoaspartate (D-aspartate) O-methyltransferase [Source:ZFIN;Ac	yes
6-2846]	yes
GENE-081104-353]	yes
-070912-206]	yes
IE-050208-657]	yes
	yes
mRNA. [Source:RefSeq mRNA;Acc:NM_001104691]	yes
e:RefSeq mRNA;Acc:NM_001104777]	yes

rg2), mRNA. [Source:RefSeq mRNA;Acc:NM_001163092]	yes
-GENE-030131-6247]	yes
-GENE-061013-682]	yes
	yes
081029-9]	yes
	yes
	yes
051107-8]	yes
GNC:7670]	yes
neurotrophic tyrosine kinase, receptor, type 2a [Source:ZFIN;Acc:ZDB-GEN	yes
aminopeptidase puromycin sensitive [Source:ZFIN;Acc:ZDB-GENE-060524	yes
N-myristoyltransferase 2 [Source:ZFIN;Acc:ZDB-GENE-030131-717]	yes
8-2]	yes
ENE-031030-13]	yes
ENE-041014-367]	yes
	yes
B-GENE-100318-4]	yes
Oryzias latipes sialidase 4 (neu4), mRNA. [Source:RefSeq mRNA;Acc:NM_(	yes
ENE-030131-2767]	yes
1-5538]	yes
31-4426]	yes
nicotinate phosphoribosyltransferase [Source:ZFIN;Acc:ZDB-GENE-04042	yes
GENE-041112-1]	yes
HGNC:7632]	yes
GENE-030131-5870]	yes
	yes
111-150]	yes
GENE-041111-91]	yes
myosin IIIA [Source:ZFIN;Acc:ZDB-GENE-041026-4]	yes
120-114]	yes
120-114]	yes
;Acc:ZDB-GENE-030404-1]	yes
0730-1]	yes
methionine sulfoxide reductase A [Source:ZFIN;Acc:ZDB-GENE-041014-34	yes
nbol;Acc:HGNC:7201]	yes
t [Source:ZFIN;Acc:ZDB-GENE-120316-2]	yes
	yes
urce:ZFIN;Acc:ZDB-GENE-070705-326]	yes
HGNC:26945]	yes
FIN;Acc:ZDB-GENE-030131-3208]	yes
ain containing 1b [Source:ZFIN;Acc:ZDB-GENE-030131-5110]	yes
Acc:ZDB-GENE-100729-5]	yes
60125-1]	yes
MAP/microtubule affinity-regulating kinase 1 [Source:ZFIN;Acc:ZDB-GENE	yes
mitogen-activated protein kinase kinase 1 [Source:ZFIN;Acc:ZDB-GENE-04	yes
]	yes
NE-060810-94]	yes
e:ZFIN;Acc:ZDB-GENE-070912-401]	yes
lon peptidase 2, peroxisomal [Source:ZFIN;Acc:ZDB-GENE-041212-1]	yes
Acc:ZDB-GENE-030131-1747]	yes
urce:ZFIN;Acc:ZDB-GENE-041111-75]	yes
ne domains 2 [Source:ZFIN;Acc:ZDB-GENE-050522-160]	yes

ng 2 [Source:HGNC Symbol;Acc:HGNC:21226]	yes
	yes
482]	yes
lysine (K)-specific methyltransferase 2D [Source:ZFIN;Acc:ZDB-GENE-0602	yes
417-71]	yes
0426-862]	yes
GNC Symbol;Acc:HGNC:29374]	yes
061013-572]	yes
IN;Acc:ZDB-GENE-030114-8]	yes
426-2426]	yes
040426-850]	yes
20813-1]	yes
NC Symbol;Acc:HGNC:6108]	yes
[Source:ZFIN;Acc:ZDB-GENE-041210-193]	yes
	yes
	yes
	yes
-6121]	yes
hydroxymethylbilane synthase [Source:HGNC Symbol;Acc:HGNC:4982]	yes
	yes
ce:ZFIN;Acc:ZDB-GENE-120920-1]	yes
-1]	yes
E-040608-1]	yes
hydroxyacyl-CoA dehydrogenase trifunctional multienzyme complex subu	yes
3-hydroxyacyl-CoA dehydratase 1 [Source:ZFIN;Acc:ZDB-GENE-170413-2]	yes
guanylate cyclase 2g [Source:ZFIN;Acc:ZDB-GENE-130531-70]	yes
FIN;Acc:ZDB-GENE-070112-332]	yes
1-7773]	yes
Oryzias latipes G protein-coupled receptor kinase 1 (grk1), mRNA. [Source	yes
Acc:HGNC:17500]	yes
ZFIN;Acc:ZDB-GENE-040724-230]	yes
	yes
ce:ZFIN;Acc:ZDB-GENE-030131-7917]	yes
GENE-061009-13]	yes
-GENE-060201-5]	yes
[Source:ZFIN;Acc:ZDB-GENE-070424-223]	yes
gamma-glutamyltransferase 7 [Source:HGNC Symbol;Acc:HGNC:4259]	yes
glucosidase, beta, acid [Source:ZFIN;Acc:ZDB-GENE-100922-270]	yes
HGNC:13840]	yes
c:HGNC:11862]	yes
NE-110203-5]	yes
22-1]	yes
GENE-050208-783]	yes
fms-related tyrosine kinase 4 [Source:ZFIN;Acc:ZDB-GENE-980526-326]	yes
FK506 binding protein 7 [Source:ZFIN;Acc:ZDB-GENE-030616-182]	yes
1002-11]	yes
FIN;Acc:ZDB-GENE-060503-218]	yes
	yes
0912-312]	yes
fructose-1,6-bisphosphatase 1a [Source:ZFIN;Acc:ZDB-GENE-030131-7171	yes
29-1254]	yes
3112]	yes

!5-1]	yes
:e:ZFIN;Acc:ZDB-GENE-060929-1026]	yes
	yes
:::ZDB-GENE-091204-198]	yes
DB-GENE-090311-55]	yes
040426-2511]	yes
.]	yes
.]	yes
.]	yes
endoplasmic reticulum aminopeptidase 2 [Source:ZFIN;Acc:ZDB-GENE-08(	yes
EPH receptor A4 [Source:HGNC Symbol;Acc:HGNC:3388]	yes
ectonucleotide pyrophosphatase/phosphodiesterase 2 [Source:HGNC Syn	yes
GENE-130530-692]	yes
.cc:ZDB-GENE-120813-3]	yes
:ZFIN;Acc:ZDB-GENE-040718-116]	yes
:ZFIN;Acc:ZDB-GENE-030131-6186]	yes
IGNC Symbol;Acc:HGNC:1316]	yes
	yes
	yes
dipeptidyl-peptidase 3 [Source:ZFIN;Acc:ZDB-GENE-030131-1247]	yes
Acc:ZDB-GENE-110601-1]	yes
FIN;Acc:ZDB-GENE-051113-296]	yes
6-1195]	yes
9-65]	yes
IN;Acc:ZDB-GENE-040801-216]	yes
FIN;Acc:ZDB-GENE-070410-128]	yes
dynein, axonemal, heavy chain 11 [Source:ZFIN;Acc:ZDB-GENE-100922-8]	yes
urce:ZFIN;Acc:ZDB-GENE-050314-4]	yes
urce:ZFIN;Acc:ZDB-GENE-030616-580]	yes
.08-4]	yes
:c:ZDB-GENE-131127-77]	yes
)B-GENE-130530-833]	yes
cc:ZDB-GENE-060623-1]	yes
)B-GENE-040426-1272]	yes
)B-GENE-080724-2]	yes
	yes
urce:ZFIN;Acc:ZDB-GENE-050714-2]	yes
urce:ZFIN;Acc:ZDB-GENE-041001-158]	yes
urce:ZFIN;Acc:ZDB-GENE-090312-107]	yes
51]	yes
	yes
cytochrome b561 [Source:ZFIN;Acc:ZDB-GENE-070713-3]	yes
-031204-5]	yes
:c:ZDB-GENE-060228-6]	yes
CREB binding protein [Source:HGNC Symbol;Acc:HGNC:2348]	yes
CREB binding protein [Source:HGNC Symbol;Acc:HGNC:2348]	yes
:c:ZDB-GENE-081231-1]	yes
030916-2]	yes
030916-2]	yes
'28-4]	yes
.31-968]	yes
cc:ZDB-GENE-030131-2281]	yes

cc:ZDB-GENE-090506-7]	yes
bol;Acc:HGNC:30237]	yes
3034]	yes
	yes
	yes
	yes
;Acc:ZDB-GENE-130530-559]	yes
:ZFIN;Acc:ZDB-GENE-040426-892]	yes
ource:ZFIN;Acc:ZDB-GENE-121129-1]	yes
3-GENE-040808-32]	yes
N;Acc:ZDB-GENE-980526-137]	yes
ENE-080723-32]	yes
DB-GENE-090313-248]	yes
ENE-091118-58]	yes
cyclin-dependent kinase 12 [Source:ZFIN;Acc:ZDB-GENE-081104-294]	yes
ZDB-GENE-040426-821]	yes
ZDB-GENE-040426-821]	yes
;Acc:ZDB-GENE-030616-60]	yes
ENE-050417-155]	yes
	yes
3]	yes
Acc:ZDB-GENE-060526-282]	yes
calcium/calmodulin-dependent protein kinase kinase 1, alpha a [Source:Z	yes
3]	yes
ember 3 [Source:ZFIN;Acc:ZDB-GENE-060503-811]	yes
rce:ZFIN;Acc:ZDB-GENE-041114-118]	yes
rce:ZFIN;Acc:ZDB-GENE-041114-118]	yes
IN;Acc:ZDB-GENE-070122-2]	yes
	yes
B-GENE-991207-1]	yes
-198]	yes
-030131-4477]	yes
brain specific kinase 146 [Source:ZFIN;Acc:ZDB-GENE-060313-3]	yes
:ZFIN;Acc:ZDB-GENE-100921-43]	yes
1]	yes
2,3-bisphosphoglycerate mutase [Source:ZFIN;Acc:ZDB-GENE-040718-375	yes
2,3-bisphosphoglycerate mutase [Source:ZFIN;Acc:ZDB-GENE-040718-375	yes
IE-091204-306]	yes
Acc:HGNC:1065]	yes
IE-050208-235]	yes
3-GENE-121116-7]	yes
NE-060825-45]	yes
ATPase sarcoplasmic/endoplasmic reticulum Ca2+ transporting 2a [Source	yes
ATPase phospholipid transporting 11A [Source:HGNC Symbol;Acc:HGNC:1	yes
[Source:ZFIN;Acc:ZDB-GENE-050517-16]	yes
	yes
	yes
26-2311]	yes
2]	yes
	yes
ce:ZFIN;Acc:ZDB-GENE-000509-2]	yes
041212-33]	yes

ariadne homolog 2 (Drosophila) [Source:ZFIN;Acc:ZDB-GENE-040426-215	yes
Source:ZFIN;Acc:ZDB-GENE-050913-39]	yes
n 3 [Source:ZFIN;Acc:ZDB-GENE-091019-1]	yes
c:ZDB-GENE-040426-1189]	yes
	yes
	yes
aldehyde dehydrogenase 18 family, member A1 [Source:ZFIN;Acc:ZDB-GE	yes
N;Acc:ZDB-GENE-040120-5]	yes
E-080516-10]	yes
E-030131-9370]	yes
DB-GENE-040624-6]	yes
90422-1]	yes
:ZFIN;Acc:ZDB-GENE-040426-1103]	yes
ce:ZFIN;Acc:ZDB-GENE-030131-3187]	yes
nd leucine zipper containing 1 [Source:ZFIN;Acc:ZDB-GENE-081016-1]	yes
Acc:HGNC:15479]	yes
GENE-070810-1]	yes
Acc:HGNC:195]	yes
104-428]	yes
acyl-CoA dehydrogenase short/branched chain [Source:ZFIN;Acc:ZDB-GEN	yes
acyl-CoA dehydrogenase long chain [Source:ZFIN;Acc:ZDB-GENE-040426-	yes
N;Acc:ZDB-GENE-070912-325]	yes
GENE-010601-2]	yes
ATP-binding cassette, sub-family B (MDR/TAP), member 11a [Source:ZFIN	yes
ATP-binding cassette, sub-family A (ABC1), member 4a [Source:ZFIN;Acc:Z	yes
apoptosis-associated tyrosine kinase a [Source:ZFIN;Acc:ZDB-GENE-10081	yes
AP2 associated kinase 1a [Source:ZFIN;Acc:ZDB-GENE-081105-101]	yes
NE-090804-1]	yes
ENE-050327-29]	yes
methionyl-tRNA synthetase 2, mitochondrial [Source:ZFIN;Acc:ZDB-GENE-	yes
494]	yes
FIN;Acc:ZDB-GENE-030131-1673]	yes
	yes
	yes
	yes
	yes
	yes
	yes
	yes
	yes
	yes
	yes
	yes
	yes
	yes
	yes
	yes
	yes
	yes
	yes
	yes
	yes

yes
yes
yes
yes
yes
yes
yes
yes
yes
yes
yes
yes
yes
yes
yes
yes
yes
yes
yes
yes
yes
yes
yes
yes
yes
yes
yes
yes
yes
yes
yes
yes
yes
yes
yes
yes
yes
yes
yes
yes
yes
yes
yes
yes
yes
yes
yes
yes
yes
yes
yes
yes

	yes
	yes
	yes
	yes
	yes
	yes
	yes
	yes
	yes
	yes
	yes
	yes
	yes
	yes
	yes
	yes
	yes
	yes
	yes
	yes
	yes
	yes
	yes
	yes
	yes
	yes
	yes
	yes
	yes
	yes
	yes
	yes
	yes
	yes
	yes
	yes
	yes
	yes
	yes
ATPase sarcoplasmic/endoplasmic reticulum Ca2+ transporting 2a [Source	no
ATPase sarcoplasmic/endoplasmic reticulum Ca2+ transporting 2a [Source	no
ATP-binding cassette, sub-family B (MDR/TAP), member 11a [Source:ZFIN	no
ceruloplasmin [Source:ZFIN;Acc:ZDB-GENE-010522-1]	no
ectonucleotide pyrophosphatase/phosphodiesterase 2 [Source:HGNC Sym	no
endoplasmic reticulum aminopeptidase 2 [Source:ZFIN;Acc:ZDB-GENE-08	no
glucosaminyl (N-acetyl) transferase family member 7 [Source:ZFIN;Acc:ZD	no
lipoxygenase homology domains 1a [Source:ZFIN;Acc:ZDB-GENE-091112-	no
molybdenum cofactor synthesis 3 [Source:ZFIN;Acc:ZDB-GENE-040426-78	no
phospholipase C zeta 1 [Source:HGNC Symbol;Acc:HGNC:19218]	no
protein kinase, AMP-activated, alpha 1 catalytic subunit [Source:ZFIN;Acc	no
pyrophosphatase (inorganic) 1a [Source:ZFIN;Acc:ZDB-GENE-040426-169	no
tyrosinase [Source:ZFIN;Acc:ZDB-GENE-991026-3]	no
70228-4]	no

IN;Acc:ZDB-GENE-090310-1]	no
DB-GENE-130425-3]	no
DB-GENE-130425-3]	no
HGNC:2194]	no
-040421-2]	no
-130530-765]	no
GENE-040426-1678]	no
NE-060825-45]	no
NE-060825-45]	no
GNC Symbol;Acc:HGNC:2622]	no
	no
B-GENE-040718-250]	no
cc:ZDB-GENE-081119-4]	no
cc:ZDB-GENE-081119-4]	no
GENE-140303-1]	no
	no
30131-9786]	no
	no
	no
	no
	no
cc:ZDB-GENE-090506-7]	no
nber 3a [Source:ZFIN;Acc:ZDB-GENE-040718-390]	no
siae) [Source:ZFIN;Acc:ZDB-GENE-040426-2411]	no
DB-GENE-060503-418]	no
NE-110203-5]	no
	no
	no
	no
	no
	no
	no
rce:ZFIN;Acc:ZDB-GENE-040426-1012]	no
NC:8543]	no
9-65]	no
	no
ce:HGNC Symbol;Acc:HGNC:18549]	no
	no
	no
HGNC Symbol;Acc:HGNC:1316]	no
6-2846]	no
6-2846]	no
r 6, like [Source:ZFIN;Acc:ZDB-GENE-040426-2249]	no
rce:ZFIN;Acc:ZDB-GENE-041114-118]	no
DB-GENE-070410-31]	no
:HGNC:11009]	no
110512-1]	no
ne domains 2 [Source:ZFIN;Acc:ZDB-GENE-050522-160]	no
]	no
FIN;Acc:ZDB-GENE-030131-3208]	no
FIN;Acc:ZDB-GENE-030131-3208]	no
	no

	no
3-GENE-070719-5]	no
	no
	no
reductase [Source:ZFIN;Acc:ZDB-GENE-101115-3]	no
er 9 [Source:ZFIN;Acc:ZDB-GENE-060421-6224]	no
	no
C:3179]	no
	no
5a [Source:ZFIN;Acc:ZDB-GENE-040426-2049]	no
:ZFIN;Acc:ZDB-GENE-030131-6186]	no
mRNA. [Source:RefSeq mRNA;Acc:NM_001104691]	no
ymbol;Acc:HGNC:26977]	no
ember 1 [Source:ZFIN;Acc:ZDB-GENE-070830-1]	no
	no
120-114]	no
ol;Acc:HGNC:13534]	no
Acc:HGNC:1065]	no
N;Acc:ZDB-GENE-141215-19]	no
ce:ZFIN;Acc:ZDB-GENE-030131-3187]	no
GENE-041111-91]	no
417-71]	no
ember 2b [Source:ZFIN;Acc:ZDB-GENE-041210-312]	no
efSeq mRNA;Acc:NM_001122902]	no
05-120]	no
	no
	no
	no
	no
	no
	no
	no
IN;Acc:ZDB-GENE-070122-2]	no
c:ZDB-GENE-120628-1]	no
GENE-050208-783]	no
	no
29167]	no
Source:ZFIN;Acc:ZDB-GENE-061110-98]	no
tor of chromatin, subfamily d, member 1 [Source:ZFIN;Acc:ZDB-GENE-030	no
Acc:ZDB-GENE-030131-6098]	no
) transferase [Source:ZFIN;Acc:ZDB-GENE-120813-8]	no
	no
51]	no
;Acc:HGNC:29173]	no
nit, a [Source:ZFIN;Acc:ZDB-GENE-090514-1]	no
IE-030131-6138]	no
28-4]	no
nit, b [Source:ZFIN;Acc:ZDB-GENE-051227-1]	no
nit, b [Source:ZFIN;Acc:ZDB-GENE-051227-1]	no
member 1b [Source:ZFIN;Acc:ZDB-GENE-070424-31]	no
member 1b [Source:ZFIN;Acc:ZDB-GENE-070424-31]	no
member 1b [Source:ZFIN;Acc:ZDB-GENE-070424-31]	no

	no
ber 6b [Source:ZFIN;Acc:ZDB-GENE-030131-3077]	no
ENE-090410-3]	no
ENE-090410-3]	no
	no
	no
	no
	no
	no
03-743]	no
urce:ZFIN;Acc:ZDB-GENE-140619-2]	no
ember 3 [Source:ZFIN;Acc:ZDB-GENE-060531-95]	no
ember 3 [Source:ZFIN;Acc:ZDB-GENE-060531-95]	no
E-060531-7]	no
E-060531-7]	no
nber 2 [Source:ZFIN;Acc:ZDB-GENE-030131-1213]	no
-081114-2]	no
c:HGNC:11862]	no
NE-000208-18]	no
426-2426]	no
426-2426]	no
426-2426]	no
B-GENE-040426-833]	no
:ZDB-GENE-120215-261]	no
Acc:ZDB-GENE-071012-5]	no
091204-268]	no
C Symbol;Acc:HGNC:4807]	no
	no
	no
	no
NE-000208-18]	no
GENE-010601-2]	no
1]	no
:ZFIN;Acc:ZDB-GENE-040426-1103]	no
718-149]	no
c:HGNC:13544]	no
:HGNC Symbol;Acc:HGNC:9311]	no
	no
c:ZDB-GENE-040715-2]	no
ENE-050327-29]	no
715]	no
	no
GENE-131121-49]	no
	no
	no
	no
	no
	no
	no
	no
	no
	no

	no
	no
.cc:ZDB-GENE-040724-171]	no
Acc:ZDB-GENE-030131-1747]	no
E-020228-2]	no
	no
GENE-050417-122]	no
	no
HGNC:13840]	no
20813-1]	no
20813-1]	no
urce:ZFIN;Acc:ZDB-GENE-040426-933]	no
Acc:ZDB-GENE-031112-1]	no
GENE-041114-196]	no
.cc:ZDB-GENE-041210-103]	no
ng 2b [Source:ZFIN;Acc:ZDB-GENE-121214-111]	no
ol;Acc:HGNC:30268]	no
nsporter 2) [Source:ZFIN;Acc:ZDB-GENE-051113-248]	no
E-080402-9]	no
3-10]	no
er 7 [Source:ZFIN;Acc:ZDB-GENE-051113-336]	no
Source:ZFIN;Acc:ZDB-GENE-030131-664]	no
	no
	no
	no
	no
	no
	no
	no
.08-4]	no
3-7]	no
	no
	no
	no
	no
ENE-050417-155]	no
ENE-050417-155]	no
l;Acc:ZDB-GENE-030404-1]	no
:ZDB-GENE-000616-8]	no
	no
DB-GENE-041008-138]	no
5]	no
C Symbol;Acc:HGNC:10591]	no
	no
	no
	no
	no
	no
	no
