# Supplementary material for: MiR-202 controls female fecundity by regulating medaka oogenesis
Source: PLoS Genet. 2018 Sep 10;14(9):e1007593. doi: 10.1371/journal.pgen.1007593 (PMC6147661; doi:10.1371/journal.pgen.1007593)
Supplement: S3 Table — (PDF) [file pgen.1007593.s006.pdf]

S3 Table. Primers used for HIDI-PCR

Name	Sequence (5'-->3')
Genot_HIDI_MiR202_F	CAACCAGTCAATGCACATGAT
Genot_HIDI_MiR202WT_R	ACCTCTGCAGCCCCATTTTC
Genot_HIDI_MiR202Mut_R	AAAACCTCTGCAGCCCATG
