# Supplementary material for: MiR-202 controls female fecundity by regulating medaka oogenesis
Source: PLoS Genet. 2018 Sep 10;14(9):e1007593. doi: 10.1371/journal.pgen.1007593 (PMC6147661; doi:10.1371/journal.pgen.1007593)
Supplement: S4 Table — (PDF) [file pgen.1007593.s007.pdf]

S4 Table. Primers used for qRT-PCR

Gene name	Sequence (5’ --> 3’)
RPL7_F	GAGATCCGCCTGGCTCGTA
RPL7_R	GGGCTGACTCCGTTGATACCT
Wnt2b_F	GCACCAGTTCAGACACCATC
Wnt2b_R	TGCTTCTCGACTGCTTCTGA
Wnt4_F	AGTGTCATGGAGTGTCTGGG
Wnt4_R	CCTCAGTGGCACCATCAAAC
Clockb_F	CGTCAACAACCAGCAGTGAA
Clockb_R	AGTGGAGAATTGAGGCAGCT
Setd4_F	CTACCTGGGGCCGTTCTTAA
Setd4_R	CGCACACCAGAAAGACACAA
Klhl23_F	ATGATGGGAAGCTGAGGTCC
Klhl23_R	GGGTGCCAGTAGTATCCTCC
Npr1_F	TGAAGATGCCACGGTACTGT
Npr1_R	AGCAGACACGTGGATCTTCA
Srgap3_F	GCACACCACATTCAGCAGAT
Srgap3_R	CCATCATGTTCTCGTCGCTG
Cyp19a1a_F	CTCTTCCTGGGTGTTCCTGTTG
Cyp19a1a_R	GCTGCTGTCTTGTGCCTCTG
Cyp17_F	AGTGACACCAGCCTCGGAGA
Cyp17_R	GGTCCACTCCTTCTCATCGTG
Gsdf_F	GGGCTGGACACTATTCGAGA
Gsdf_R	CATGACACAGAGGAGCTGGA
Sox9b_F	AGCGACTCCAAGAAGGACGA
Sox9b_R	GTCCAGTCGTAGCCCTTCAG
Foxl2_F	GTCACAAACCACAACCTGCT
Foxl2_R	TTTGGAGCCGTTTGTCATCC
Foxl3_F	CAAAGCCCACCTGAGTCATG
Foxl3_R	AGAGCCACGTACGAATAGGG
Vasa_F	CCCAAAGTGACCTACATC
Vasa_R	AAGTTGATGCCCATCTTG
Inh_F	CGTTTCCCTTCCAGCCTTC
Inh_R	AAGAGCGTTGCGGATGAG
Sycp3_F	ACTTTAGTGGCGGGAAGACG
Sycp3_R	GCACATTCATCCGCTCCTTC
ControlGeneUp_F	ACTCTGGGTCTCATCTGCAC
ControlGeneUp_R	GAGTCACAGCAGGTTCAGGA
